# Resin Glycosides with *α*-Glucosidase and Protein Tyrosine Phosphatase 1B Inhibitory Activities from the Seeds of *Cuscuta japonica*

**DOI:** 10.3390/biom15101465

**Published:** 2025-10-16

**Authors:** Su-Peng Guo, Ye He, Xin Lan, Tian-Zi Qi, Jin-Ping Gu, Jia Guo, Xin-Yu Wang, Min Yang, Wen-Li Wang, Guang-Tong Chen, Bo-Yi Fan

**Affiliations:** 1 School of Pharmacy, Nantong University, 9 Seyuan Road, Nantong 226019, China; 2 Jinghua Pharmaceutical Group Co., Ltd., 9 Xingtai Road, Nantong 226005, China

**Keywords:** resin glycoside, *Cuscuta japonica*, *α*-glucosidase inhibitory activity, PTP1B inhibitory activity, molecular docking

## Abstract

In the present study, seven previously undescribed resin glycosides, designated cusponins I-VII (**1**–**7**), together with one known analog (**8**), were isolated from the seeds of *Cuscuta japonica*, a traditional medicine used in China. Structural elucidation revealed them to be glycosidic acid methyl esters, generated through on-column methyl esterification of naturally occurring resin glycosides catalyzed by NH_2_-functionalized silica gel. All isolates were characterized as either pentasaccharides or tetrasaccharides, incorporating D-glucose, L-rhamnose, or D-fucose units as the sugar residues. Notably, compounds **1** and **3**–**7** contained the uncommon aglycone, 11*S*-hydroxypentadecanoic acid. Bioactivity assessments demonstrated that compounds **1**–**4**, **6** and **8** suppressed *α*-glucosidase activity, with IC_50_ values between 8.02 and 71.39 μM. In addition, compounds **3** and **5** exhibited inhibitory effects on protein tyrosine phosphatase 1B (PTP1B), with IC_50_ values of 14.19 ± 1.29 μM and 62.31 ± 8.61 μM, respectively, marking the first report of PTP1B inhibitory activity among resin glycosides. Enzyme kinetic analyses indicated that compound **2** acted as an uncompetitive *α*-glucosidase inhibitor (*K*_is_ = 3.02 μM), whereas compound **3** inhibited PTP1B via a mixed-type mechanism (*K*ᵢ = 24.82 μM; *K*_is_ = 64.24 μM). Molecular docking combined with molecular dynamics simulations suggested that compounds **2** and **3** interacted with *α*-glucosidase-pNPG and PTP1B, respectively, forming stable complexes with favorable binding free energies. Collectively, this study reported eight resin glycosides from *C. japonica*, seven of them newly identified, with compounds **2** and **3** highlighted as promising scaffolds for antidiabetic drug discovery.

## 1. Introduction

Diabetes mellitus is a prevalent and complex metabolic disorder, defined by sustained elevations in blood glucose levels and frequently accompanied by a spectrum of long-term complications, notably those affecting the cardiovascular system, retinal function, and renal health [1,2]. Based on the combined assessments of the World Health Organization and the International Diabetes Federation, an estimated 537 million individuals worldwide were living with diabetes in 2021, and this figure is projected to surge to approximately 783 million by 2045 [3]. Among the various forms of this disease, type 2 diabetes mellitus (T2DM), distinguished by the gradual loss of pancreatic *β*-cell function in conjunction with reduced tissue sensitivity to insulin, remains the most widespread and burdensome [4]. A key player in postprandial glucose regulation is *α*-glucosidase, a digestive enzyme embedded in the brush-border membranes of small intestinal epithelial cells, which catalyzes the conversion of dietary oligo- and disaccharides into absorbable monosaccharides. Pharmacological inhibition of *α*-glucosidase slows carbohydrate breakdown, thereby mitigating sharp rises in blood glucose and offering a preventive approach to T2DM [5,6]. Equally important is protein tyrosine phosphatase 1B (PTP1B), a pivotal intracellular non-transmembrane enzyme that exerts a suppressive effect on insulin signaling pathways: selective inhibition of the activity of PTP1B enhances cellular glucose uptake and counteracts the insulin resistance that typifies T2DM [7]. Consequently, the identification and development of novel therapeutic agents capable of targeting either *α*-glucosidase or PTP1B has emerged as an area of intense interest and strategic significance to both biomedical research and the pharmaceutical industry.

Resin glycosides, hallmark metabolites of plants in the family *Convolvulaceae*, represent a unique and structurally intricate class of glycolipids. Owing to their remarkable structural diversity and wide-ranging biological profiles, these compounds have garnered considerable attention from both natural products chemists and pharmacologists [8,9]. Structurally, a typical resin glycoside features an oligosaccharide moiety covalently linked to a long-chain fatty acid aglycone [8,9]. To date, hundreds of resin glycosides have been isolated from members of the Convolvulaceae, many of which display an extensive spectrum of pharmacological activities, such as cytotoxic, anti-inflammatory, sedative, antiviral, anticonvulsant, and multidrug-resistance reversal effects [10,11,12,13,14,15]. Notably, several resin glycosides have also been reported as *α*-glucosidase inhibitors. For example, six cairicoside resin glycosides purified from *Ipomoea cairica* have demonstrated pronounced *α*-glucosidase inhibitory properties, with IC_50_ values ranging from 21.4 to 30.4 μM [16,17]. Likewise, albinosides VI and VII, obtained from *Ipomoeaq alba* via an affinity-directed fractionation-mass spectrometry approach, have been identified as potent *α*-glucosidase inhibitors [18]. Furthermore, operculinic acid A from *Ipomoea biflora* exhibits notable *α*-glucosidase inhibition, recording an IC_50_ of 78.32 μM [19].

*Cuscuta japonica* Choisy, commonly referred to as Japanese dodder, is a parasitic vine that derives water and nutrients directly from the its host plants [20]. In traditional Chinese medicine, the seeds of *C. japonica* are employed as substitutes for Cuscutae Semen, a renowned herbal remedy traditionally used to alleviate excessive urination, enhance sexual vitality, and manage conditions such as pharyngitis [21]. Contemporary pharmacological investigations have demonstrated that *C. japonica* seeds can shield SH-SY5Y neuronal cells from H_2_O_2_-induced oxidative damage, suppress influenza virus infection and HIV-1 fusion, improve cognitive performance, and inhibit α-MSH-stimulated melanogenesis [20,21,22,23,24]. Phytochemical analyses have further revealed a diverse repertoire of secondary metabolites, including aromatic glycosides, flavonoids, steroids, alkaloids, fatty acids, and lignans [20,21,25]. Notably, to the best of our knowledge, resin glycosides have not previously been reported from this species.

**Figure 1:**
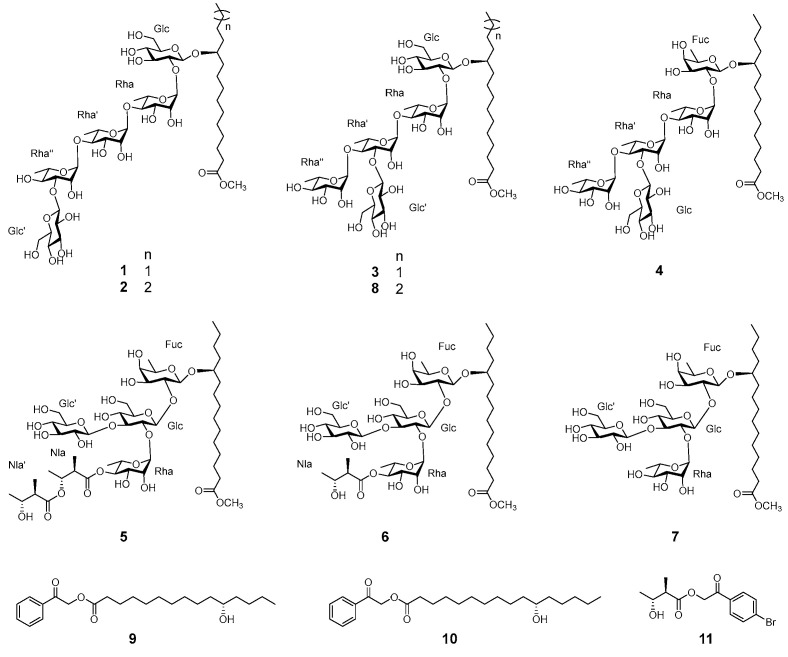
Chemical structures of compounds **1**–**8** and related derivatives **9**–**11**.

As our continuing research exploration on structurally and functionally diverse resin glycosides from Convolvulaceae plants [26,27,28], the seeds of *C. japonica* were investigated. Although the initial isolation process was hindered by poor high-performance liquid chromatography (HPLC) resolution, seven previously undescribed glycosidic acid methyl esters (**1**–**7**) and one known analog (**8**) were successfully obtained through NH_2_–silica gel on-column methyl esterification (Figure 1) [26,27]. Structural characterization established that these compounds were either pentasaccharides or tetrasaccharides, composed of D-glucose, L-rhamnose, or D-fucose residues, with compounds **1** and **3**–**7** featuring the rare 11*S*-hydroxypentadecanoic acid as the aglycone [29]. The α-glucosidase and PTP1B inhibitory activities of all eight compounds were assessed, alongside kinetic analyses, molecular docking and molecular dynamics (MD) simulations for the bioactive members. This study thus detailed the isolation, structural elucidation, and bioactivity evaluation of compounds **1**–**8** from *C. japonica* seeds were reported.

## 2. Materials and Methods

### 2.1. General Experimental Procedures

Optical rotation values were determined using an Anton Paar MCP 150 polarimeter (Anton Paar Group, Graz, Austria). Infrared (IR) spectra were recorded on a Thermo Nicolet iS10 IR spectrometer (Thermo Fisher Scientific Inc., Waltham, MA, USA), and high-resolution electrospray ionization mass spectrometry (HRESIMS) data were obtained on a Waters G2-S Q-TOF LC/MS system (Waters Corp., Milford, MA, USA). A Bruker 600 AV NEO spectrometer (Bruker Corp., Billerica, MA, USA) was used to acquire Nuclear magnetic resonance (NMR) spectra. For column chromatography, silica gel (Qingdao Marine Chemical Co., Ltd., Qingdao, China), NH_2_-silica gel (Wuhan Weiqi Technology, Wuhan, China), Sephadex LH-20 (GE Healthcare Bio-Sciences AB Inc., Uppsala, Sweden), and octadecylsilyl (ODS, 40–63 μm, YMC Co., Ltd., Kyoto, Japan) materials were employed. A Shimadzu LC-20AD liquid chromatograph (Shimadzu Corp., Tokyo, Japan) was used for preparative HPLC, monitored with either an RID-10A refractive index detector or a SPD-20A UV/VIS detector, and fitted with a YMC RP-C18 column (10 × 200 mm).

### 2.2. Plant Material

Seeds of *C. japonica* were harvested in August 2021 from Nanning City, Guangxi Zhuang Autonomous Region, China. Botanical identification was performed by Prof. Min Yang, and a voucher specimen (No. 202108) has been deposited in the School of Pharmacy, Nantong University.

### 2.3. Extraction and Isolation

The powdered seeds of *C. japonica* (10.0 kg) were refluxed with 95% EtOH for three successive cycles (3 × 3 h). The combined EtOH extracts were concentrated under reduced pressure to yield a crude residue (700 g), which was suspended in water and successively partitioned with dichloromethane (CH_2_Cl_2_) and n-butanol (n-BuOH). The n-BuOH-soluble fraction was subjected to silica gel column chromatography, eluted stepwise with CH_2_Cl_2_/MeOH mixtures (10:1, 5:1, 2:1, 1:1, *v*/*v*), affording four major fractions (Frs. 1–4). Fr. 3 (24.3 g) was further separated over an ODS column using a gradient of MeOH-H_2_O (30:70, 50:50, 70:30, 80:20, 90:10, 100:0, *v*/*v*) to produce five subfractions (Frs. 3.1–3.5). Based on diagnostic ^1^H NMR signals and TLC analysis, Fr. 3.2 was identified as the principal resin glycoside-containing fraction.

Fr. 3.2 was initially purified by the Sephadex LH-20 column chromatography with MeOH as the eluent, followed by NH_2_-silica gel column chromatography eluted with CH_2_Cl_2_/MeOH mixtures (5:1, 2:1, 1:1, 0:1, *v*/*v*) to produce the catalyzed resin glycoside fraction (Fr. 3.2.1). This fraction was further separated on an ODS column eluted with MeOH-H_2_O gradients (70:30, 80:20, 90:10, 100:0, *v*/*v*), generating seven subfractions (Frs. 3.2.1.1–3.2.1.7).

Preparative HPLC of Fr. 3.2.1.1, using MeOH–H_2_O (70:30, *v*/*v*) as the mobile phase, afforded compounds **1** (*t*_R_ = 17 min, 1.6 mg), and **3** (*t*_R_ = 20 min, 1.7 mg). Similarly, purification of Fr. 3.2.1.2 with MeOH-H_2_O (70:30, *v*/*v*) yielded compounds **2** (*t*_R_ = 30 min, 1.7 mg), **4** (*t*_R_ = 17 min, 2.4 mg), and **8** (*t*_R_ = 14 min, 2.6 mg). Using MeOH-H_2_O (89:11, *v*/*v*) as the HPLC eluent, compounds **5** (*t*_R_ = 50 min, 3.4 mg), **6** (*t*_R_ = 34 min, 7.6 mg), and **7** (*t*_R_ = 29 min, 3.7 mg) were isolated from Fr. 3.2.1.4.

Cusponin I (**1**): colorless gum; [*α*]^20^_D_ −22.5 (c 0.03, MeOH); IR *v*_max_ (KBr) cm^−1^: 3429.16, 2930.18, 1632.86, 1067.34; ^1^H and ^13^C NMR spectral data: see Table 1; HRESIMS *m*/*z* 1033.5062 [M−H]^−^ (calcd for C_46_H_81_O_25_ 1033.5067);

Cusponin II (**2**): colorless gum; [*α*]^20^_D_ −65.0 (c 0.04, MeOH); IR *v*_max_ (KBr) cm^−1^: 3441.07, 2931.92, 1630.15, 1083.79; ^1^H and ^13^C NMR spectral data: see Table 1; HRESIMS *m*/*z* 1071.5189 [M + Na]^+^ (calcd for C_47_H_84_O_25_Na 1071.5199);

Cusponin III (**3**): colorless gum; [*α*]^20^ _D_ −42.5 (c 0.04, MeOH); IR *v*_max_ (KBr) cm^−1^: 3431.26, 2933.22, 1628.07, 1062.75; ^1^H and ^13^C NMR spectral data: see Table 1; HRESIMS *m*/*z* 1057.5035 [M + Na]^+^ (calcd for C_46_H_82_O_25_Na 1057.5043);

Cusponin IV (**4**): colorless gum; [*α*] ^20^_D_ −26.8 (c 0.04, MeOH); IR *v*_max_ (KBr) cm^−1^: 3430.83, 2932.71, 1725.98, 1654.62, 1044.67; ^1^H and ^13^C NMR spectral data: see Table 1; HRESIMS *m*/*z* 1041.5096 [M + Na]^+^ (calcd for C_46_H_82_O_24_Na 1041.5094).

Cusponin V (**5**): colorless gum; [*α*]^20^_D_ −25.0 (c 0.04, MeOH); IR *v*_max_ (KBr) cm^−1^: 3427.64, 2926.33, 1737.15, 1074.76; ^1^H and ^13^C NMR spectral data: see Table 2; HRESIMS *m*/*z* 1111.5515 [M + Na]+ (calcd for C_50_H_88_O_25_Na 1111.5512);

Cusponin VI (**6**): colorless gum; [*α*]^20^_D_ −14.6 (c 0.06, MeOH); IR *v*_max_ (KBr) cm^−1^: 3430.23, 2926.22, 1742.94, 1074.16; ^1^H and ^13^C NMR spectral data: see Table 2; HRESIMS *m*/*z* 1011.4988 [M + Na]+ (calcd for C_45_H_80_O_23_Na 1011.4988);

Cusponin VII (**7**): colorless gum; [*α*]^20^_D_ −16.7 (c 0.06, MeOH); IR *v*_max_ (KBr) cm^−1^: 3414.50, 2928.61, 2854.62, 1622.54, 1075.73; ^1^H and ^13^C NMR spectral data: see Table 2; HRESIMS *m*/*z* 911.4465 [M + Na]^+^ (calcd for C_40_H_72_O_21_Na 911.4464).

### 2.4. Alkaline Hydrolysis of Resin Glycoside Fraction

A portion of the resin glycoside fraction Fr 3.2 was dissolved in 5% NaOH solution and subjected to be refluxed at 90 °C for 2 h. After completion, the reaction mixture was cooled to ambient temperature, acidified to pH 4 using HCl, and sequentially extracted with CH_2_Cl_2_, ethyl acetate (EtOAc), and n-BuOH, successively.

The EtOAC fraction was evaporated to dryness and reacted with *p*-bromophenacyl bromide (10 mg) in anhydrous acetone (2 mL), added with several drops of triethylamine, at room temperature for 1 h. Purification of the reaction mixture was carried out by preparative HPLC using MeOH-H_2_O (64:36, *v*/*v*) as the mobile phase, yielding 4-bromophenyacyl-(2*R*,3*R*)-nilate (**11**, t_R_ = 35 min, 1.2 mg). The structure of compound **11** was confirmed by comparing its NMR, HRESIMS, and optical rotation data with those of an authentic sample [26].

### 2.5. Acid Hydrolysis and Sugar Analysis

The aforementioned n-BuOH fraction was dried under reduced pressure, and was added with 10 mL of 1N HCl, followed by heating at 90 °C for 2 h. After it was cooled to room temperature, the reaction mixture underwent successive extractions with CH_2_Cl_2_ and EtOAc.

The CH_2_Cl_2_ layer was reacted with 2-bromoacetophenone following previously reported protocols [26,28]. Subsequent purification of the reaction mixture was carried out by preparative HPLC with MeOH-H_2_O (83:17, *v*/*v*), affording 2-oxo-2-phenylethyl 11*S*-hydroxypentadecanoate (**9**, *t*_R_ = 27 min, 4.7 mg), and 2-oxo-2-phenylethyl 11*S*-hydroxyhexadecanoate (**10**, *t*_R_ = 33 min, 1.5 mg), Structural assignments of compounds **9** and **10** were confirmed by NMR, HRESIMS, and optical rotation data comparisons with literature values [28,29].

The aqueous phase, consisting of hydrolyzed sugars, was derived to determine their absolute configurations using established procedures [26,28]. The resulting derivatives were analyzed on a Shimadzu HPLC system equipped with a Welch Ultimate XB-C18 column (4.6 × 150 mm), maintained at 30 °C. 25% Acetonitrile in 0.1% formic acid solution was used as the mobile phase at a flow rate of 1.0 mL/min, and detection was carried out at 254 nm. Retention times of the derivatives (9.7, 13.2, and 17.3 min) were compared with standards, confirming the presence of D-glucose, D-fucose, and L-rhamnose, respectively.

### 2.6. α-Glucosidase Inhibitory Assay

All the compounds **1**–**8** were evaluated for their inhibitory effects against *α*-glucosidase derived from *Saccharomyces cerevisiae*. In brief, 140 μL of phosphate buffer (0.2 M, pH 6.8) was added to the 96-well microplate wells, followed by the addition of 10 μL *α*-glucosidase solution (0.5 U/mL, Sigma-Aldrich Inc., Saint Louis, MO, USA) and 2 μL of the test compound. The control wells contained 2 μL of DMSO in place of the test sample, while the enzyme solution was replaced by an additional 10 μL of phosphate buffer in the background wells. After preincubation at 37 °C for 10 min, 48 μL of 2 mM p-nitrophenyl-*α*-D-glucopyranoside (pNPG) was introduced into each well. The reaction mixtures were incubated for 30 min before being terminated by adding 50 μL of 0.1 M Na_2_CO_3_ solution. Absorbance was measured at 405 nm using a microplate reader. Genistein served as the positive control. All assays were performed in triplicate and repeated three times independently [19].

### 2.7. PTP1B Inhibitory Assay

The inhibitory activity against PTP1B was assessed via the p-nitrophenyl phosphate (pNPP) hydrolysis assay. The reaction buffer consisted of 50 mM citrate buffer (pH 6.0) supplemented with 0.1 M NaCl, 2 mM EDTA, and 1 mM DTT. Recombinant PTP1B enzyme (MedChemExpress LLC, Monmouth Junction, NJ, USA) was used at a concentration of 5 μg/mL, with 10 mM pNPP serving as the substrate. The enzymatic reaction was terminated by the addition of 1M NaOH. Experimental procedures mirrored those used in the *α*-glucosidase assay, and ursolic acid was employed as the positive control [30].

### 2.8. Enzyme Reversibility and Kinetics Analysis

To elucidate the inhibitory mechanisms of the most potent α-glucosidase inhibitor, compound **2**, and PTP1B inhibitor, compound **3**, enzyme reversibility and kinetic analyses were performed. Reversibility was assessed by plotting the enzyme reaction velocity (v) against varying enzyme concentrations in the presence of different inhibitor concentrations. Lineweaver–Burk plots (1/v vs. 1/[S]) were constructed by measuring reaction velocities at multiple substrate concentrations with varying amounts of inhibitors. From these plots, inhibition type, as well as Michaelis–Menten constants *K*_m_ and *V*_max_, were determined. Additionally, slopes and Y-intercepts of these reciprocal plots were also plotted against inhibitor concentrations to further characterize the inhibition constants *K*_i_ and *K*_is_, respectively [6].

### 2.9. Molecular Docking Analysis

The amino acid sequence of *α*-glucosidase AL12 from *Saccharomyces cerevisiae* (UniProt accession number P53341) was obtained from the UniProt protein database. The I-TASSER online server was utilized for homology modeling of *α*-glucosidase [19,31]. The crystal structure of PTP1B was retrieved from the Protein Data Bank (PDB ID: 2QBQ) [32]. Ligand 3D structures of ligands were constructed and energy minimized using the MMFF94 force field. AutoDock Vina version 1.1.2 (Scripps Research, La Jolla, CA, USA) was used for the molecular docking simulations were via flexible docking approach. Visualization of ligand–protein interactions was generated with PyMOL (Schrödinger Inc., New York, NY, USA) for three-dimensional diagrams and Discovery Studio (Dassault Systèmes, CA, USA) for two-dimensional interaction maps.

### 2.10. MD Simulation

MD simulations were performed using GROMACS version 2022.03 software (University of Groningen, Groningen, Netherlands) [33]. The protein topology was generated with the AMBER99SB-ILDN force field integrated within GROMACS, whereas ligand topology and coordinate files were prepared using AmberTools22. The protein–ligand complex was solvated in a TIP3P water model with a minimum distance of 1.2 nm between protein atoms and the edges of the cubic water box. To neutralize the system, Na^+^ ions were added accordingly. Energy minimization was conducted using the steepest descent algorithm to achieve a stable starting conformation. Short-range electrostatic and van der Waals interactions employed a cutoff of 1.0 nm, while long-range electrostatics were treated via the Particle Mesh Ewald (PME) method. All hydrogen-containing bonds were constrained using the LINCS algorithm.

During the MD simulation process, pressure was regulated at 1 bar employing the Berendsen barostat, and temperature control was maintained at 300 K using the V-rescale thermostat. The system underwent initial energy equilibration, starting with a 100 ps pre-equilibration, followed by sequential 100 ps simulations in the NVT and NPT ensembles to stabilize temperature and pressure, respectively. Subsequently, production MD runs of 100 ns duration were carried out without constraints. The simulations were conducted under periodic boundary conditions within an NPT ensemble. Integration time steps were set to 2 fs, with trajectory data, including atomic coordinates, velocities, and energies, recorded every 10 ps for downstream analyses.

## 3. Results and Discussion

### 3.1. Structural Elucidation

Cusponin I (**1**) was isolated as a colorless gum, and its molecular formula was determined as C_46_H_82_O_25_ based on the HRESIMS ion peak at m/z 1033.5062 [M − H]^−^ (calcd for C_46_H_81_O_25_, 1033.5067). The ^1^H NMR spectrum (Table 1) displayed five anomeric proton signals at δ_H_ 4.97 (d, J = 7.2 Hz), 6.41 (br s), 6.25 (br s), 6.30 (br s), and 5.38 (d, J = 7.8 Hz), along with a methoxy proton resonance at δ_H_ 3.64 (s) and a primary methyl signal at δ_H_ 1.04 (t, J = 7.2 Hz). Correspondingly, the ^13^C NMR spectrum (Table 1) revealed five anomeric carbons at δ_C_ 101.5, 101.8, 103.5, 103.7, and 107.3, a methoxy carbon at δ_c_ 51.7 and a carboxyl carbon resonating at δ_C_ 174.5. Additional overlapping signals between δ_H_ 3.6–5.2 and δ_C_ 62–85 were assigned to sugar residues, while resonances between δ_H_ 1.1–2.4 and δ_C_ 14–36 corresponded to fatty acid moieties. Collectively, these data indicated that compound **1** was a pentasaccharide glycosidic acid methyl ester [26,27].

**Figure 2:**
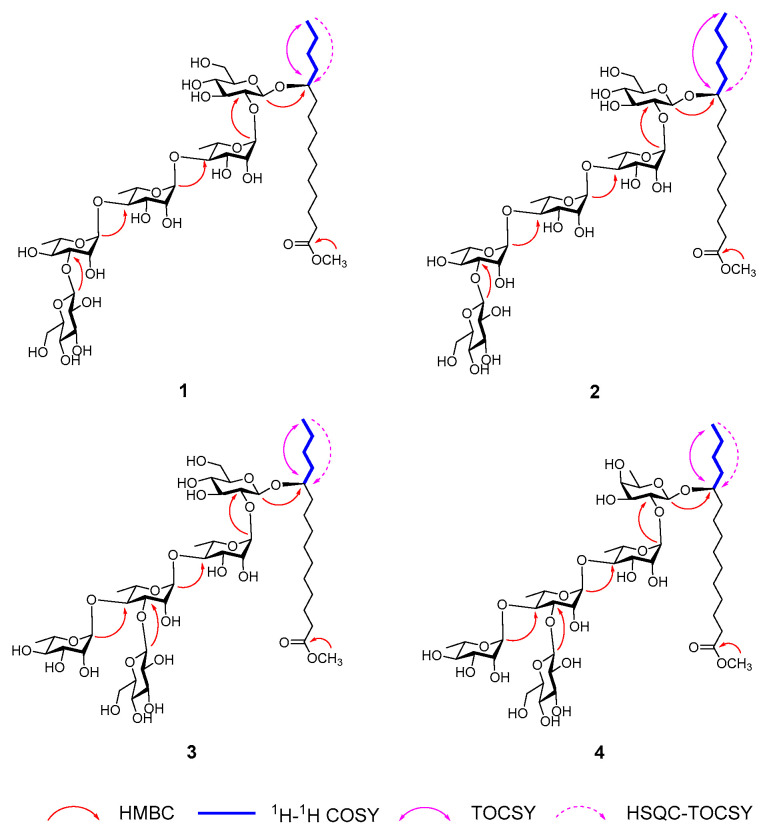
Key 2D NMR correlations of compounds **1**–**4**.

Detailed analysis of the 1D (^1^H and ^13^C) and 2D (HSQC, HMBC, TOCSY, ^1^H-^1^H COSY, HSQC-TOCSY) NMR spectra enabled the full assignment of the pentasaccharide framework, comprising two glucopyranosyl (Glc) and three rhamnopyranosyl (Rha) units (Figure 1). The absolute configurations of the monosaccharide units were determined by acid hydrolysis of the resin glycoside fraction, followed by HPLC analysis of their derivatives, which confirmed the presence of D-glucose and L-rhamnose [26,28]. The β-anomeric configurations of the two D-glucopyranosyl residues were supported by the large coupling constants (J = 7.2 and 7.8 Hz) observed at δ_H_ 4.97 and 5.38, respectively [34,35]. Meanwhile, the three L-rhamnopyranosyl units were assigned α-configurations, as indicated by characteristic C-5 chemical shifts at δ_C_ 67.7, 68.8, and 70.5 in the ^13^C NMR spectrum [36]. HMBC correlations further clarified the sugar sequence, showing cross-peaks from δ_H_ 6.41 (H-1, Rha) to δ_C_ 77.3 (C-2, Glc), δ_H_ 6.25 (H-1, Rha’) to δ_C_ 81.3 (C-4, Rha), δ_H_ 6.30 (H-1, Rha”) to δ_C_ 80.2 (C-4, Rha’), and δ_H_ 5.38 (H-1, Glc’) to δ_C_ 84.8 (C-3, Rha”) (Figure 2). Based on these data, the pentasaccharide moiety of compound **1** was defined as β-D-glucopyranosyl-(1→3)-O-α-L-rhamnopyranosyl-(1→4)-O-α-L-rhamnopyranosyl-(1→4)-O-α-L-rhamnopyranosyl-(1→2)-O-β-D-glucopyranoside.

Following the assignment of the pentasaccharide chain, the molecular formula of compound **1** suggested the presence of a monohydroxypentadecanoyl methyl ester as the aglycone. The ^1^H−^1^H COSY correlations traced from H-15 (δ_H_ 1.04) to H-14(δ_H_ 1.42), then to H-13(δ_H_ 1.62, 1.54), H-12(δ_H_ 1.74, 1.69), and finally to H-11 (δ_H_ 4.02), clearly indicated that the hydroxyl substituent was positioned at C-11 on the pentadecanoyl chain (Figure 2). This assignment was further supported by TOCSY correlations spanning from H-15 through H-14, H-13, H-12, to H-11, along with HSQC-TOCSY correlations connecting H-15 (δ_H_ 1.04) to carbons C-14 (δ_C_ 23.9), C-13 (δ_C_ 28.0), C-12 (δ_C_ 35.7), and C-11 (δ_C_ 78.5) (Figure 2). Consequently, the fatty acyl moiety was characterized as an 11-hydroxypentadecanoyl residue. The absolute S-configuration of this hydroxy fatty acid was inferred from specific rotation value ([α]^25^_D_ +2.0) of its phenacyl ester derivative, 2-oxo-2-phenylethyl 11S-hydroxypentadecanoate (**9**), obtained via phenacyl esterification of the hydrolysis product of the resin glycoside fraction. This matched well with literature values reported for methyl 11S-hydroxypentadecanoate [29].

The glycosidic linkage between the 11-hydroxypentadecanoyl aglycone and the pentasaccharide moiety was established by HMBC correlations from the anomeric proton H-1 (δ_H_ 4.97) of Glc to the carbon C-11 (δ_C_ 78.5) of the hydroxy fatty acid. Additionally, the presence of a methyl ester was confirmed by the HMBC cross-peak between the methoxy proton signal (δ_H_ 3.64) and the ester carbonyl carbon (δ_C_ 174.5) of the aglycone (Figure 2). Based on these collective data, the structure of compound 1 was conclusively determined as illustrated.

Compound **2**, also isolated as a colorless gum, exhibited a molecular formula of C_47_H_84_O_25_, evidenced by the HRESIMS ion peak at *m*/*z* 1071.5189 [M + Na]^+^, representing a 14-Da mass increase relative to compound **1**. The NMR spectra of compound **2** closely resembled those of compound **1**, with differences localized primarily in the high-field region (20–45 ppm), corresponding to the methylene signals of the fatty acyl chain (Table 1). These findings indicated that compound **2** was a homolog of compound **1**, featuring a hexadecanoyl rather than a pentadecanoyl aglycone. The hydroxyl group on the hexadecanoyl residue was similarly assigned at C-11, supported by ^1^H−^1^H COSY correlations from H-16 (δ_H_ 0.99) to H-15 (δ_H_ 1.42), H-14(δ_H_ 1.37), H-13(δ_H_ 1.64, 1.57), H-12(δ_H_ 1.76, 1.70), and H-11 (δ_H_ 4.02). This was corroborated by TOCSY correlations spanning H-16 through H-11, as well as HSQC-TOCSY correlations from H-16 (δ_H_ 0.99) to C-15 (δ_C_ 23.6), C-14 (δ_C_ 33.0), C-13 (δ_C_ 25.7), C-12 (δ_C_ 36.0), and C-11 (δ_C_ 78.6) (Figure 2). The S-configuration of the 11-hydroxyhexadecanoyl moiety was deduced from the specific rotation value ([α]^25^_D_ +2.0) of its phenacyl ester derivative, 2-oxo-2-phenylethyl 11S-hydroxyhexadecanoate (**11**), produced by hydrolysis followed by phenacyl esterification of the resin glycoside fraction [28]. The detailed structure of compound **2** was thus elucidated as depicted.

Cusponins III (**3**) and IV (**4**) were isolated as colorless gums. Their molecular formulas were determined as C_46_H_82_O_25_ and C_46_H_82_O_24_, respectively, based on HRESIMS data showing [M + Na]^+^ ions at m/z 1057.5035 for **3** and 1041.5096 for **4**. Detailed interpretation of their 1D and 2D NMR spectra (Table 1) revealed that both compounds shared a pentasaccharide core glycosidic acid methyl ester featuring an 11-hydroxypentadecanoyl aglycone. Notably, the sugar sequence of compound **3** resembled that of compounds **1** and **2**, with the exception that the Glc’ unit was linked at the C-3 position of Rha’ residue rather than the Rha” moiety, as evidenced by the HMBC correlation from δ_H_ 5.26 (H-1, Glc’) to δ_C_ 83.2 (C-3, Rha’) (Figure 2).

Comparatively, compounds **4** possessed a similar sugar composition to compound **3**, differing primarily by the substitution of the initial glucopyranosyl unit with a fucopyranosyl (Fuc) residue. This substitution was supported by HMBC cross-peaks linking H-1 (δ_H_ 4.80) of Fuc to C-11 (δ_C_ 78.3) of 11S-hydroxypentadecanoyl residue, alongside a correlation from δ_H_ 6.26 (H-1, Rha) to δ_C_ 75.6 (C-2, Fuc) (Figure 2). These data collectively permitted full structural assignment of cusponins III (**3**) and IV (**4**), which are the analogs of operculinic acid B methyl ester (**8**) and operculinic acid A methyl ester, respectively, with a pentadecanoyl instead of a hexadecanoyl aglycone [37].

Cusponin V (**5**) was also isolated as a colorless gum, with a molecular formula of C_50_H_88_O_25_ derived from HRESIMS [M + Na]^+^ ion at *m*/*z* 1111.5515 (calcd 1111.5512). Analysis of the 1D and 2D NMR data (Table 2) indicated that compound **5** contained four sugar residues: one Fuc, two Glc, and one Rha, in addition to the 11-hydroxypentadecanoyl aglycone and a methoxyl group, consistent with a tetrasaccharide glycosidic acid methyl ester framework. Intriguingly, two distinct sets of resonances corresponding to 3-hydroxy-2-methylbutanoyl (Nla) moieties were observed. The ^1^H NMR signals appeared at δ_H_ 3.01 (dq, J = 7.2, 7.2 Hz), 5.59 (m), 1.36 (d, J = 6.6 Hz), and 1.28 (d, J = 7.2 Hz) for one group, and at δ_H_ 2.81 (dq, J = 7.2, 7.2 Hz), 4.33 (m), 1.36 (d, J = 6.6 Hz), and 1.28(d, J = 7.2 Hz) for the other. Corresponding ^13^C resonances were assigned at δ_C_ 174.1, 45.7, 71.8, 17.2, 13.1 and δ_C_ 175.3, 48.9, 69.6, 21.5, 13.8, respectively. The absolute configurations of these Nla residues were determined as 2R,3R based on the specific rotation ([α]^25^_D_ −12.0) of their derivative, 4-bromophenyacyl-(2R,3R)-nilate (**11**, Figure 1), which matched authentic standards [26].

**Figure 3:**
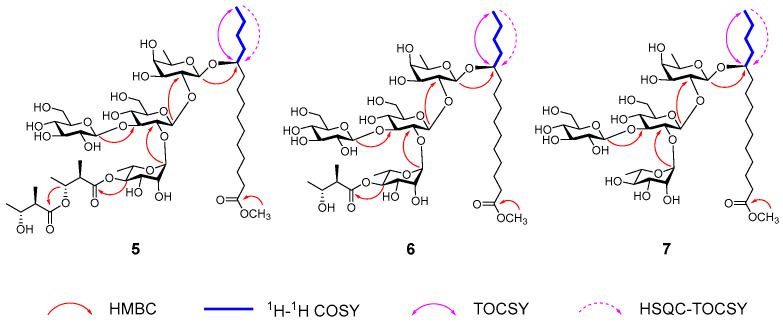
Key 2D NMR correlations of compounds **5**–**7**.

The glycosidic linkages in compound **5** were elucidated through key HMBC correlations: from the anomeric proton δ_H_ 4.98 (H-1, Glc’) to δ_C_ 90.3 (C-3, Glc), from δ_H_ 6.47 (H-1, Rha) to δ_C_ 77.5 (C-2, Glc), from δ_H_ 5.72 (H-1, Glc) to δ_C_ 78.1 (C-2, Fuc), and from δ_H_ 4.82 (H-1, Fuc) to δ_C_ 81.2 (C-11, 11-hydroxypentadecanoyl moiety) (Figure 3). The attachment of the two Nla moieties was confirmed by the HMBC cross peaks between δ_H_ 5.89 (H-4, Rha) and δ_C_ 174.1 (C-1, Nla), and between δ_H_ 5.59 (H-3, Nla) tand δ_C_ 175.3 (C-1, Nla’) (Figure 2). Furthermore, the methyl ester linkage was substantiated by the correlation from methyl proton at δ_H_ 3.63 to the carbonyl carbon C-1 (δ_C_ 174.5) of the 11-hydroxypentadecanoyl residue (Figure 3). Collectively, these data enabled the full structural assignment of compound **5** as depicted.

Cusponins VI (**6**) and VII (**7**), both isolated as colorless gums, exhibited HRESIMS ion peaks at m/z 1011.4988 [M + Na]^+^ (C_45_H_80_O_23_Na) and 911.4465 [M + Na]^+^ (C_40_H_72_O_21_Na), respectively. Comprehensive NMR analysis revealed that both compounds shared the identical tetrasaccharide core, 11-hydroxypentadecanoyl aglycone, and methyl ester features found in compound **5** (Table 2). Distinguishingly, compound **7** lacked any acyl modification on the sugar chain. In contrast, in compound **6**, the C-4 position of the Rha residue was esterified with an Nla group, as supported by the HMBC correlation from δ_H_ 5.94 (H-4, Rha) to δ_C_ 175.8 (C-1, Nla) (Figure 3). These observations led to the structural elucidation of compounds **6** and **7** as illustrated.

Compound **8** was identified as the known natural product operculinic acid B methyl ester, based on comprehensive NMR (^1^H, ^13^C, HSQC, HMBC, TOCSY, ^1^H-^1^H COSY, HSQC-TOCSY) and HRESIMS data [37]. Notably, its NMR data differed somewhat from previously reported values and are detailed in Table S1 of the Supporting Information.

### 3.2. Enzyme Inhibitory Activity

Given the increasing number of studies highlighting the α-glucosidase inhibitory potential of resin glycosides [16,17,18,19], the inhibitory effects of the isolated compounds **1**–**8** against α-glucosidase were assessed. As summarized in Table 3, all compounds demonstrated varying degrees of inhibition, ranging from 49.41% to 83.00% at a concentration of 100 μM. Subsequent detailed assays revealed that compounds **1**–**4**, **6** and **8** effectively suppressed α-glucosidase activity, with IC_50_ values spanning from 8.02 to 71.39 μM. Among these, compound **2** exhibited the strongest inhibition with an IC_50_ of 8.02 ± 2.90 μM.

To further explore their antidiabetic potential, the inhibitory activity against PTP1B was evaluated. Notably, compounds **3** and **5** showed significant PTP1B inhibition, with IC_50_ values of 14.19 ± 1.29 μM and 62.31 ± 8.61 μM, respectively (Table 3). This study represented the first report of PTP1B inhibitory activity associated with resin glycosides. Furthermore, compound **3** emerged as a promising dual-target inhibitor, effectively acting against both α-glucosidase and PTP1B.

### 3.3. Reversibility and Inhibitory Kinetics

To further characterize the enzyme inhibition properties of resin glycosides, the reversibility and kinetic behavior of the most potent inhibitors, compound **2** for *α*-glucosidase and compound **3** for PTP1B were examined.

**Figure 4:**
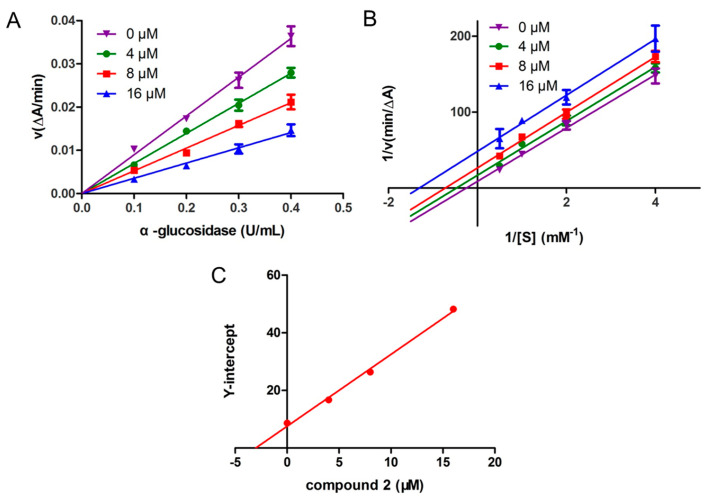
The reversibility and inhibitory kinetics of α-glucosidase inhibition by compound **2**. (**A**) The catalytic activity of α-glucosidase enzyme at different concentrations of compound **2**. (**B**) Lineweaver–Burk plot analysis of the kinetics of α-glucosidase inhibition by compound **2**. (**C**) The secondary plots of Y-intercept versus inhibitor concentration used to determine the inhibition constant *K*_is_. Data used in plots taken from the Lineweaver–Burk plots.

The reversibility of α-glucosidase inhibition by compound **2** was assessed by plotting reaction velocity against enzyme concentration at different inhibitor levels (Figure 4A). The resulting straight lines all passed through the origin, with slopes decreasing as compound **2** concentration increased, indicating that inhibition was reversible and involved non-covalent interactions [38].

The inhibition mechanism of compound **2** was further analyzed using Lineweaver–Burk and secondary plots. The Lineweaver–Burk plots (Figure 4B) displayed a series of parallel lines, characteristic of uncompetitive inhibition [39]. Increasing the concentration of compound **2** from 0 to 16 μM led to a reduction in *K*_m_ value from 4.04 to 0.77 and in *V*_max_ value from 0.115 to 0.021. This behavior confirmed that compound **2** bound exclusively to the enzyme–substrate complex, forming an enzyme–substrate-inhibitor ternary complex. The inhibition constant *K*_is_, reflecting the affinity of compound **2** for the enzyme–substrate complex, was calculated as 3.02 μM, based on secondary plots of the Y-intercept derived from the Lineweaver–Burk data (Figure 4C).

**Figure 5:**
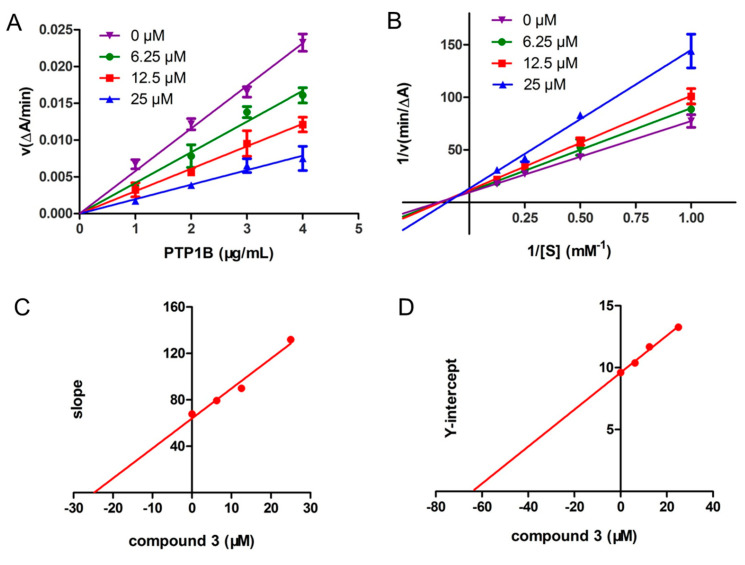
The reversibility and inhibitory kinetics of PTP1B inhibition by compound **3**. (**A**) The catalytic activity of PTP1B enzyme at different concentrations of compound **3**. (**B**) Lineweaver–Burk plot analysis of the kinetics of PTP1B inhibition by compound **3**. (**C**) The secondary plots of slope versus inhibitor concentration used to determine the inhibition constant *K*_i_. (**D**) The secondary plots of Y-intercept versus inhibitor concentration used to determine the inhibition constant *K*_is_. Data used in plots taken from the Lineweaver–Burk plots.

Using a similar approach, the reversibility of PTP1B inhibition by compound **3** was investigated. As depicted in Figure 5A, all lines passed through the origin, indicating that compound **3** was also a reversible inhibitor of PTP1B. The Lineweaver–Burk plot (Figure 5B) revealed that the lines intersected in the second quadrant, consistent with mixed-type inhibition [40]. Moreover, with increasing concentrations of compound **3** increased from 0 to 25 μM, the *K*_m_ values rose from 7.05 to 9.94, while the *V*_max_ values declined from 0.104 to 0.075, further confirming the mixed-type inhibitory behavior.

As a mixed-type inhibitor, compound **3** could bind both the free enzyme and the enzyme–substrate complex. Accordingly, two inhibition constants, *K*_i_ and *K*_is_, were determined, reflecting the binding affinities for the free enzyme and the enzyme–substrate complex, respectively. Based on secondary plots derived from the Lineweaver–Burk data (Figure 5C,D), *K*_i_ and *K*_is_, were calculated to be 24.82 μM and 64.24 μM, respectively. The substantially lower *K*_i_ compared to *K*_is_ suggested that compound **3** preferentially bound more tightly and readily to the free PTP1B enzyme than to the enzyme–substrate complex.

### 3.4. Molecular Docking Analysis

The molecular docking studies were conducted to elucidate the binding interactions of compounds **2** and **3** with *α*-glucosidase and PTP1B, respectively.

**Figure 6:**
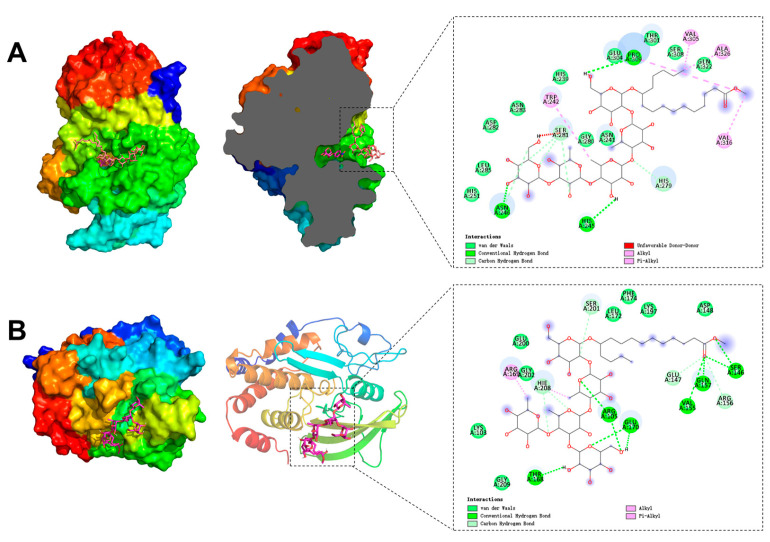
Three-dimensional and 2D ligand interactions diagrams of compound 2 with α-glucosidase-pNPG complex (**A**) and compound 3 with PTP1B (**B**).

Since compound **2** acted as an uncompetitive inhibitor binding to the enzyme–substrate complex, it was docked with the *α*-glucosidase-pNPG complex. The *α*-glucosidase homology model was generated via the I-TASSER online platform [19,31]. As depicted in Figure 6A, pNPG occupied the enzyme’s active site, while compound **2** situated itself effectively at the pocket entrance, thereby obstructing substrate access. The 2D ligand interaction analysis revealed that compound **2** formed hydrogen bonds with His245, Asn246, and Pro309, alongside alkyl and π-alkyl contacts involving Trp242, Val305, Val316, and Ala326. Furthermore, numerous van der Waals interactions with residues, such as His251, Leu285, Asp282, Asn283, His239, Glu304, Thr301, Ser308, and Gln322, were observed. The calculated binding affinity was −7.1 kcal/mol, indicating a robust interaction between compound **2** and the *α*-glucosidase-pNPG complex.

Molecular docking of compound **3** with PTP1B (PDB ID: 2QBQ) was performed, considering its propensity to bind the free enzyme. As illustrated in Figure 6B, compound **3** was securely docked near the allosteric site of PTP1B, exhibiting a binding energy of −6.3 kcal/mol [30,41]. The 2D interaction analysis revealed that compound **3** formed hydrogen bonds with key residues Thr168, Arg105, Glu170, Ser146, Gln 157, and Val155. Additionally, alkyl and π-alkyl interactions were observed with Arg169 and Hie208, while van der Waals contacts involved multiple amino acids, including Gly209, Lys103, Gly202, Glu200, Leu172, Phe174, Lys197, and Asp148.

### 3.5. MD Simulation

To further elucidate the interactions of compounds **2** and **3** with the *α*-glucosidase-pNPG complex and PTP1B enzyme, respectively, MD simulations were performed using the docking complexes described above [33].

**Figure 7:**
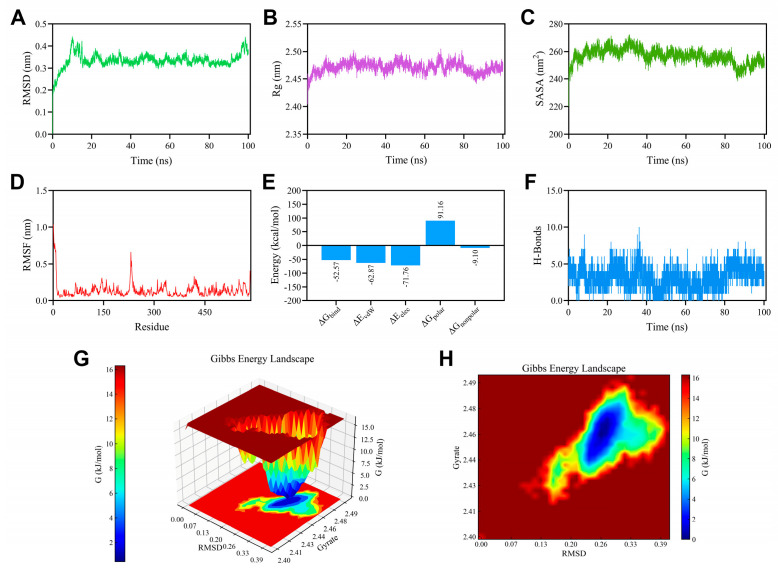
Molecular dynamics simulation of α-glucosidase-pNPG-**2** complex. (**A**) RMSD profile. (**B**) Rg profile. (**C**) SASA profile. (**D**) RMSF profile. (**E**) Contributions to the binding free energy (kcal/mol). (**F**) Number of H-bonds formed between compound **2** and α-glucosidase during MD simulation. (**G**,**H**) Three and two dimensional gibbs FEL plot contour from MD simulation trajectories.

During MD simulations, the root mean square deviation (RMSD) of the system relative to the initial structure served as a critical parameter to assess stability. The RMSD trajectory of the *α*-glucosidase-pNPG-**2** complex indicated that the system stabilized after roughly 20 ns, confirming equilibration (Figure 7A). The radius of gyration (Rg), which reflects the overall compactness and folding state of the protein, fluctuated narrowly between 2.45 and 2.50 nm, suggesting that the protein structure remained consistently stable throughout the simulation period (Figure 7B). The solvent-accessible surface area (SASA), measuring the extent of residue exposure to solvent, initially increased but then plateaued around 250–270 nm^2^. This trend indicated conformational rearrangements leading to increased solvent exposure of the complex surface (Figure 7C).

The root mean square fluctuation (RMSF) profile revealed minimal positional variation for most amino acid residues of *α*-glucosidase, further demonstrating structural stability during the entire simulation (Figure 7D). The binding free energy, calculated via the MM-PBSA approach, was determined to be −52.57 kcal/mol, supporting a strong and energetically favorable interaction (Figure 7E). Energy component analysis showed that van der Waals forces, electrostatic interactions, and nonpolar solvation energies all contributed positively to the binding affinity. In contrast, polar solvation energy had an unfavorable influence. Particularly, electrostatic interactions played a dominant role relative to van der Waals and nonpolar solvation contributions. Hydrogen bonds, which represent a key electrostatic interaction, were abundant: compound **2** formed between 3 and 10 hydrogen bonds with various residues of *α*-glucosidase, underscoring the stability of the complex (Figure 7F).

The Gibbs free energy landscape (FEL) was generated to visualize the energy minima and conformational stability of the complex (Figure 7G,H). The compactness and density of the minimum energy basin (depicted in blue) within the FEL plot were indicative of a highly stable binding interaction. For the *α*-glucosidase-pNPG-2 system, the FEL showed a well-defined and concentrated energy minimum, confirming that the complex was structurally stable and energetically favorable.

**Figure 8:**
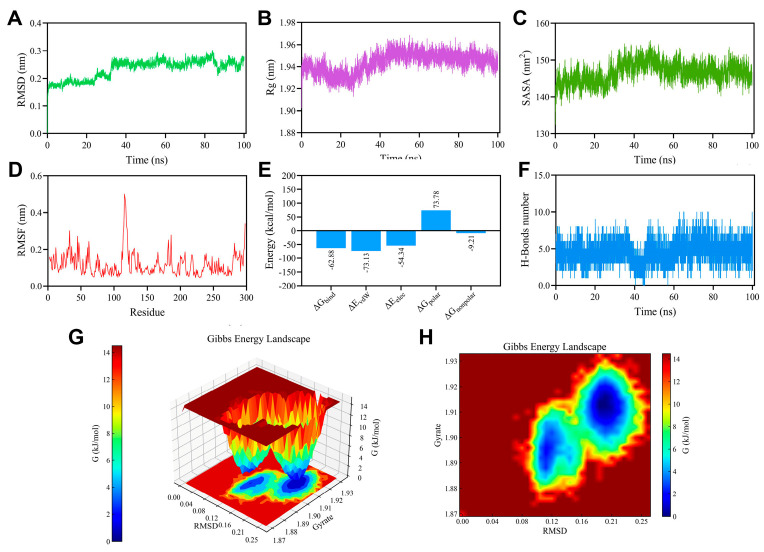
Molecular dynamics simulation of PTP1B-**3** complex. (**A**) RMSD profile. (**B**) Rg profile. (**C**) SASA profile. (**D**) RMSF profile. (**E**) Contributions to the binding free energy (kcal/mol). (**F**) Number of H-bonds formed between compound **3** and PTP1B during MD simulation. (**G**,**H**) Three and two dimensional FEL plot contour from MD simulation trajectories.

For the PTP1B-compound **3** complex, the RMSD values rose from near zero to approximately 0.25 nm over the simulation timeframe, indicating that the complex experienced gradual conformational adjustments during the MD process (Figure 8A). The Rg fluctuated modestly between 1.91 and 1.97 nm, demonstrating that the overall structural compactness of the complex remained consistently stable throughout the simulation (Figure 8B). The SASA was maintained within the range of 140–155 nm^2^, reflecting sustained solvent exposure and stable solvent-protein interactions (Figure 8C). The RMSF analysis offered further evidence of structural stability, showing no significant residue-level fluctuations in PTP1B during the entire simulation period (Figure 8D).

The binding free energy computed via MM-PBSA was −62.88 kcal/mol, suggesting a robust and energetically favorable interaction between PTP1B and compound **3**, with van der Waals forces contributing the most substantially to the binding affinity (Figure 8E). Moreover, hydrogen bonding analysis revealed that compound **3** consistently formed approximately five hydrogen bonds with key residues of PTP1B throughout the simulation (Figure 8F). The Gibbs FEL plots (Figure 8G,H) exhibited densely concentrated blue regions, indicative of the highly stable and energetically preferred binding interactions between compound **3** and PTP1B.

## 4. Conclusions

In this study, a comprehensive chemical investigation of resin glycosides extracted from *C. japonica* seeds resulted in the isolation of eight resin glycosides, among which seven were novel and designated as cusponins I-VII (**1**–**7**). All isolated compounds were identified as glycosidic acid methyl esters, likely formed via methyl esterification during NH_2_ silica gel chromatography. Structural analysis revealed that compounds **1**–**4** were pentasaccharides, whereas compounds **5**–**7** consisted of tetrasaccharide frameworks. Notably, compounds **1** and **3**–**7** contained the uncommon 11*S*-hydroxypentadecanoic acid as their aglycone moiety.

Biological activity assays demonstrated that compounds **1**–**4**, **6**, and **8** effectively inhibited *α*-glucosidase, with IC_50_ values ranging from 8.02 to 71.39 μM. Meanwhile, compounds **3** and **5** showed significant inhibition against PTP1B, with IC_50_ values of 14.19 ± 1.29 μM and 62.31 ± 8.61 μM, respectively, marking the first report of PTP1B inhibitory activity among resin glycosides. Enzymatic kinetic studies indicated that compound **2** and **3** are uncompetitive α-glucosidase inhibitor and mixed-type PTP1B inhibitor, respectively. Molecular docking and MD simulations further confirmed that compounds **2** and **3** stably interacted with *α*-glucosidase-pNPG and PTP1B, respectively, forming complexes characterized by favorable conformational stability and strong binding free energies.

Together, these findings advanced the understanding on the structural diversity of resin glycosides, and provided valuable insights for the development of novel lead compounds targeting diabetes treatment, although more research on their bioavailability and absorption are still needed.

## Figures and Tables

**Table 1:** ^1^H and ^13^C NMR spectroscopic data of compounds **1**–**4** (600 and 151 MHz, respectively, in pyridine-d5) ^a^.

Position ^b^	1		2		3		4	
	*δ*_H_	*δ*_C_	*δ*_H_	*δ*_C_	*δ*_H_	*δ*_C_	*δ*_H_	*δ*_C_
Glc-1	4.97 (7.2)	101.5	4.98 d (7.2)	101.6	5.01 d (7.2)	101.3	5.26 d (7.8)	106.0
2	4.26	77.3	4.26	77.4	4.26	77.6	4.00 br t (8.4)	75.6
3	4.27	80.2	4.28	80.2	4.29 t (9.6)	80.1	4.21 dd (9.0, 9.0)	78.9
4	4.18 dd (9.0, 9.0)	72.5	4.17 dd (9.0, 9.0)	72.5	4.19	72.5	4.14	72.1
5	3.90	78.7	3.90 m	78.6	3.92 m	78.7	3.97	79.0
6	4.37	63.2	4.37	63.3	4.37	63.3	4.31	63.4
	4.53		4.52 dd (2.4, 12.0)		4.52		4.56	
Fuc-1							4.80 d (7.8)	101.6
2							4.52	75.6
3							4.16	77.1
4							4.95 d (3.0)	74.0
5							3.81 m	71.7
6							1.53 d (6.6)	17.7
Rha-1	6.41 br s	101.8	6.40 br s	101.9	6.38 br s	102.0	6.26 br s	102.0
2	4.66 br s	73.2	4.68 br s	73.2	4.69 br s	73.1	4.69 br s	73.2
3	4.63	73.8	4.64 dd (3.0, 9.0)	73.8	4.63	73.1	4.63 dd (3.6, 9.0)	73.2
4	4.38	81.3	4.39	81.3	4.29 t (9.0)	82.7	4.25	82.7
5	4.92 m	67.7	4.91 m	67.8	4.93	68.2	4.68	68.0
6	1.67 d (6.6)	19.6	1.68 d (6.6)	19.6	1.72 d (6.6)	19.3	1.61 d (6.6)	19.2
Rha*’*-1	6.25 br s	103.5	6.25 br s	103.5	5.92 d (1.8)	104.0	5.91 br s	103.9
2	4.79 br s	73.6	4.80 br s	73.6	5.21 br s	72.5	5.20 br s	72.5
3	4.58 dd (3.0, 9.0)	73.7	4.59 dd (3.0, 9.0)	73.8	4.76 dd (3.0, 9.0)	83.2	4.75 dd (3.0, 9.0)	83.2
4	4.46 t (9.0)	80.2	4.47	80.2	4.53 t (9.0)	79.0	4.52	79.1
5	4.41	68.8	4.41	68.8	4.44 m	69.0	4.41 m	69.0
6	1.62 d (6.0)	19.3	1.62 d (6.0)	19.3	1.62 d (6.0)	19.4	1.61 d (6.6)	19.4
Rha”-1	6.30 br s	103.7	6.30 br s	103.7	6.25 br s	103.6	6.24 br s	103.6
2	5.07 br s	72.3	5.07 br s	72.2	4.90 br s	73.1	4.91 br s	73.2
3	4.61 dd (3.0, 9.0)	84.8	4.61 dd (3.0, 9.6)	84.8	4.45	73.2	4.45 dd (3.6, 9.0)	73.2
4	4.50	73.4	4.49	73.4	4.26	74.4	4.25	74.4
5	4.42	70.5	4.42	70.5	4.33 m	70.9	4.33	70.9
6	1.57 d (6.6)	18.8	1.57 d (6.0)	18.8	1.60 d (6.0)	18.9	1.59 d (7.2)	18.9
Glc*’*-1	5.38 d (7.8)	107.3	5.38 d (7.8)	107.2	5.26 d (7.8)	106.0		
2	4.08 br t (8.4)	76.5	4.08 br t (8.4)	76.5	3.99	75.6		
3	4.24 t (9.0)	78.8	4.24 t (9.0)	78.8	4.20	78.9		
4	4.36	71.5	4.36 t (9.0)	71.5	4.14	72.1		
5	3.83 m	78.8	3.84 m	78.8	3.97 m	79.1		
6	4.41	62.6	4.41	62.6	4.30	63.4		
	4.43		4.43		4.56			
Ag-1		174.5		174.5		174.5		174.5
2	2.32 t (7.2)	34.6	2.32 t (7.2)	34.6	2.33 t (7.2)	34.6	2.34 t (7.2)	34.6
11	4.02 m	78.5	4.02 m	78.6	4.07 m	78.3	4.00	78.3
12	1.74	35.7	1.76	36.0	1.77	35.6	1.77	35.8
	1.69		1.70					
13	1.62	28.0	1.57	25.7	1.64	28.1	1.63	28.2
	1.54		1.64		1.53		1.55	
14	1.42	23.9	1.37	33.0	1.37	23.8	1.40	23.7
15	1.04 t (7.2)	15.0	1.42	23.6	0.95 t (7.2)	14.9	0.96 t (7.2)	14.9
16			0.99 t (7.2)	15.0				
-OCH3	3.64 s	51.7	3.64 s	51.7	3.62 s	51.7	3.62 s	51.7

^a^ Overlapped signals are reported without designating multiplicity; ^b^ Abbreviations: Glc = glucopyranosyl, Rha = rhamnopyranosyl, Fuc = fucopyranosyl, Ag = 11-hydroxypentadecanoyl for **1**,**3**, and **4**, and 11-hydroxyhexadecanoyl for **2**.

**Table 2:** ^1^H and ^13^C NMR spectroscopic data of compounds **5**–**7** (600 and 151 MHz, respectively, in pyridine-d5) ^a^.

Position ^b^	5		6		7	
	*δ*_H_	*δ*_C_	*δ*_H_	*δ*_C_	*δ*_H_	*δ*_C_
Fuc-1	4.82 d (7.2)	103.2	4.81 d (7.2)	103.2	4.82 d (7.2)	103.1
2	4.64 dd (7.2, 9.0)	78.1	4.62 dd (7.2, 9.0)	78.2	4.60 br t (8.4)	78.1
3	4.48 dd (3.0, 9.0)	77.0	4.53 dd (3.0, 9.0)	76.9	4.44 dd (3.0, 9.6)	76.8
4	4.22 br s	73.6	4.11	73.7	3.84 d (3.0)	73.4
5	4.06	71.8	4.06	71.5	3.77 br q (6.0)	71.4
6	1.67 d (6.0)	17.9	1.61 d (6.6)	17.9	1.50 d (6.0)	17.8
Glc-1	5.72 d (7.8)	102.3	5.75 d (7.8)	102.3	5.75 d (7.8)	102.4
2	4.24	77.5	4.25 br t (8.4)	77.2	4.28 br t (8.4)	77.7
3	4.07	90.3	4.07	90.4	4.08	90.1
4	4.03	70.5	4.03	70.5	4.06	70.5
5	3.57 m	77.5	3.58 m	77.5	3.59 m	77.5
6	4.20	63.1	4.20 dd (1.8, 11.4)	63.1	4.20 br d (12.0)	63.1
	4.12		4.12		4.12 m	
Rha-1	6.47 br s	102.1	6.51 br s	102.0	6.44 br s	102.4
2	4.85 br s	72.8	4.88 br s	72.6	4.89 br s	72.9
3	4.84	70.7	4.92 dd (3.0, 9.6)	70.9	4.77 dd (3.0, 9.6)	73.2
4	5.89 t (9.6)	77.0	5.94 t (9.6)	76.7	4.36 t (9.6)	74.8
5	5.24 m	67.3	5.29 m	67.5	5.08 m	70.1
6	1.68 d (6.6)	19.0	1.74 d (6.6)	19.0	1.88 d (6.0)	19.6
Glc’-1	4.98	105.3	4.98	105.3	5.02 d (7.8)	105.0
2	4.03	75.2	4.03	75.2	4.03	75.4
3	4.16 t (9.0)	79.2	4.16 t (9.0)	79.2	4.17	79.1
4	4.12	71.9	4.12	71.9	4.12	71.9
5	4.04	79.1	4.02 m	79.1	4.02	79.1
6	4.57 dd (1.8, 11.4)	62.8	4.57 br d (10.8)	62.8	4.56 br d (11.4)	62.9
	4.27		4.27		4.26	
Ag-1		174.5		174.5		174.5
2	2.33 t (7.8)	34.6	2.33 t (7.2)	34.6	2.33 t (7.8)	34.6
11	3.93 m	81.2	3.88 m	81.2	3.92 m	81.1
12	1.83	35.5	1.80	35.5	1.82	35.5
	1.68		1.66		1.66	
13	1.52	28.2	1.50	28.2	1.52	28.2
14	1.30	23.6	1.31	23.6	1.31	23.6
15	0.89 t (7.2)	14.8	0.88 t (7.2)	14.8	0.88 t (7.2)	14.8
-OCH3	3.63 s	51.7	3.63 s	51.7	3.63 s	51.7
Nla-1		174.1		175.8		
2	3.01 dq (7.2, 7.2)	45.7	2.80 dq (6.6, 6.6)	49.9		
3	5.59 m	71.8	4.32 dq (6.6, 6.6)	70.1		
4	1.36 d (6.6)	17.2	1.34 d (6.6)	21.6		
5	1.28 d (7.2)	13.1	1.28 d (6.6)	14.3		
Nla’-1		175.3				
2	2.81 dq (7.2, 7.2)	48.9				
3	4.33 m	69.6				
4	1.36 d (6.6)	21.5				
5	1.28 d (7.2)	13.8				

^a^ Overlapped signals are reported without designating multiplicity; ^b^ Abbreviations: Glc = glucopyranosyl, Rha = rhamnopyranosyl, Fuc = fucopyranosyl, Qui = quinovopyranosyl, Ag = 12-hydroxyhexadecanoyl aglycone, Nla = 3-hydroxy-2-methylbutanoyl.

**Table 3:** α-glucosidase and PTP1B inhibitory activities of the isolated compounds.

Compound	α-Glucosidase		PTP1B	
	Inhibition ^a^ (%)	IC_50_^b^ (μM)	Inhibition ^a^ (%)	IC_50_^b^ (μM)
1	68.72 ± 4.11	38.21 ± 3.36	5.41 ± 0.67	>100
2	83.00 ± 0.84	8.02 ± 2.90	3.26 ± 0.16	>100
3	67.13 ± 1.18	64.92 ± 2.97	89.23 ± 2.55	14.19 ± 1.29
4	66.55 ± 3.65	55.53 ± 6.89	15.02 ± 2.45	>100
5	51.78 ± 1.77	>100	52.74 ± 4.60	62.31 ± 8.61
6	60.51 ± 5.42	71.39 ± 3.23	29.85 ± 4.50	>100
7	49.41 ± 1.71	>100	15.93 ± 2.92	>100
8	64.60 ± 3.12	65.08 ± 9.41	7.78 ± 1.98	>100
Genistein	50.14 ± 1.17	99.87 ± 2.43	-	-
Ursolic acid	-	-	90.43 ± 5.23	16.41 ± 1.99

^a^ Percent inhibition at a concentration of 100 μM. ^b^ Inhibitor concentration (mean ± SD of three independent experiments) required for 50% inactivation of α-glucosidase or PTP1B.

## Data Availability

The original contributions presented in this study are included in the article/Supplementary Materials. Further inquiries can be directed to the corresponding authors.

## Supplementary Materials

The following supporting information can be downloaded at: https://www.mdpi.com/article/10.3390/biom15101465/s1. Table S1: ^1^H and ^13^C NMR spectroscopic data of compound **8** (600 and 151 MHz, respectively, in pyridine-d_5_)*^a^*; Figures S1: NMR and HRESIMS spectra of compounds **1**–**8**.
