# Supplementary material for: Resin Glycosides with α-Glucosidase and Protein Tyrosine Phosphatase 1B Inhibitory Activities from the Seeds of Cuscuta japonica
Source: Biomolecules. 2025 Oct 16;15(10):1465. doi: 10.3390/biom15101465 (PMC12562854; doi:10.3390/biom15101465)
Supplement: Supplementary file 1 [file biomolecules-15-01465-s001.zip › biomolecules-3884196-supplementary.pdf]

Supporting Information

Resin Glycosides with *α*-Glucosidase and Protein Tyrosine Phosphatase 1B Inhibitory Activities from the Seeds of *Cuscuta japonica*

Su-Peng Guo [1,†], Ye He [2,†], Xin Lan [1], Tian-Zi Qi [1], Jin-Ping Gu [1], Jia Guo [1,2], Xin-Yu Wang [1], Min Yang [1], Wen-Li Wang [1], Guang-Tong Chen [1,*], Bo-Yi Fan [1,*]

1 School of Pharmacy, Nantong University, 9 Seyuan Road, Nantong 226019, People's Republic of China;
2 Jinghua Pharmaceutical Group Co., Ltd., 9 Xingtai Road, Nantong 226005, People's Republic of China
* Correspondence: guangtong_chen@163.com (G.-T. Chen); fanboyi@ntu.edu.cn (B.-Y. Fan)
† These authors contributed equally to this work.

Table of contents

Table S1 ^{1}H and ^{13}C NMR spectroscopic data of compound **8** (600 and 151 MHz, respectively, in pyridine-*d*5)[a]

Figure S1. ^{1}H-NMR spectrum of compound **1** (600 MHz, pyridine-*d*5)

Figure S2. ^{13}C-NMR spectrum of compound **1** (151 MHz, pyridine-*d*5)

Figure S3. HSQC spectrum of compound **1** (600 MHz, pyridine-*d*5)

Figure S4. Expanded HSQC spectrum of compound **1** (600 MHz, pyridine-*d*5)

Figure S5. HMBC spectrum of compound **1** (600 MHz, pyridine-*d*5)

Figure S6. Expanded HMBC spectrum on the glycosidic linkages of compound **1** (600 MHz, pyridine-*d*5)

Figure S7. Expanded HMBC spectrum on the ester linkages of compound **1** (600 MHz, pyridine-*d*5)

Figure S8. ^{1}H-^{1}H COSY spectrum of compound **1** (600 MHz, pyridine-*d*5)

Figure S9. Expanded ^{1}H-^{1}H COSY spectrum of compound **1** (600 MHz, pyridine-*d*5)

Figure S10. TOCSY spectrum of compound **1** (600 MHz, pyridine-*d*5)

Figure S11. Expanded TOCSY spectrum of compound **1** (600 MHz, pyridine-*d*5)

Figure S12. HSQC-TOCSY spectrum of compound **1** (600 MHz, pyridine-*d*5)

Figure S13. Expanded HSQC-TOCSY spectrum of compound **1** (600 MHz, pyridine-*d*5)

Figure S14. HRESIMS spectrum of compound **1**

Figure S15. ^{1}H-NMR spectrum of compound **2** (600 MHz, pyridine-*d*5)

Figure S16. ^{13}C-NMR spectrum of compound **2** (151 MHz, pyridine-*d*5)

Figure S17. HSQC spectrum of compound **2** (600 MHz, pyridine-*d*5)

Figure S18. Expanded HSQC spectrum of compound **2** (600 MHz, pyridine-*d*5)

Figure S19. HMBC spectrum of compound **2** (600 MHz, pyridine-*d*5)

Figure S20. Expanded HMBC spectrum on the glycosidic linkages of compound **2** (600 MHz, pyridine-*d*5)

Figure S21. Expanded HMBC spectrum on the ester linkages of compound **2** (600 MHz, pyridine-*d*5)

Figure S22. ^{1}H-^{1}H COSY spectrum of compound **2** (600 MHz, pyridine-d_5)

Figure S23. Expanded ^{1}H-^{1}H COSY spectrum of compound **2** (600 MHz, pyridine-d_5)

Figure S24. TOCSY spectrum of compound **2** (600 MHz, pyridine-d_5)

Figure S15. Expanded TOCSY spectrum of compound **2** (600 MHz, pyridine-d_5)

Figure S26. HSQC-TOCSY spectrum of compound **2** (600 MHz, pyridine-d_5)

Figure S27. Expanded HSQC-TOCSY spectrum of compound **2** (600 MHz, pyridine-d_5)

Figure S28. HRESIMS spectrum of compound **2**

Figure S29. ^{1}H-NMR spectrum of compound **3** (600 MHz, pyridine-d_5)

Figure S30. ^{13}C-NMR spectrum of compound **3** (151 MHz, pyridine-d_5)

Figure S31. HSQC spectrum of compound **3** (600 MHz, pyridine-d_5)

Figure S32. Expanded HSQC spectrum of compound **3** (600 MHz, pyridine-d_5)

Figure S33. HMBC spectrum of compound **3** (600 MHz, pyridine-d_5)

Figure S34. Expanded HMBC spectrum on the glycosidic linkages of compound **3** (600 MHz, pyridine-d_5)

Figure S35. Expanded HMBC spectrum on the ester linkages of compound **3** (600 MHz, pyridine-d_5)

Figure S36. ^{1}H-^{1}H COSY spectrum of compound **3** (600 MHz, pyridine-d_5)

Figure S37. TOCSY spectrum of compound **3** (600 MHz, pyridine-d_5)

Figure S38. HSQC-TOCSY spectrum of compound **3** (600 MHz, pyridine-d_5)

Figure S39. HRESIMS spectrum of compound **3**

Figure S40. ^{1}H-NMR spectrum of compound **4** (600 MHz, pyridine-d_5)

Figure S41. ^{13}C-NMR spectrum of compound **4** (151 MHz, pyridine-d_5)

Figure S42. HSQC spectrum of compound **4** (600 MHz, pyridine-d_5)

Figure S43. Expanded HSQC spectrum of compound **4** (600 MHz, pyridine-d_5)

Figure S44. HMBC spectrum of compound **4** (600 MHz, pyridine-d_5)

Figure S45. Expanded HMBC spectrum on the glycosidic linkages of compound **4** (600 MHz, pyridine-d_5)

Figure S46. Expanded HMBC spectrum on the ester linkages of compound **4** (600 MHz, pyridine-d_5)

Figure S47. ^{1}H-^{1}H COSY spectrum of compound **4** (600 MHz, pyridine-d_5)

Figure S48. TOCSY spectrum of compound **4** (600 MHz, pyridine-d_5)

Figure S49. HSQC-TOCSY spectrum of compound **4** (600 MHz, pyridine-d_5)

Figure S50. HRESIMS spectrum of compound **4**

Figure S51. ^{1}H-NMR spectrum of compound **5** (600 MHz, pyridine-d_5)

Figure S52. ^{13}C-NMR spectrum of compound **5** (151 MHz, pyridine-d_5)

Figure S53. HSQC spectrum of compound **5** (600 MHz, pyridine-d_5)

Figure S54. Expanded HSQC spectrum of compound **5** (600 MHz, pyridine-d_5)

Figure S55. HMBC spectrum of compound **5** (600 MHz, pyridine-d_5)

Figure S56. Expanded HMBC spectrum on the glycosidic linkages of compound **5** (600 MHz, pyridine-d_5)

Figure S57. Expanded HMBC spectrum on the ester linkages of compound **5** (600 MHz, pyridine-d_5)

Figure S58. ^{1}H-^{1}H COSY spectrum of compound **5** (600 MHz, pyridine-d_5)

Figure S59. TOCSY spectrum of compound **5** (600 MHz, pyridine-d_5)

Figure S60. HSQC-TOCSY spectrum of compound **5** (600 MHz, pyridine-d_5)

Figure S61. HRESIMS spectrum of compound **5**

Figure S62. ^{1}H-NMR spectrum of compound **6** (600 MHz, pyridine-d_5)

Figure S63. ^{13}C-NMR spectrum of compound **6** (151 MHz, pyridine-d_5)

Figure S64. HSQC spectrum of compound **6** (600 MHz, pyridine-d_5)

Figure S65. Expanded HSQC spectrum of compound **6** (600 MHz, pyridine-d_5)

Figure S66. HMBC spectrum of compound **6** (600 MHz, pyridine-d_5)

Figure S67. Expanded HMBC spectrum on the glycosidic linkages of compound **6** (600 MHz, pyridine-d_5)

Figure S68. Expanded HMBC spectrum on the ester linkages of compound **6** (600 MHz, pyridine-d_5)

Figure S69. ^{1}H-^{1}H COSY spectrum of compound **6** (600 MHz, pyridine-d_5)

Figure S70. TOCSY spectrum of compound **6** (600 MHz, pyridine-d_5)

Figure S71. HSQC-TOCSY spectrum of compound **6** (600 MHz, pyridine-d_5)

Figure S72. HRESIMS spectrum of compound **6**

Figure S73. ^{1}H-NMR spectrum of compound **7** (600 MHz, pyridine-d_5)

Figure S74. ^{13}C-NMR spectrum of compound **7** (151 MHz, pyridine-d_5)

Figure S75. HSQC spectrum of compound **7** (600 MHz, pyridine-d_5)

Figure S76. Expanded HSQC spectrum of compound **7** (600 MHz, pyridine-d_5)

Figure S77. HMBC spectrum of compound **7** (600 MHz, pyridine-d_5)

Figure S78. Expanded HMBC spectrum on the glycosidic linkages of compound **7** (600 MHz, pyridine-d_5)

Figure S79. Expanded HMBC spectrum on the ester linkages of compound **7** (600 MHz, pyridine-d_5)

Figure S80. ^{1}H-^{1}H COSY spectrum of compound **7** (600 MHz, pyridine-d_5)

Figure S81. TOCSY spectrum of compound **7** (600 MHz, pyridine-d_5)

Figure S82. HSQC-TOCSY spectrum of compound **7** (600 MHz, pyridine-d_5)

Figure S83. HRESIMS spectrum of compound **7**

Figure S84. ^{1}H-NMR spectrum of compound **8** (600 MHz, pyridine-d_5)

Figure S85. ^{13}C-NMR spectrum of compound **8** (151 MHz, pyridine-d_5)

Figure S86. HSQC spectrum of compound **8** (600 MHz, pyridine-d_5)

Figure S87. Expanded HSQC spectrum of compound **8** (600 MHz, pyridine-d_5)

Figure S88. HMBC spectrum of compound **8** (600 MHz, pyridine-d_5)

Figure S89. Expanded HMBC spectrum on the glycosidic linkages of compound **8** (600 MHz, pyridine-d_5)

Figure S90. Expanded HMBC spectrum on the ester linkages of compound **8** (600 MHz, pyridine-d_5)

Figure S91. ^{1}H-^{1}H COSY spectrum of compound **8** (600 MHz, pyridine-d_5)

Figure S92. TOCSY spectrum of compound **8** (600 MHz, pyridine-d_5)

Figure S93. HSQC-TOCSY spectrum of compound **8** (600 MHz, pyridine-d_5)

Figure S94. HRESIMS spectrum of compound **8**

Table S1 ^{1}H and ^{13}C NMR spectroscopic data of compound **8** (600 and 151 MHz, respectively, in pyridine-d_5)[a]

position[b]	**8**	
	δ_H	δ_C
Glc-1	5.01 d (7.2)	101.4
2	4.26	77.7
3	4.29 t (9.0)	80.1
4	4.17	72.5
5	3.92 m	78.7
6	4.37 dd (5.4,	63.3
	4.53	
Rha-1	6.37 br s	102.1
2	4.70 br s	73.2
3	4.64 dd (3.0, 9.0)	73.2
4	4.30	82.7
5	4.94 m	68.2
6	1.73 d (6.6)	19.4
Rha′-1	5.92 br d (1.8)	104.0
2	5.22 br s	72.5
3	4.76 dd (3.0, 9.0)	83.2
4	4.53 t (9.0)	79.0
5	4.44 m	69.0
6	1.63 d (6.6)	19.4
Rha″-1	6.25 br s	103.6
2	4.90 br s	73.1
3	4.45	73.2
4	4.25 t (9.0)	74.4
5	4.33 m	70.9
6	1.59 d (6.6)	18.8
Glc-1	5.26 d (7.8)	106.0
2	3.99 br t (8.4)	75.6
3	4.21	78.9
4	4.14	72.2
5	3.97 m	79.1
6	4.30	63.4
	4.56	
Ag-1		174.5
2	2.33 t (7.8)	34.6
11	4.07 m	78.4
12	1.80	35.9

13	1.67	25.8
	1.56	
14	1.34	33.0
15	1.35	23.5
16	0.92 t (7.2)	14.9
-OCH_3	3.62 s	51.7

[a] Overlapped signals are reported without designating multiplicity; [b]Abbreviations: Glc = glucopyranosyl, Rha = rhamnopyranosyl, Ag = 11-hydroxyhexadecanoyl.

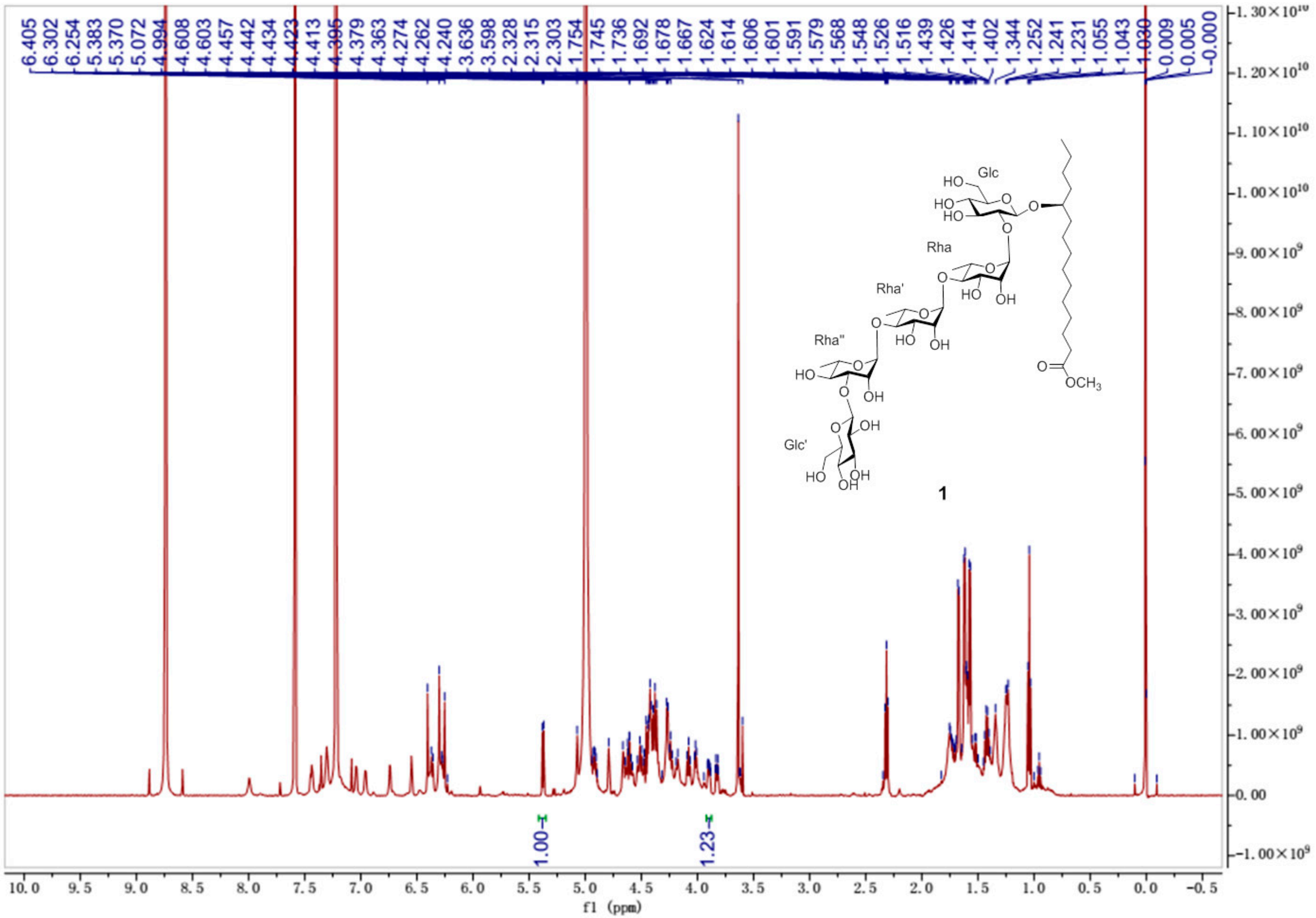

Figure S1. ^{1}H-NMR spectrum of compound **1** (600 MHz, pyridine-d_5)

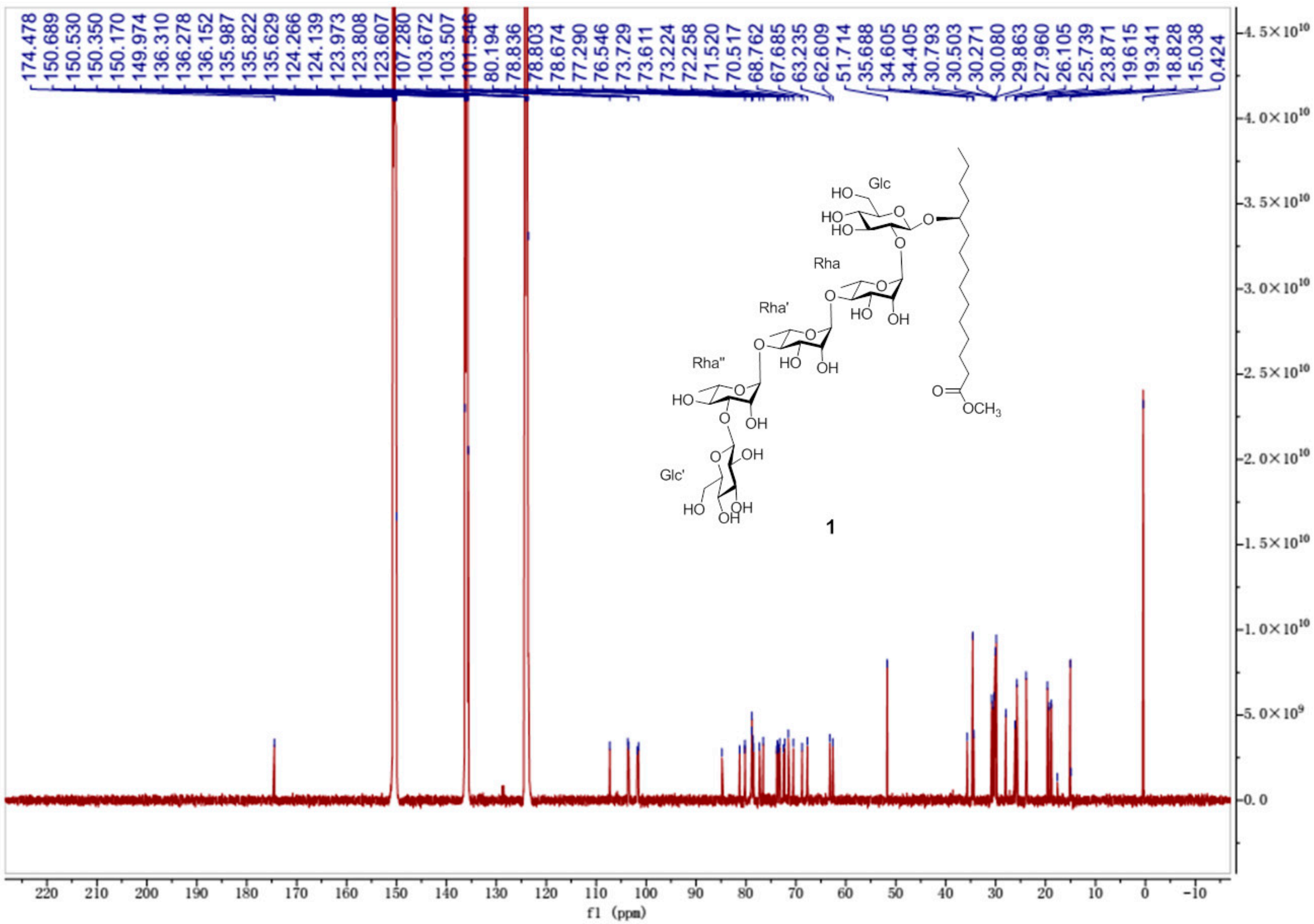

Figure S2. ^{13}C-NMR spectrum of compound **1** (151 MHz, pyridine-d_5)

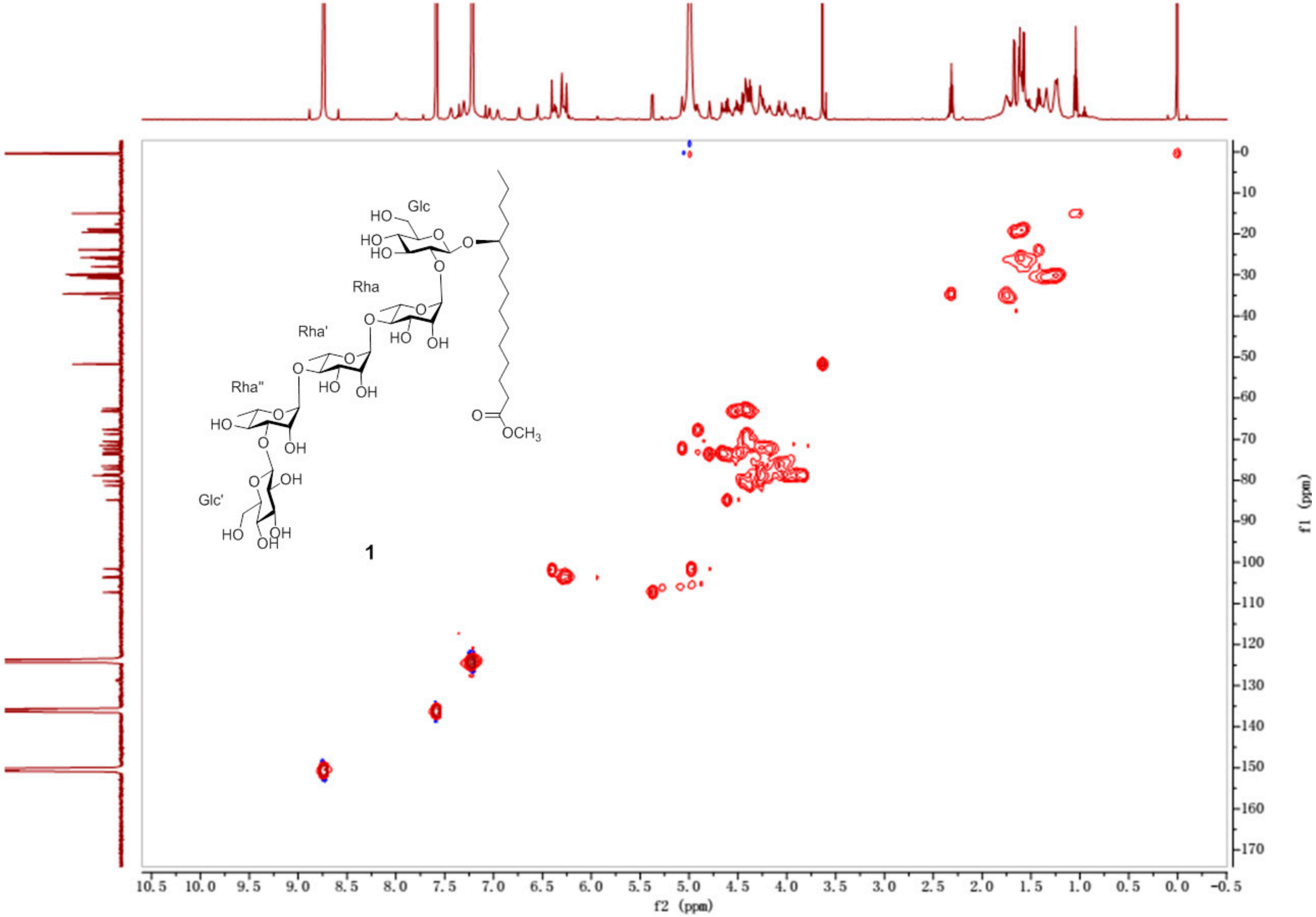

Figure S3. HSQC spectrum of compound **1** (600 MHz, pyridine-d_5)

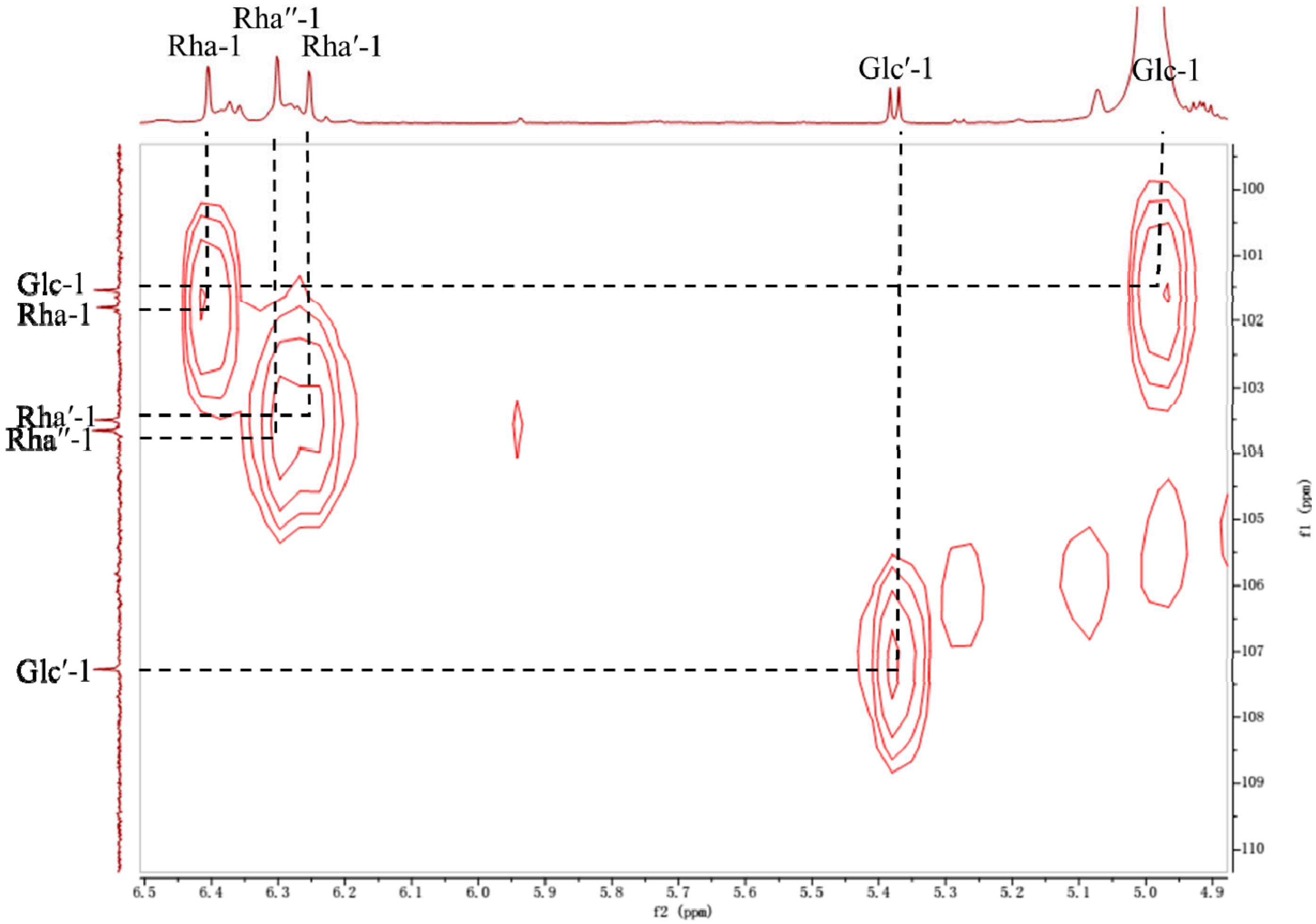

Figure S4. Expanded HSQC spectrum of compound **1** (600 MHz, pyridine-d_5)

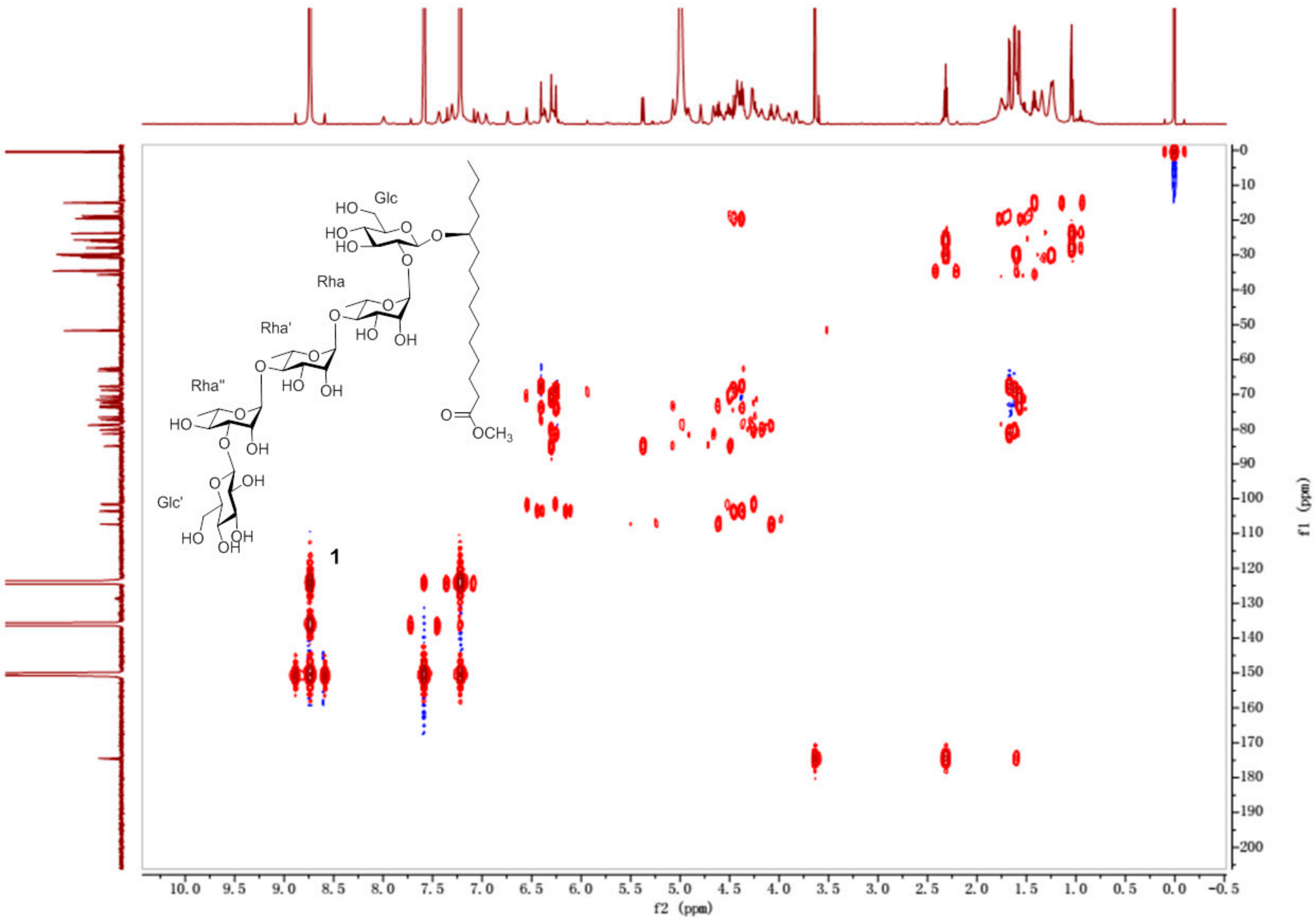

Figure S5. HMBC spectrum of compound **1** (600 MHz, pyridine-d_5)

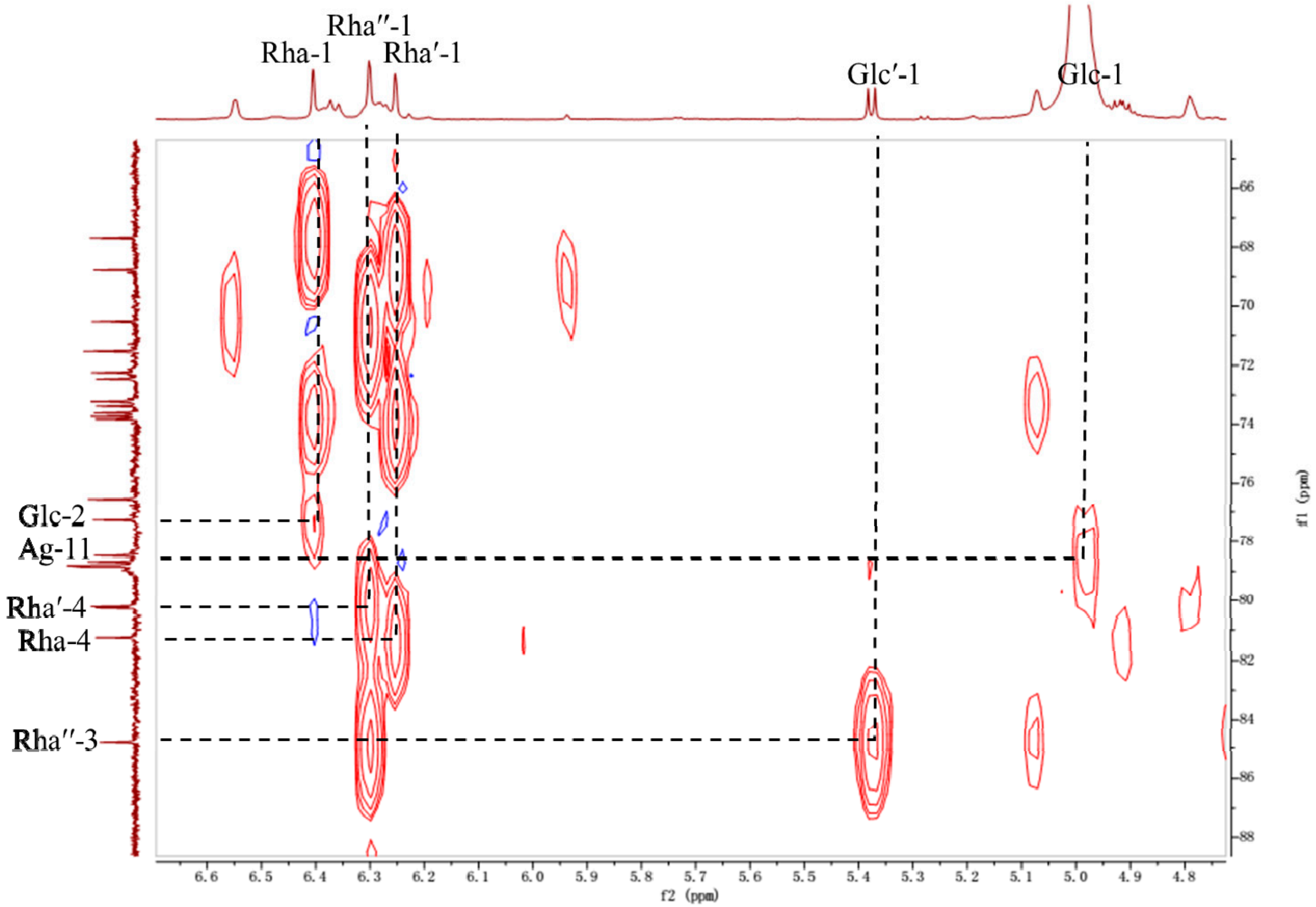

Figure S6. Expanded HMBC spectrum on the glycosidic linkages of compound **1** (600 MHz, pyridine-d_5)

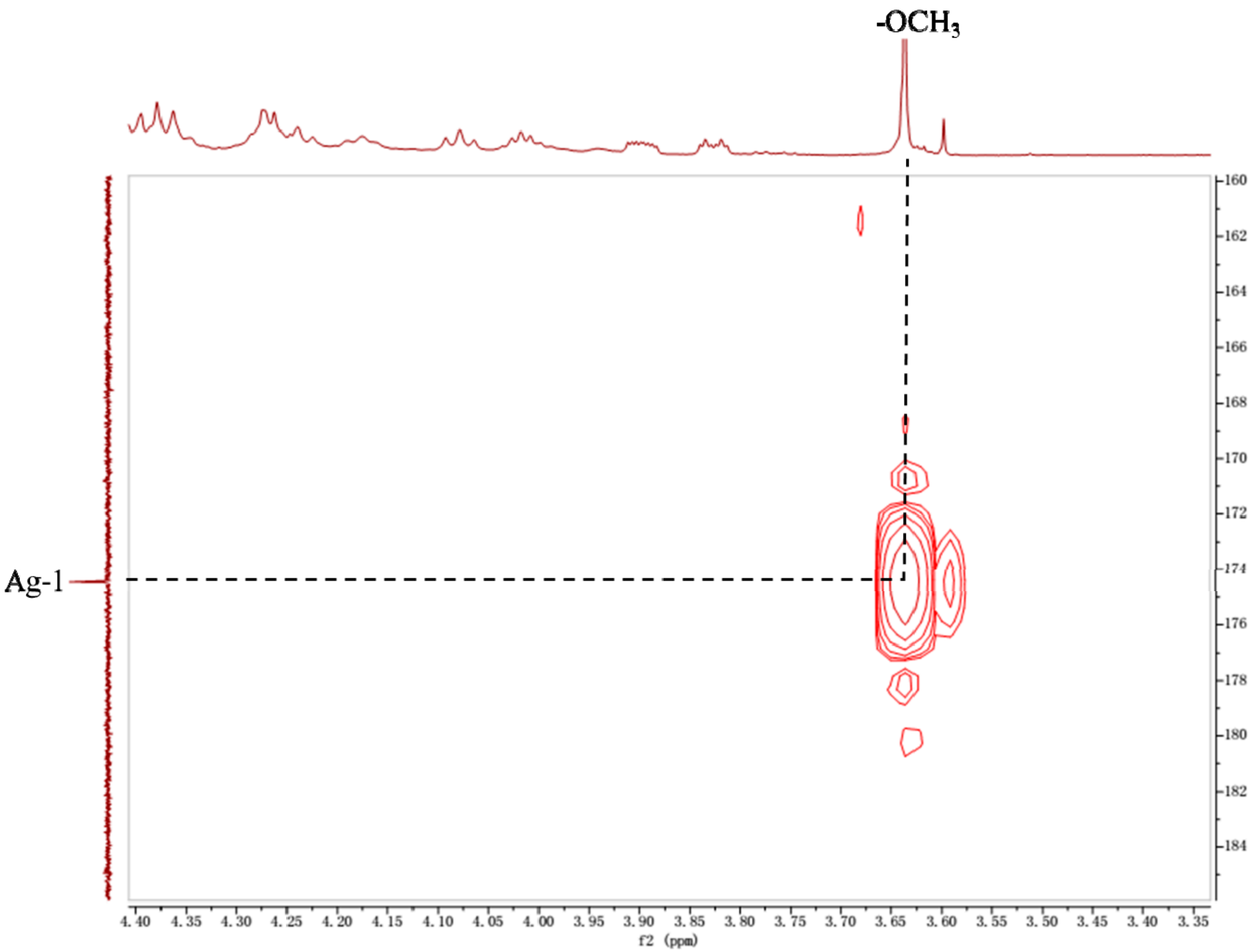

Figure S7. Expanded HMBC spectrum on the ester linkages of compound **1** (600 MHz, pyridine-*d*$_5$)

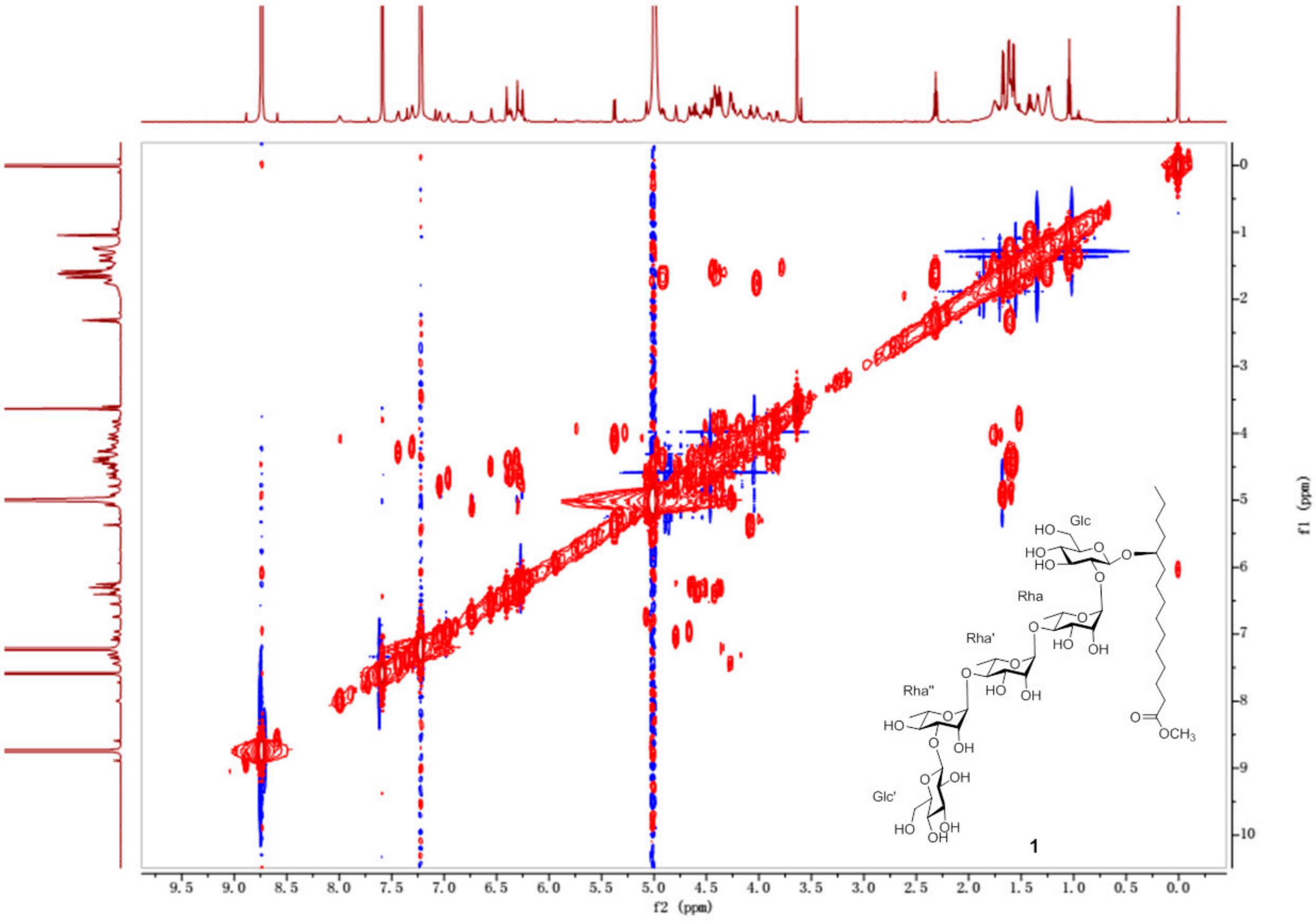

Figure S8. ^{1}H-^{1}H COSY spectrum of compound **1** (600 MHz, pyridine-d_5)

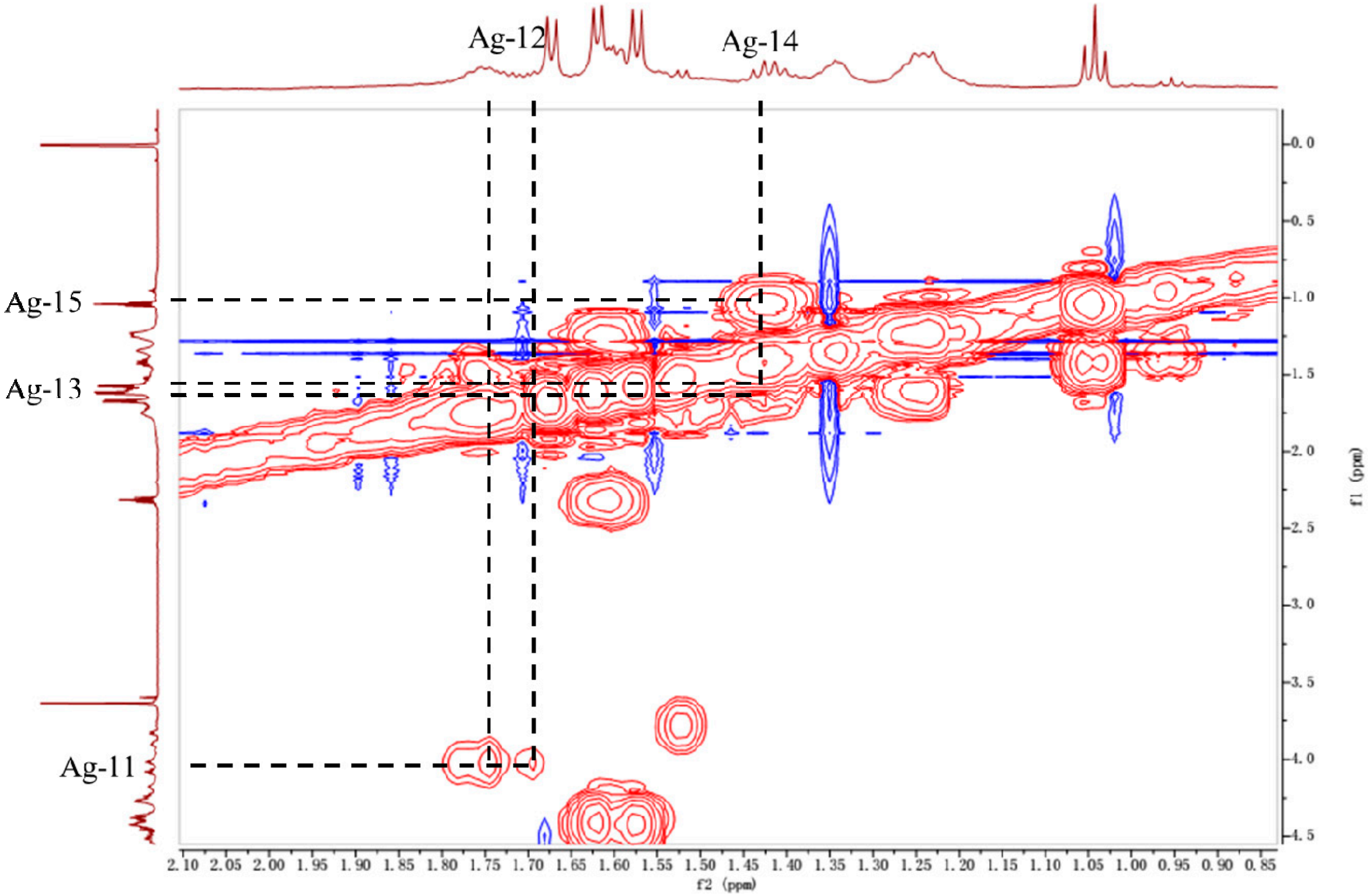

Figure S9. Expanded ^{1}H-^{1}H COSY spectrum of compound **1** (600 MHz, pyridine-d_5)

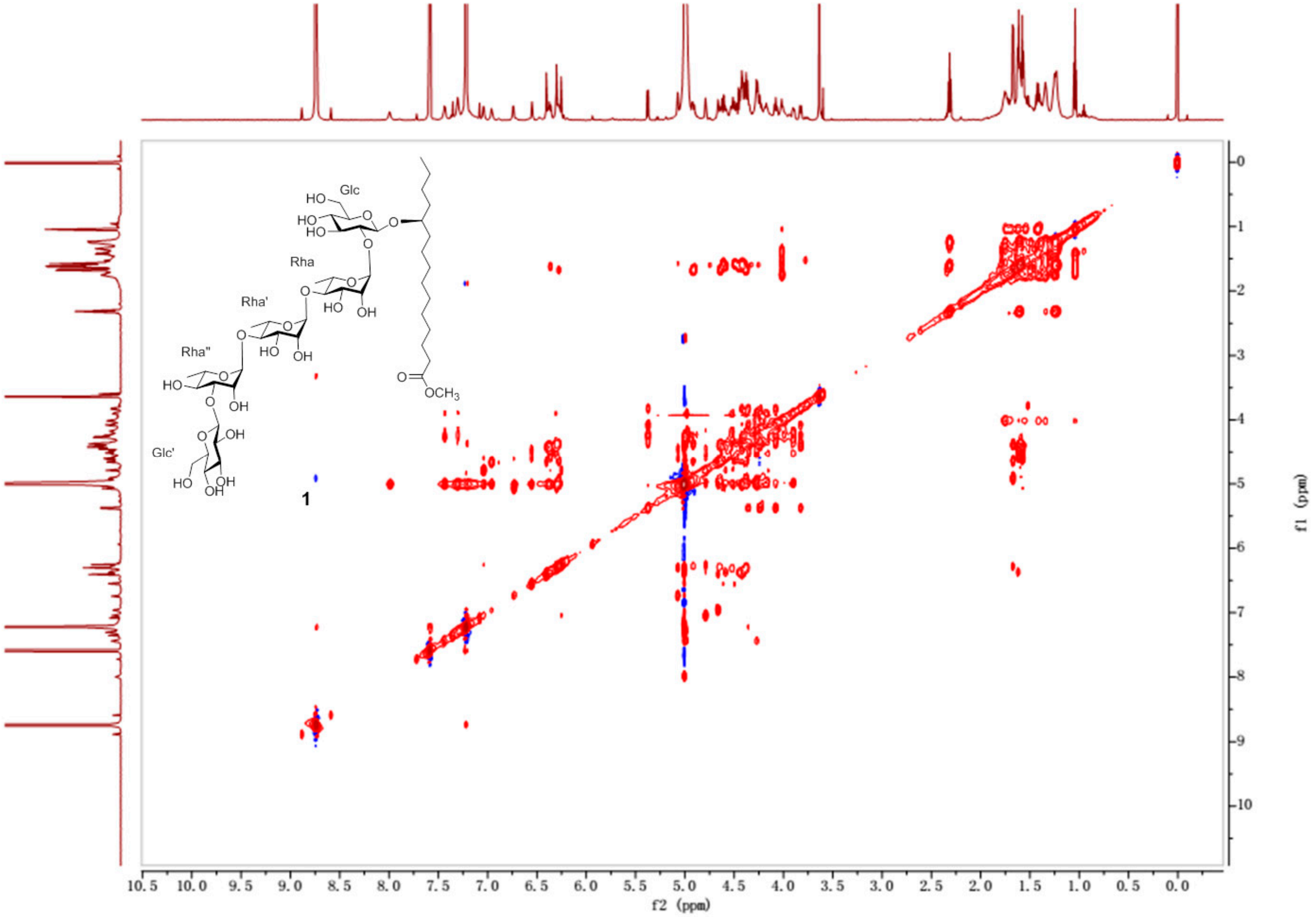

Figure S10. TOCSY spectrum of compound **1** (600 MHz, pyridine-d_5)

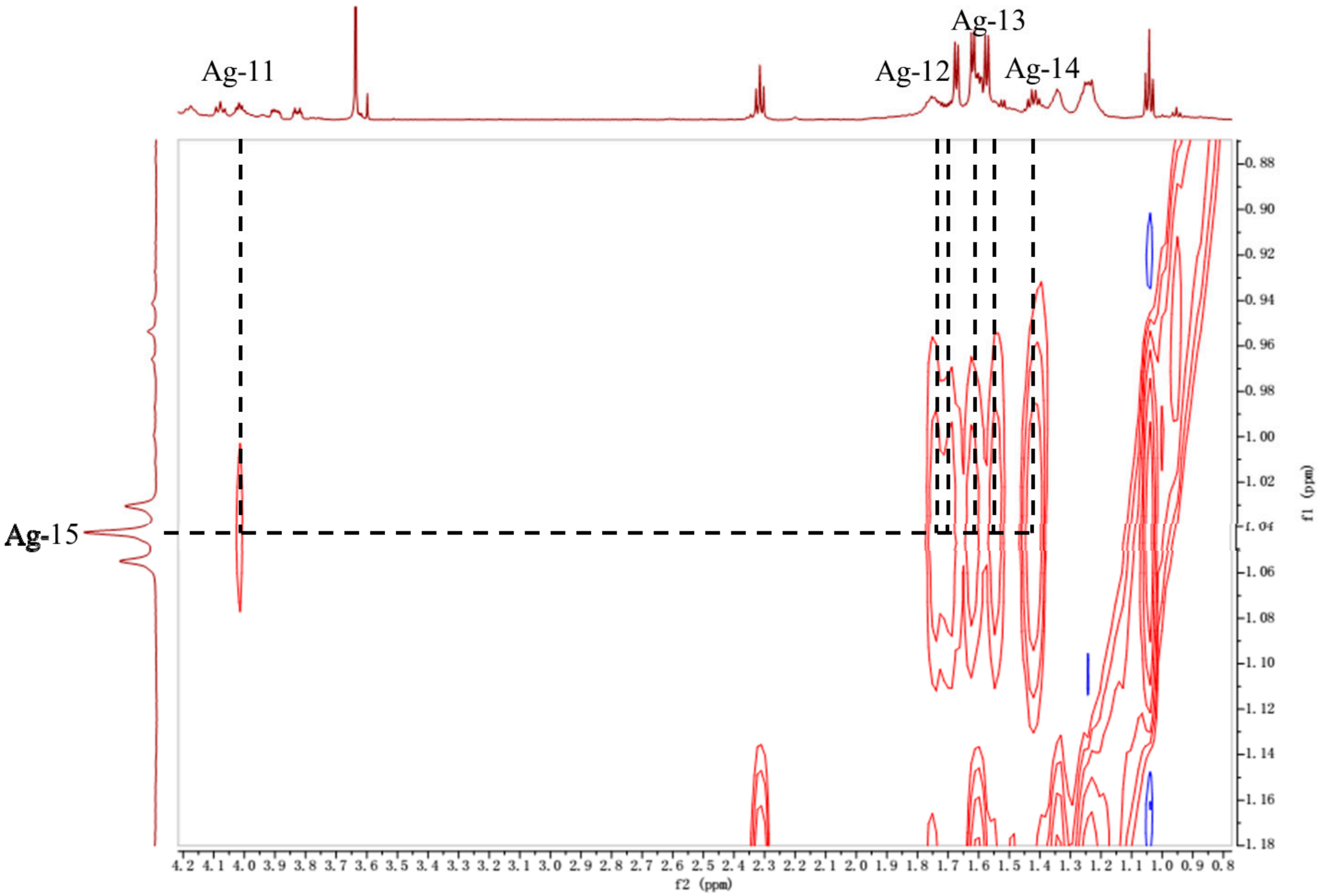

Figure S11. Expanded TOCSY spectrum of compound **1** (600 MHz, pyridine-d_5)

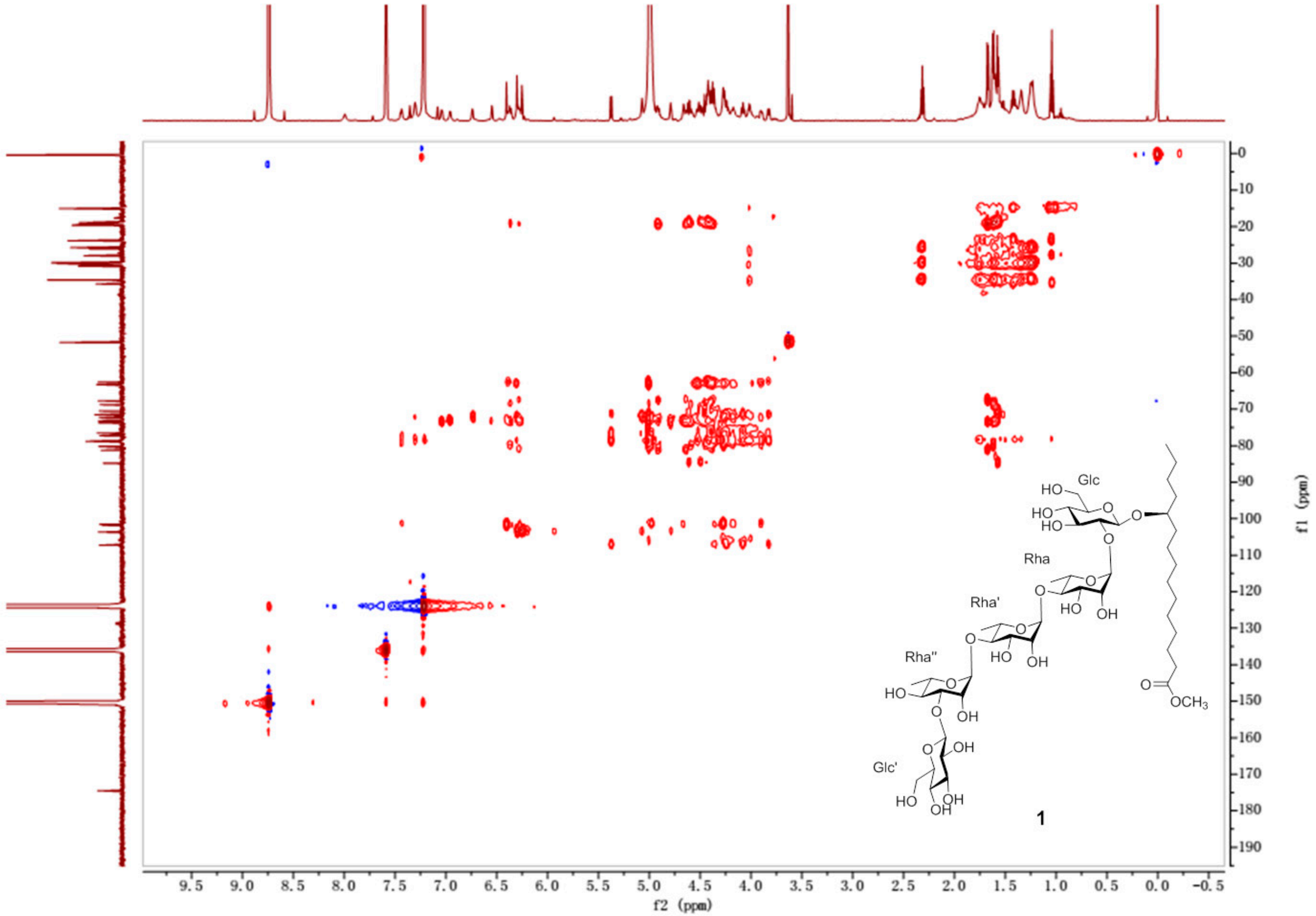

Figure S12. HSQC-TOCSY spectrum of compound **1** (600 MHz, pyridine-d_5)

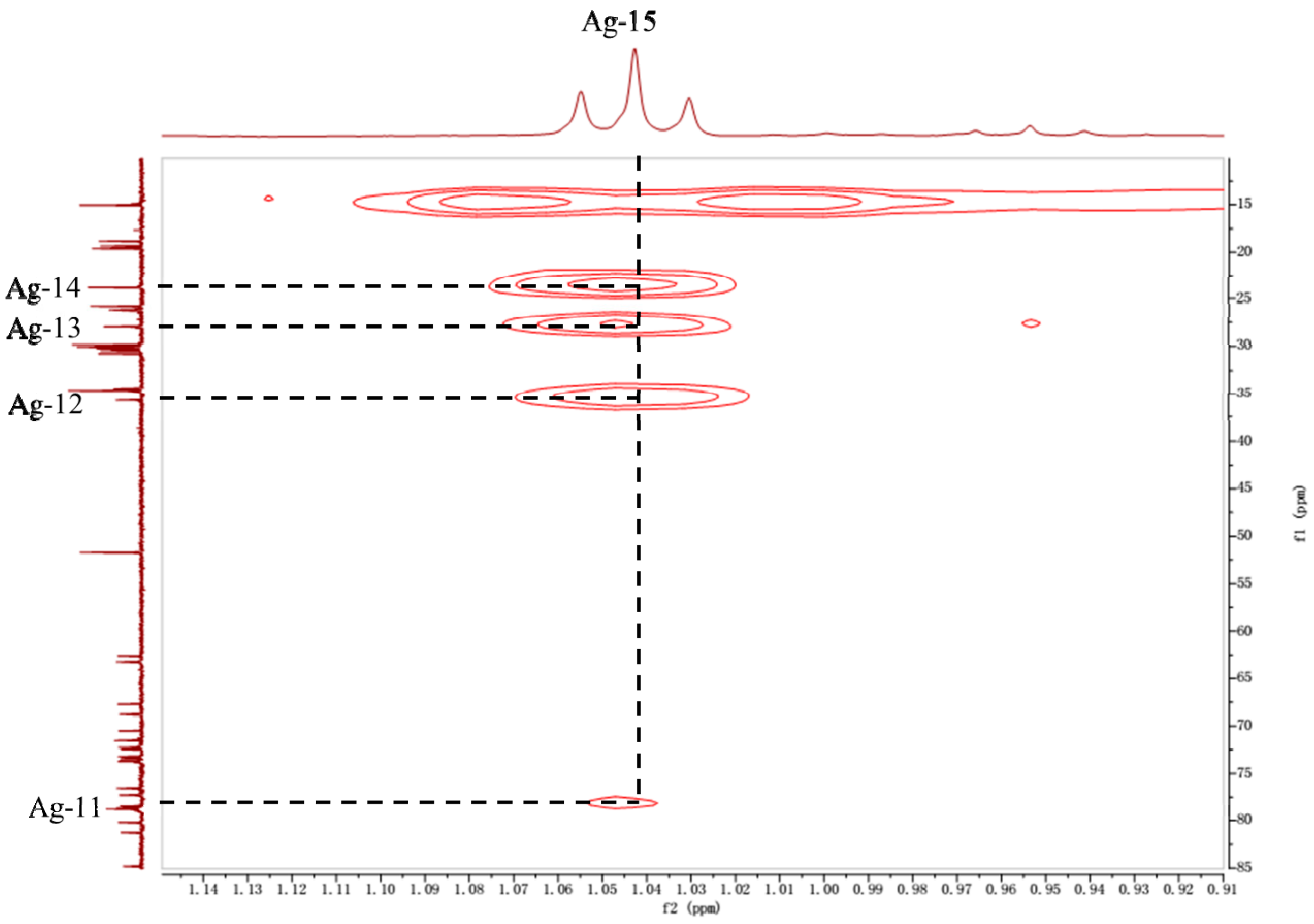

Figure S13. Expanded HSQC-TOCSY spectrum of compound **1** (600 MHz, pyridine-d_5)

Single Mass Analysis
Tolerance = 5.0 mDa / DBE: min = -1.5, max = 50.0
Element prediction: Off
Number of isotope peaks used for i-FIT = 3

Monoisotopic Mass, Even Electron Ions
9722 formula(e) evaluated with 1 results within limits (up to 50 closest results for each mass)
Elements Used:
C: 46-46 H: 81-81 N: 0-100 O: 0-100 Na: 0-4
4--P--N
240906-1-262-2-JDT-41------------neg 20 (0.158) 1: TOF MS ES- 5.02e+004

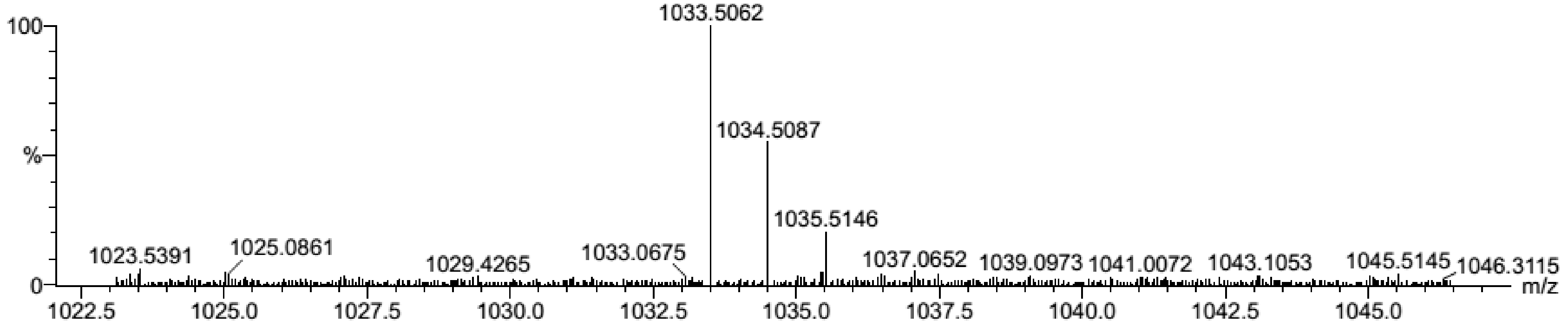

Minimum:				-1.5				
Maximum:		5.0	10.0	50.0				

Mass	Calc. Mass	mDa	PPM	DBE	i-FIT	Norm	Conf(%)	Formula
1033.5062	1033.5067	-0.5	-0.5	6.5	670.6	n/a	n/a	C46 H81 O25

Figure S14. HRESIMS spectrum of compound **1**

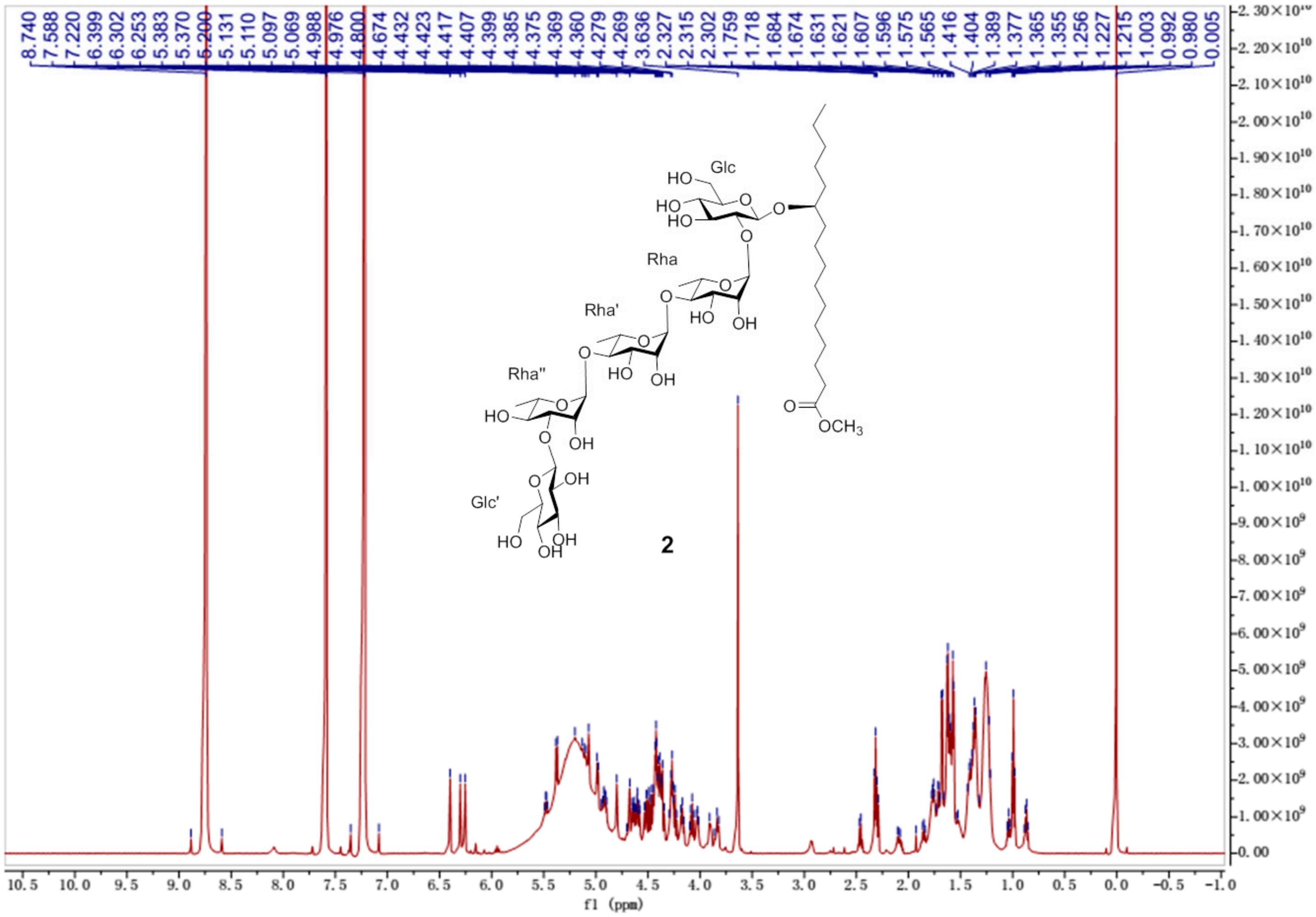

Figure S15. ^{1}H-NMR spectrum of compound **2** (600 MHz, pyridine-d_5)

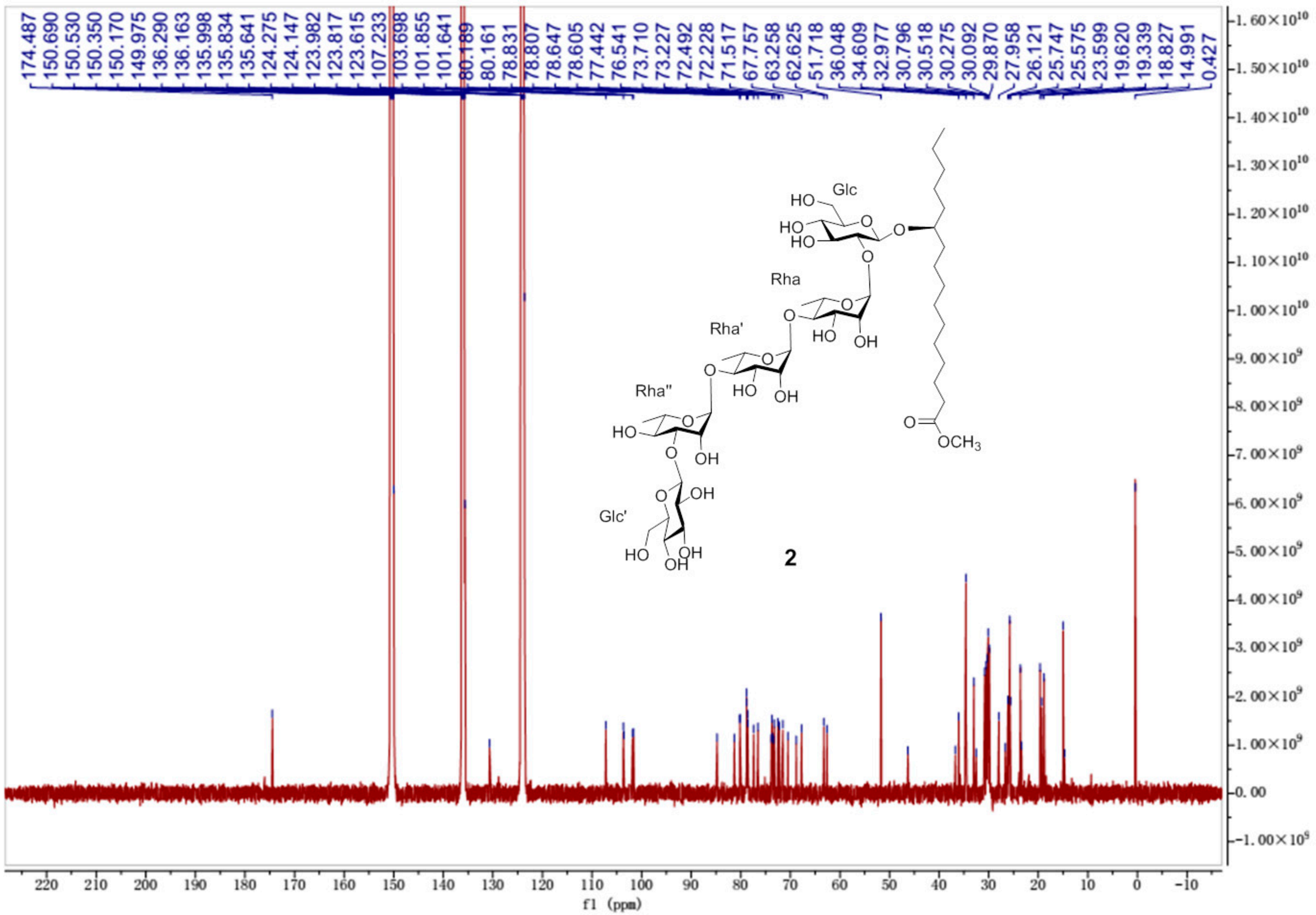

Figure S16. ^{13}C-NMR spectrum of compound **2** (151 MHz, pyridine-d_5)

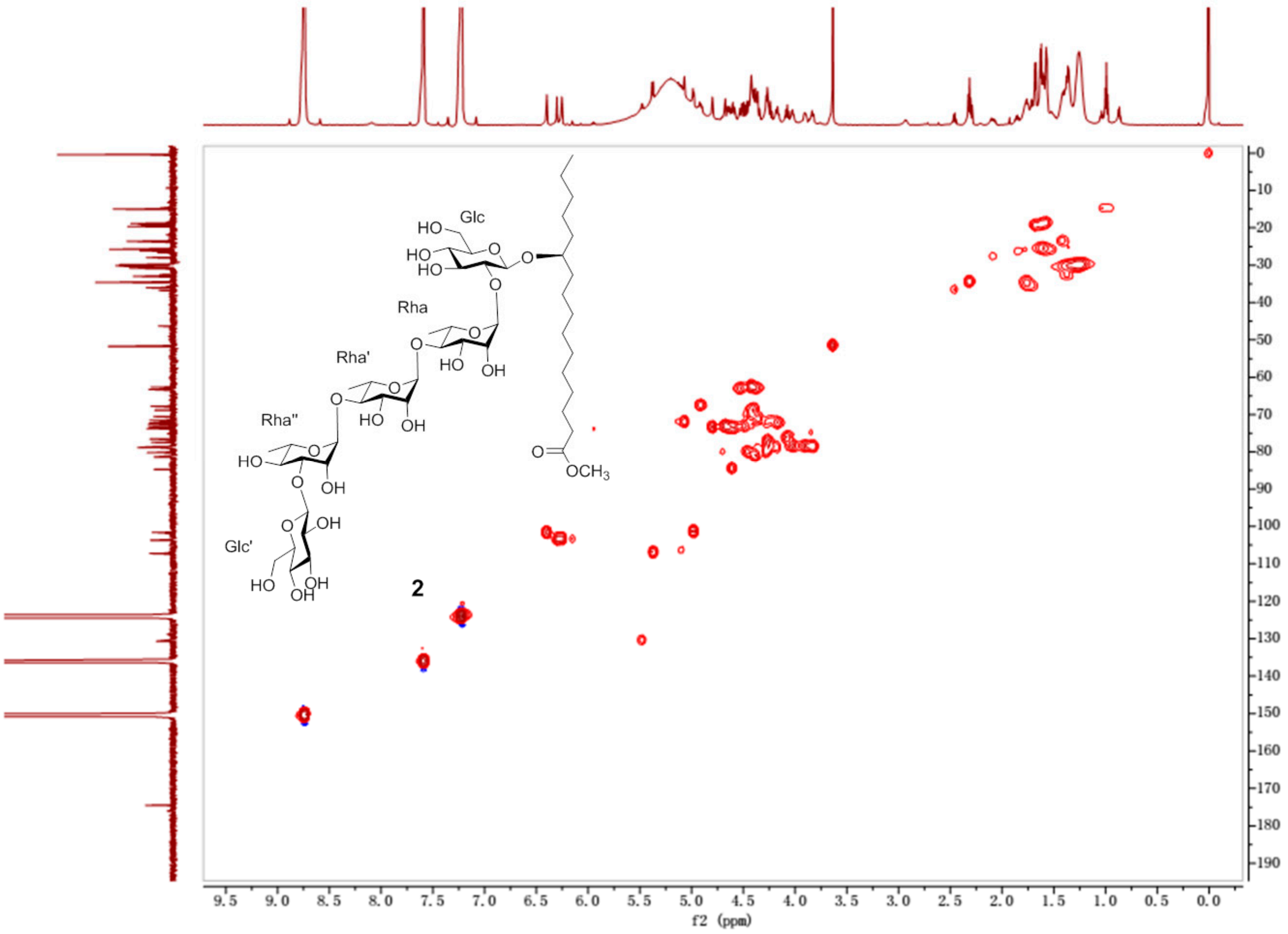

Figure S17. HSQC spectrum of compound **2** (600 MHz, pyridine-d_5)

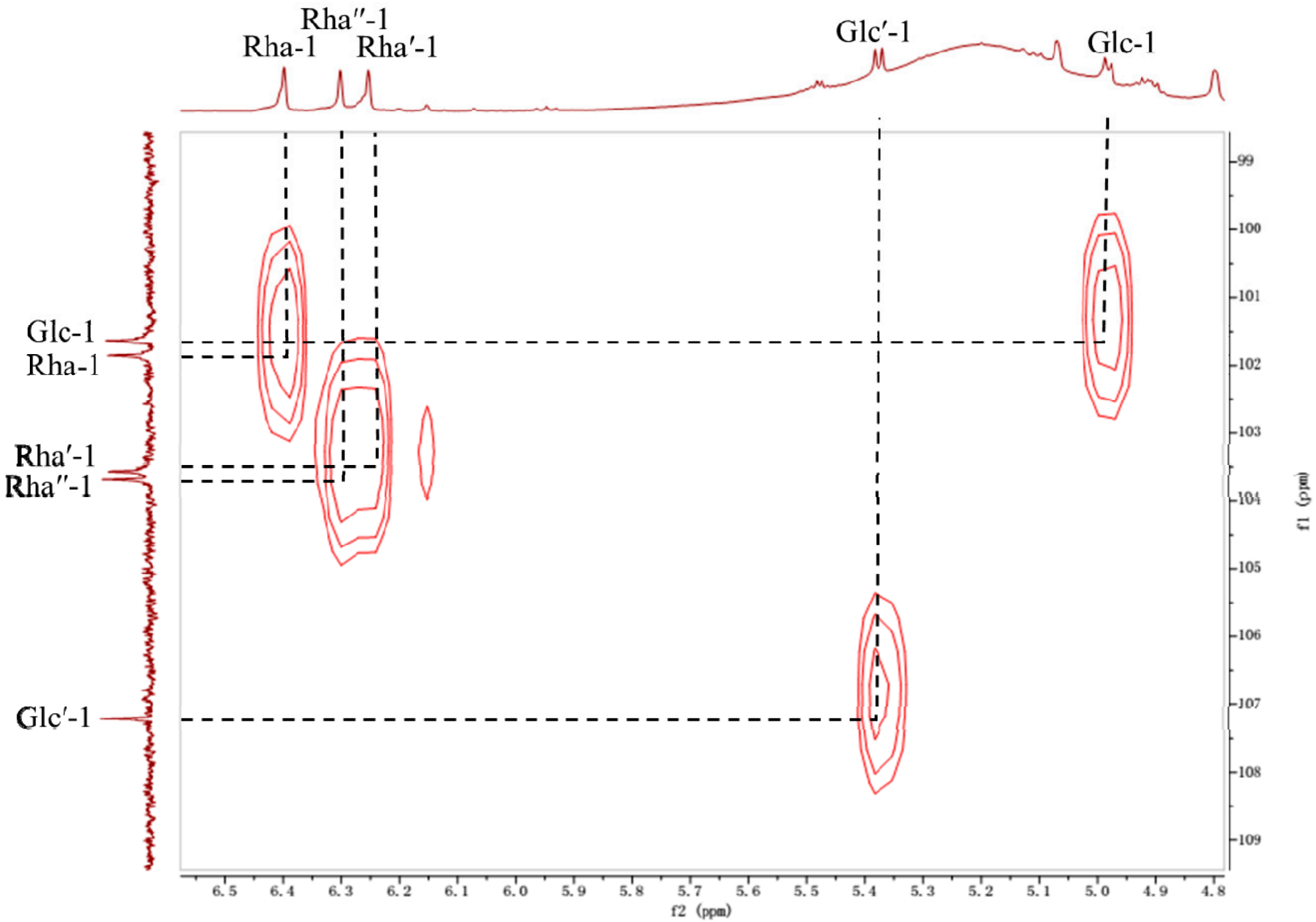

Figure S18. Expanded HSQC spectrum of compound **2** (600 MHz, pyridine-d_5)

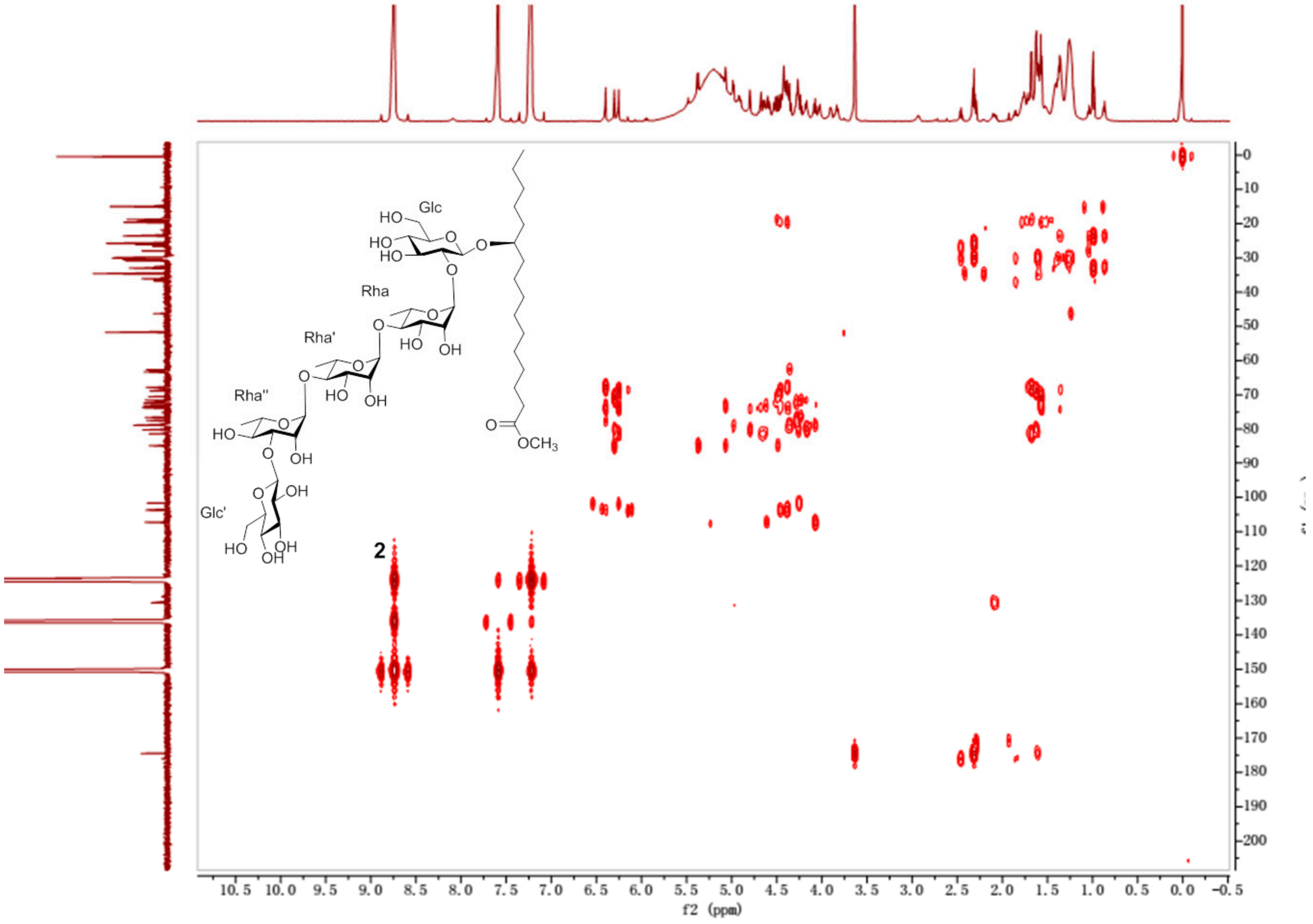

Figure S19. HMBC spectrum of compound **2** (600 MHz, pyridine-d_5)

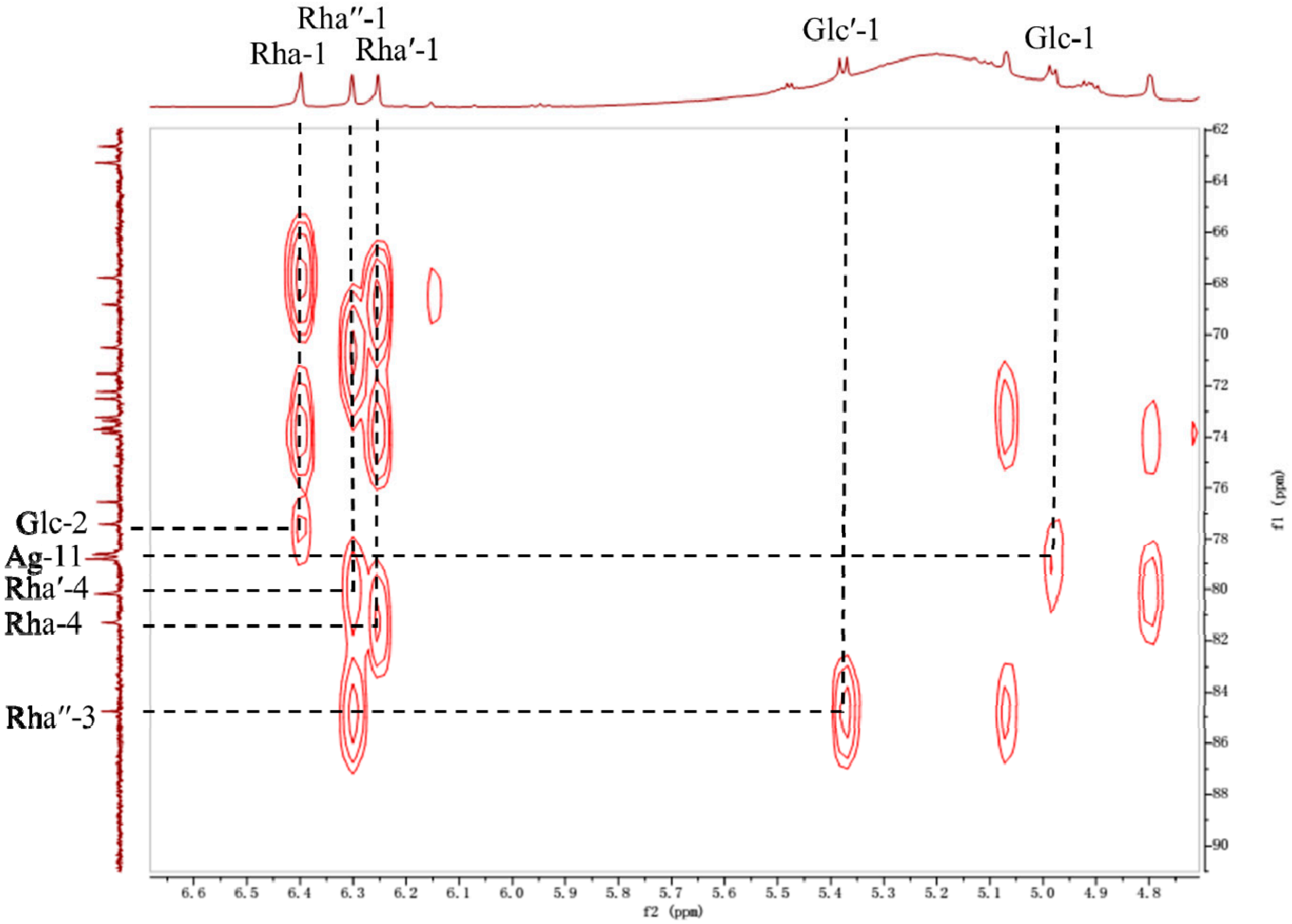

Figure S20. Expanded HMBC spectrum on the glycosidic linkages of compound **2** (600 MHz, pyridine-d_5)

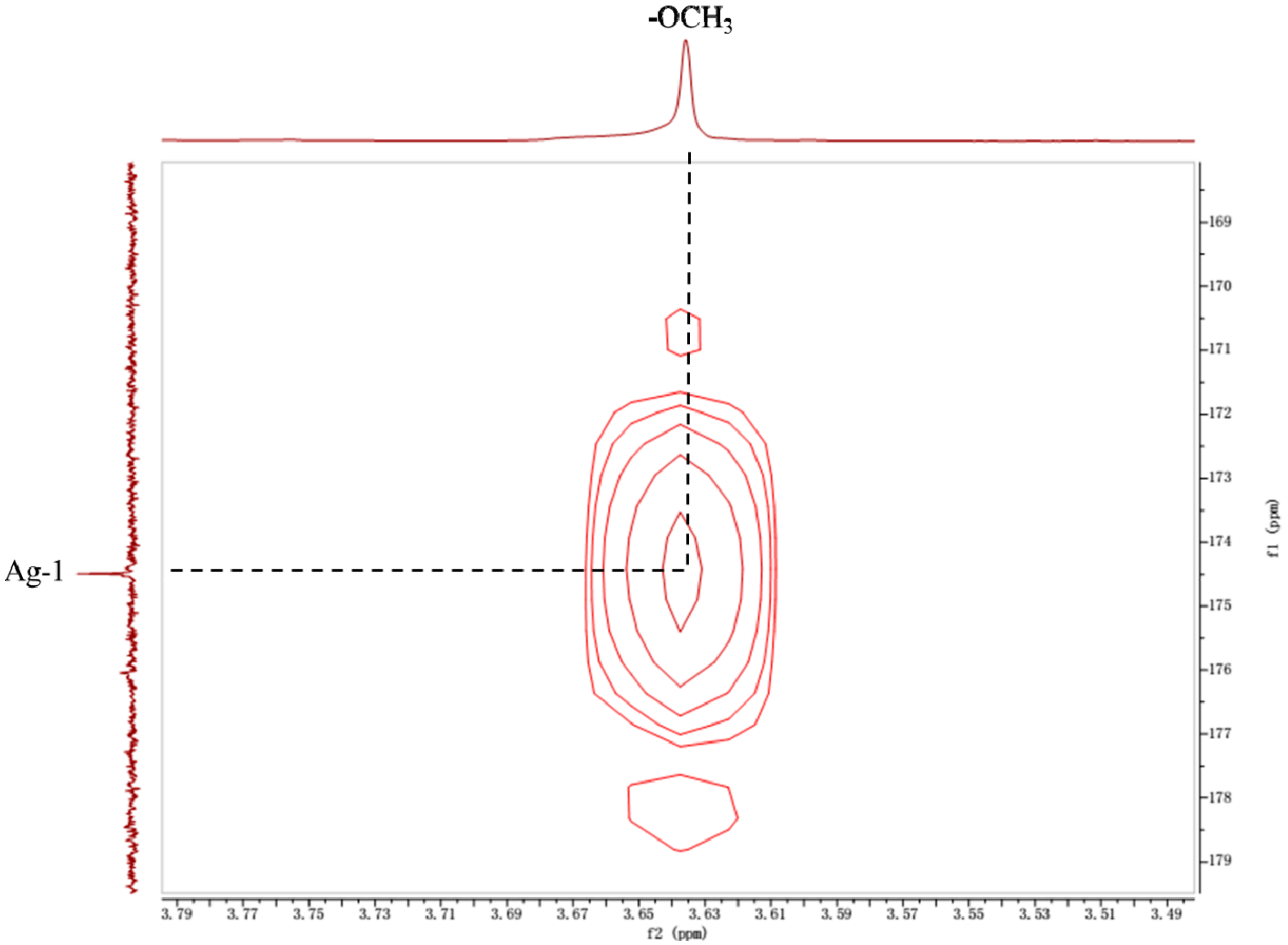

Figure S21. Expanded HMBC spectrum on the ester linkages of compound **2** (600 MHz, pyridine-*d*5)

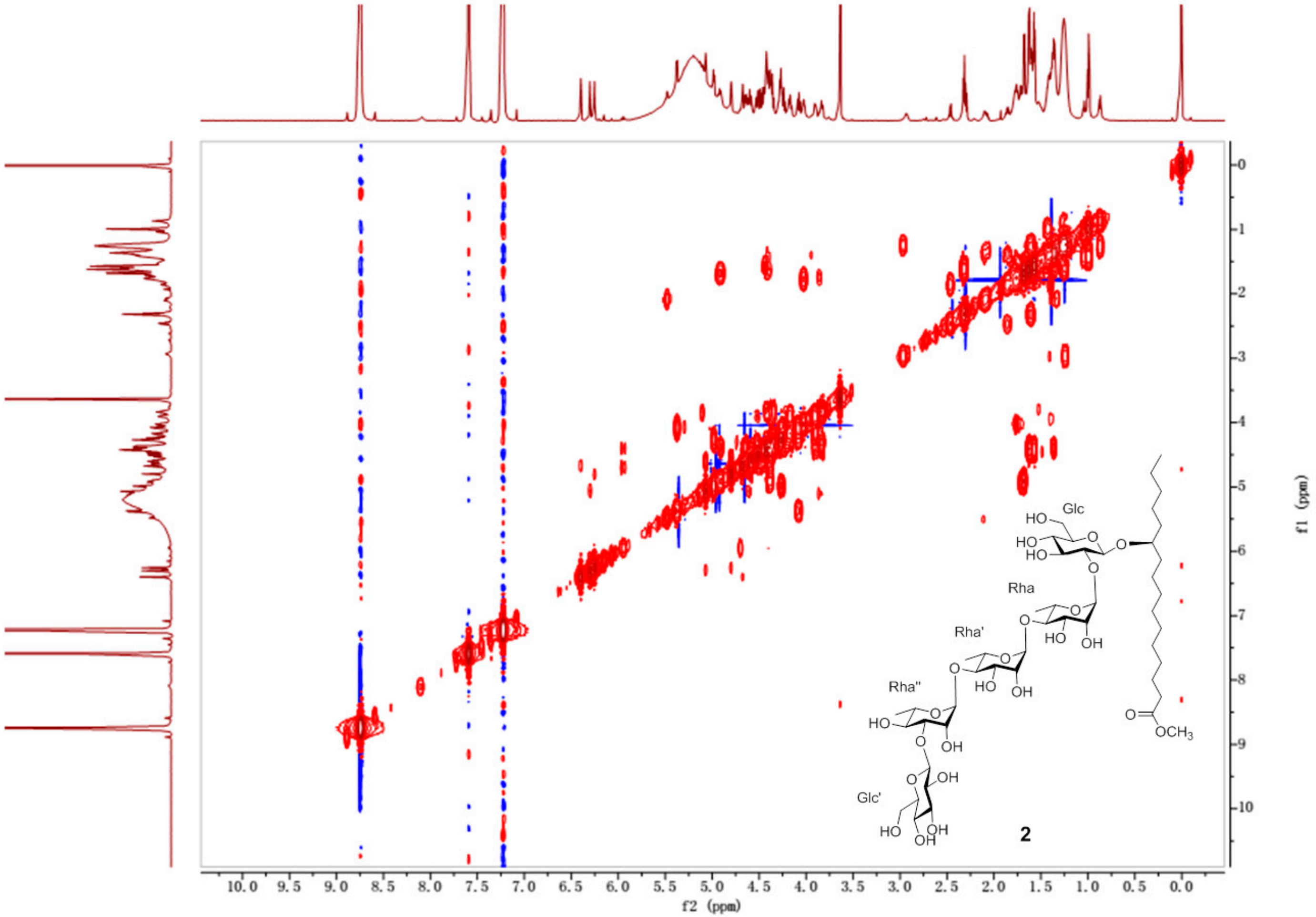

Figure S22. ^{1}H-^{1}H COSY spectrum of compound **2** (600 MHz, pyridine-d_5)

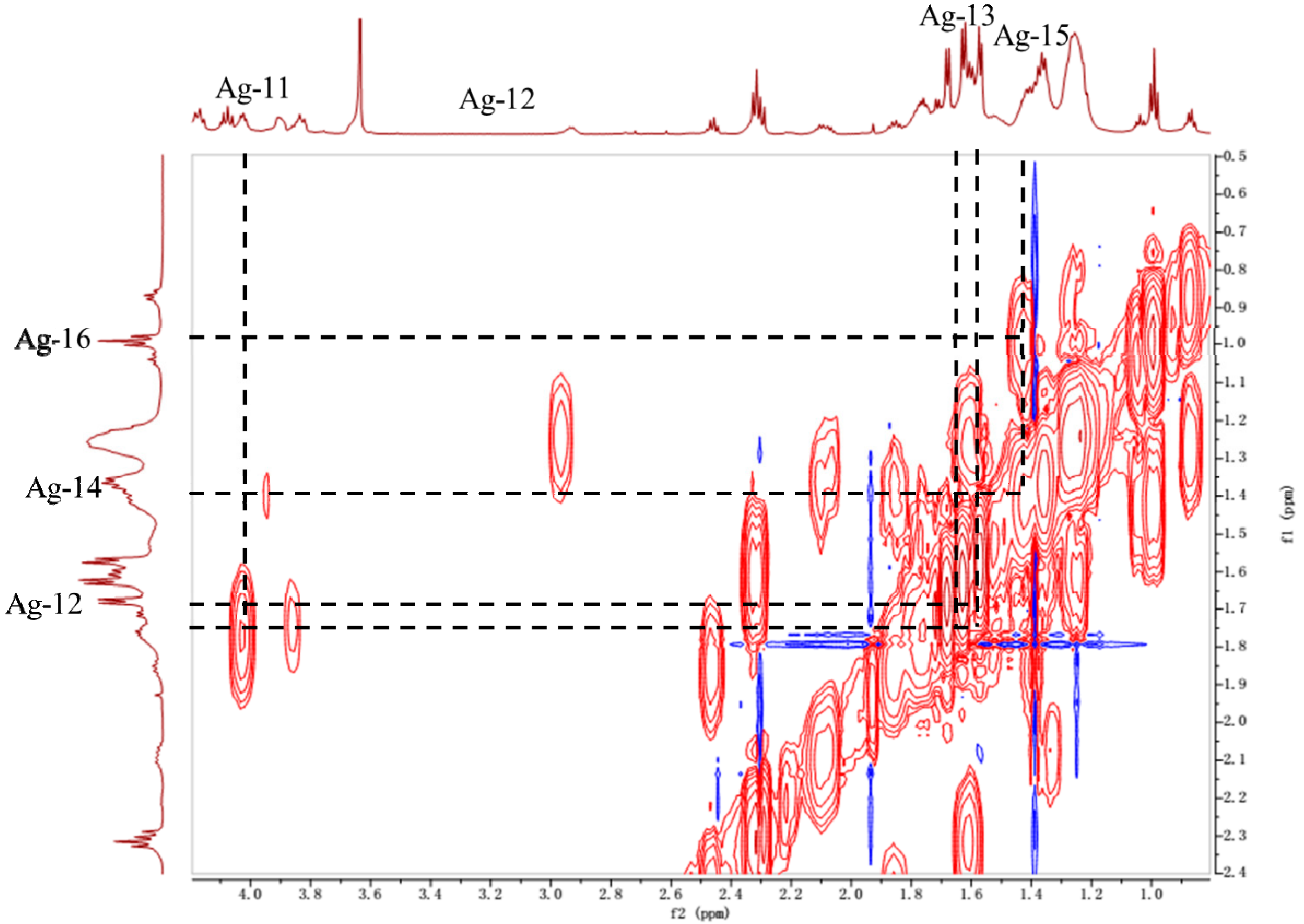

Figure S23. Expanded ^1H-^1H COSY spectrum of compound **2** (600 MHz, pyridine-d_5)

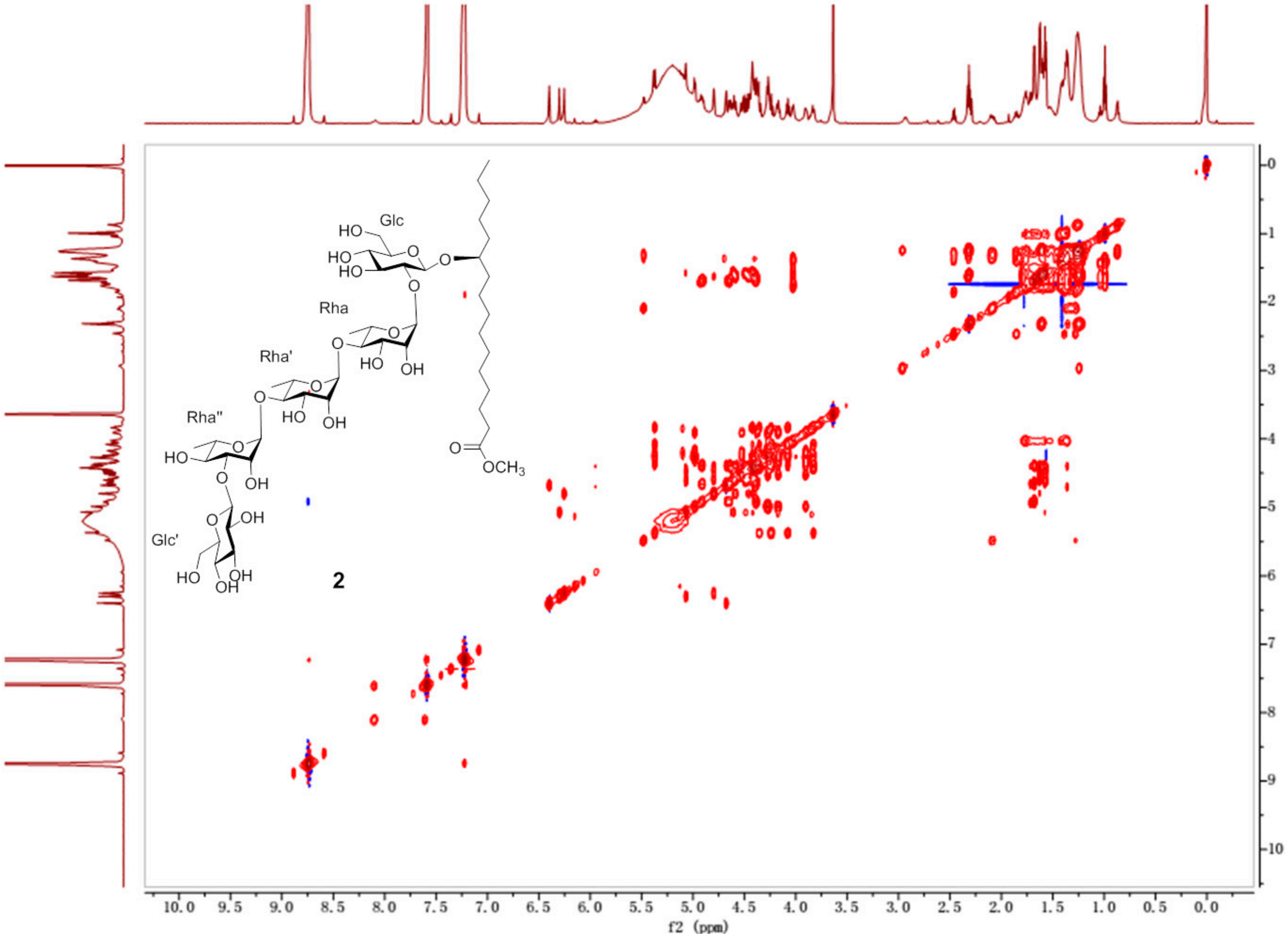

Figure S24. TOCSY spectrum of compound **2** (600 MHz, pyridine-d_5)

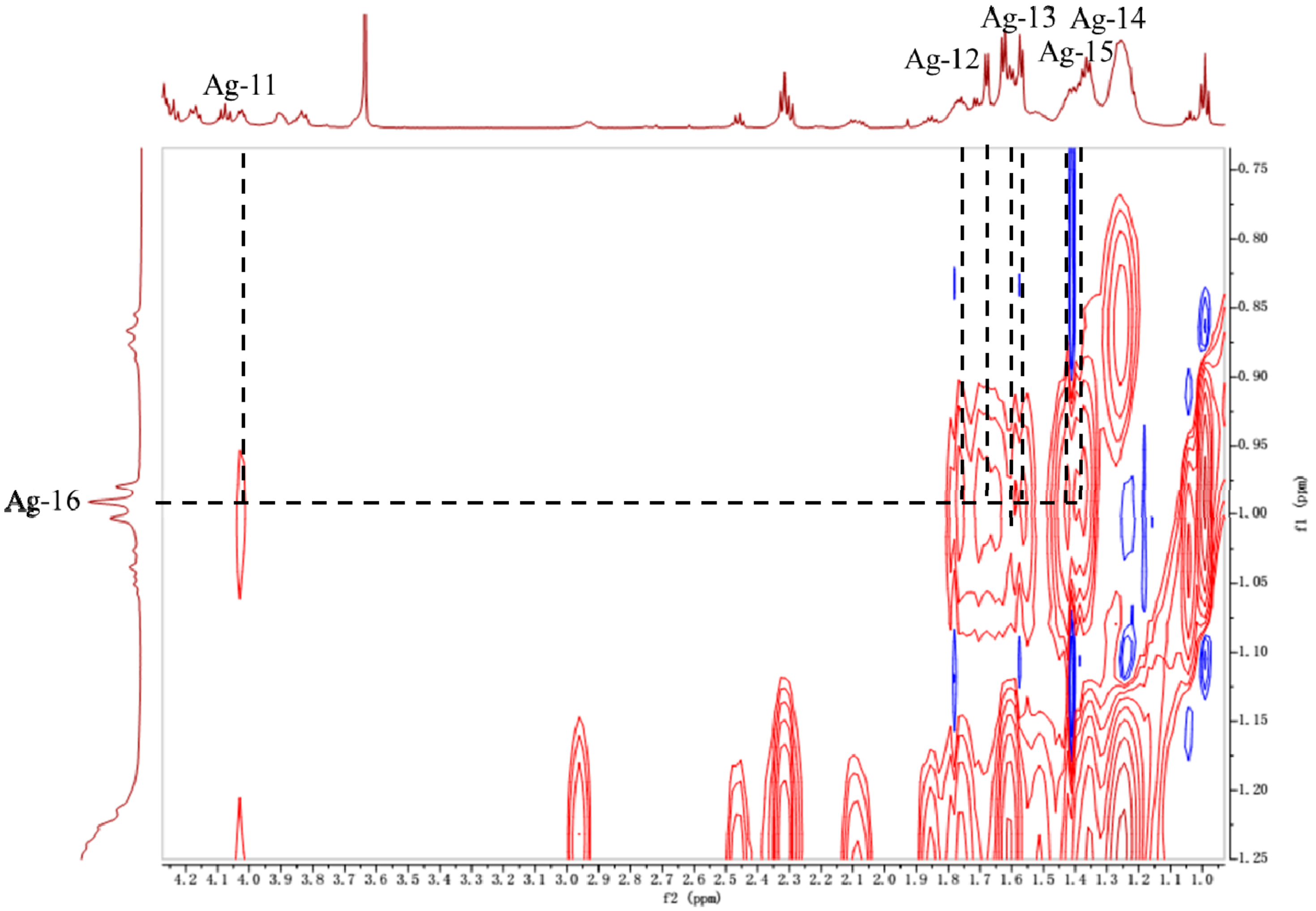

Figure S15. Expanded TOCSY spectrum of compound **2** (600 MHz, pyridine-*d*5)

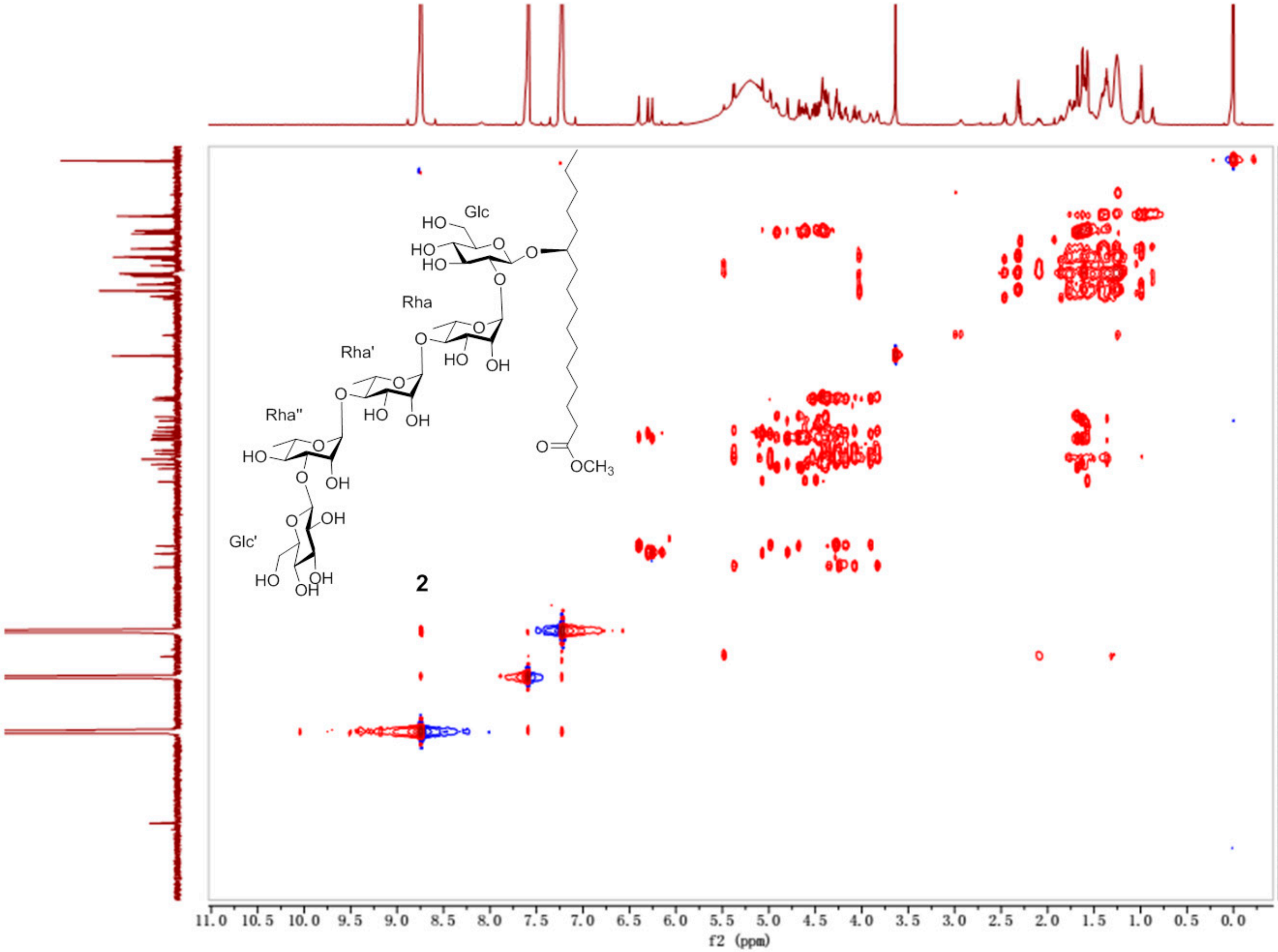

Figure S26. HSQC-TOCSY spectrum of compound **2** (600 MHz, pyridine-d_5)

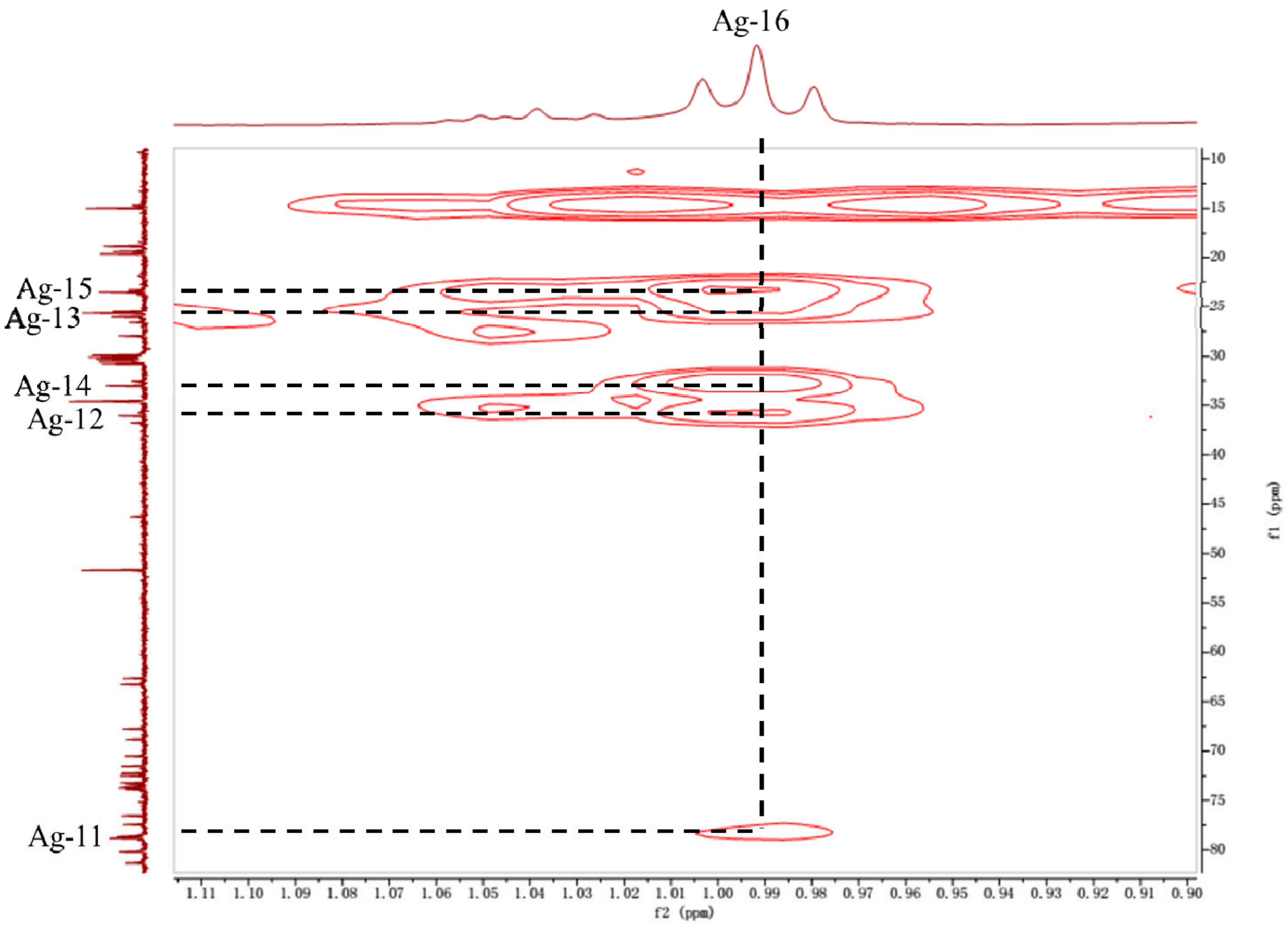

Figure S27. Expanded HSQC-TOCSY spectrum of compound **2** (600 MHz, pyridine-*d*5)

Elemental Composition Report **Page 1**

Single Mass Analysis
Tolerance = 5.0 mDa / DBE: min = -1.5, max = 50.0
Element prediction: Off
Number of isotope peaks used for i-FIT = 3

Monoisotopic Mass, Even Electron Ions
6525 formula(e) evaluated with 1 results within limits (up to 50 closest results for each mass)
Elements Used:
C: 47-47 H: 84-84 N: 0-100 O: 0-100 Na: 0-2
1--P--N
240810-8-250-2-JDT-39 8 (0.079)

1: TOF MS ES+
1.36e+006

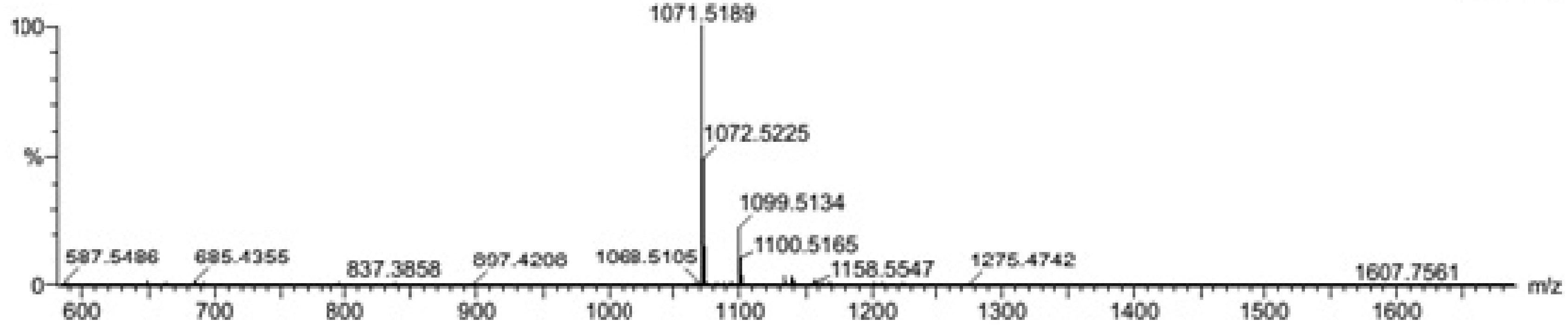

Minimum:				-1.5				
Maximum:		5.0	10.0	50.0				

Mass	Calc. Mass	mDa	PPM	DBE	i-FIT	Norm	Conf(%)	Formula
1071.5189	1071.5199	-1.0	-0.9	5.5	552.0	n/a	n/a	C47 H84 O25 Na

Figure S28. HRESIMS spectrum of compound **2**

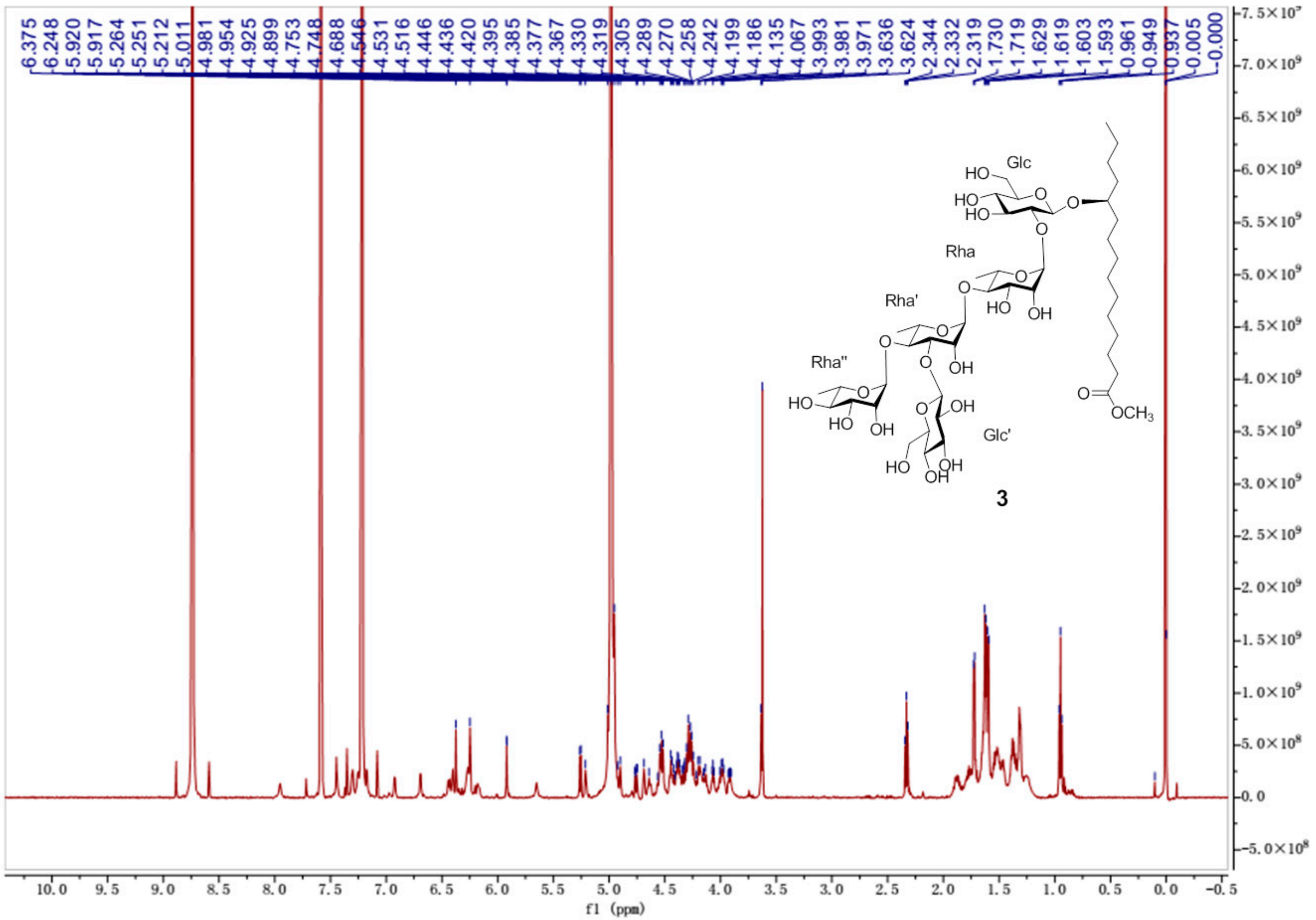

Figure S29. ^{1}H-NMR spectrum of compound **3** (600 MHz, pyridine-d_5)

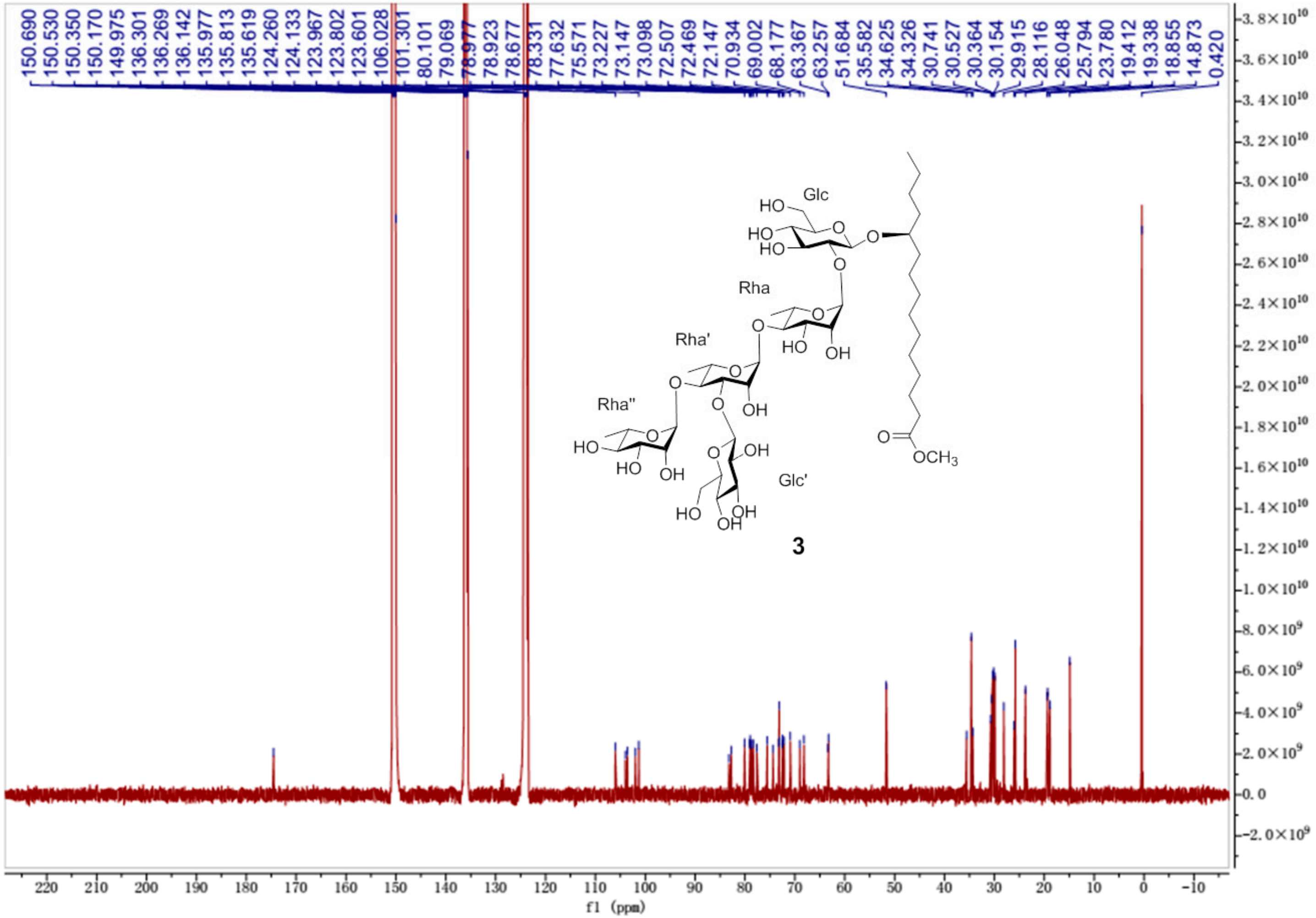

Figure S30. ^{13}C-NMR spectrum of compound **3** (151 MHz, pyridine-d_5)

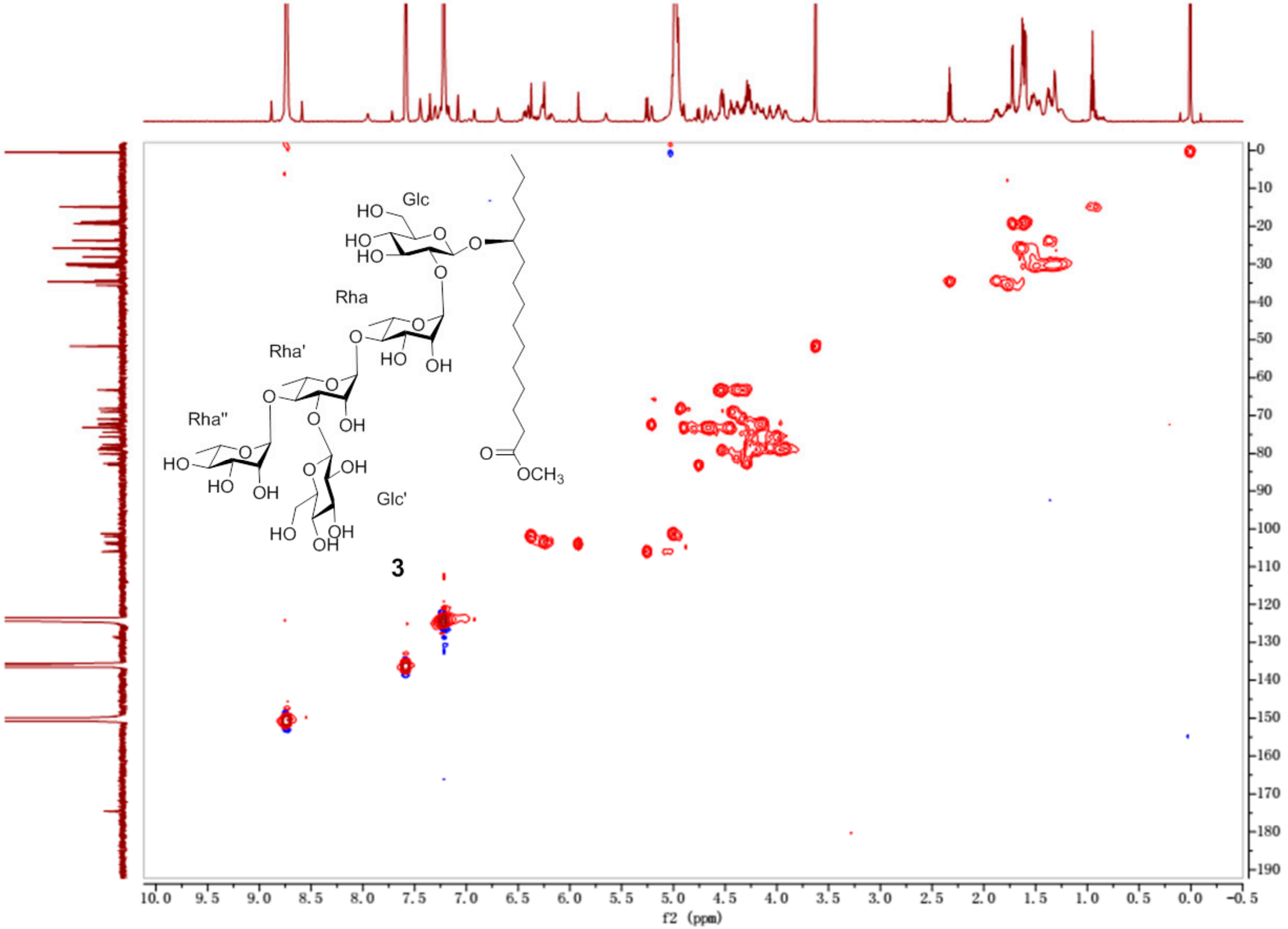

Figure S31. HSQC spectrum of compound **3** (600 MHz, pyridine-*d*5)

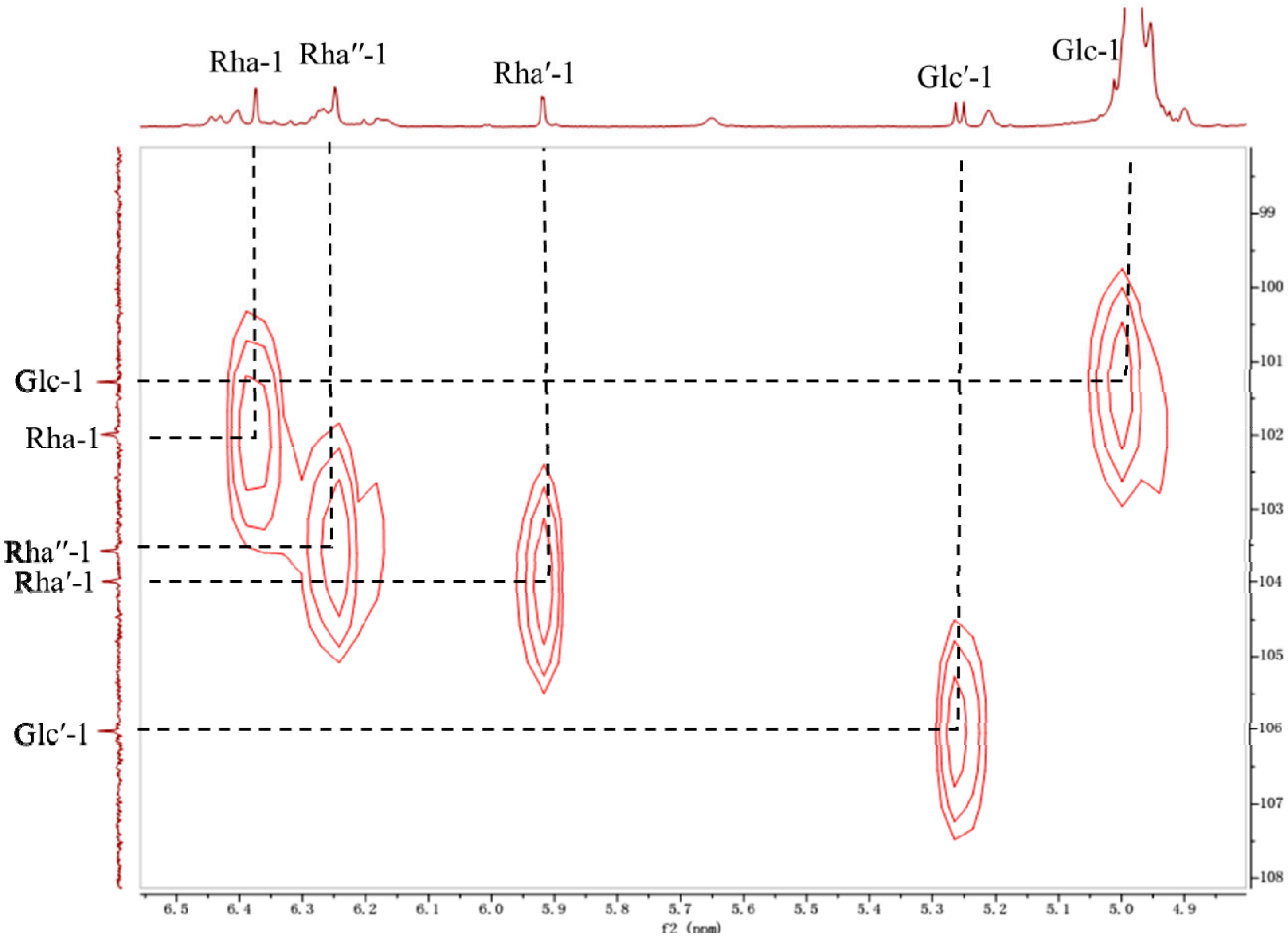

Figure S32. Expanded HSQC spectrum of compound **3** (600 MHz, pyridine-*d*5)

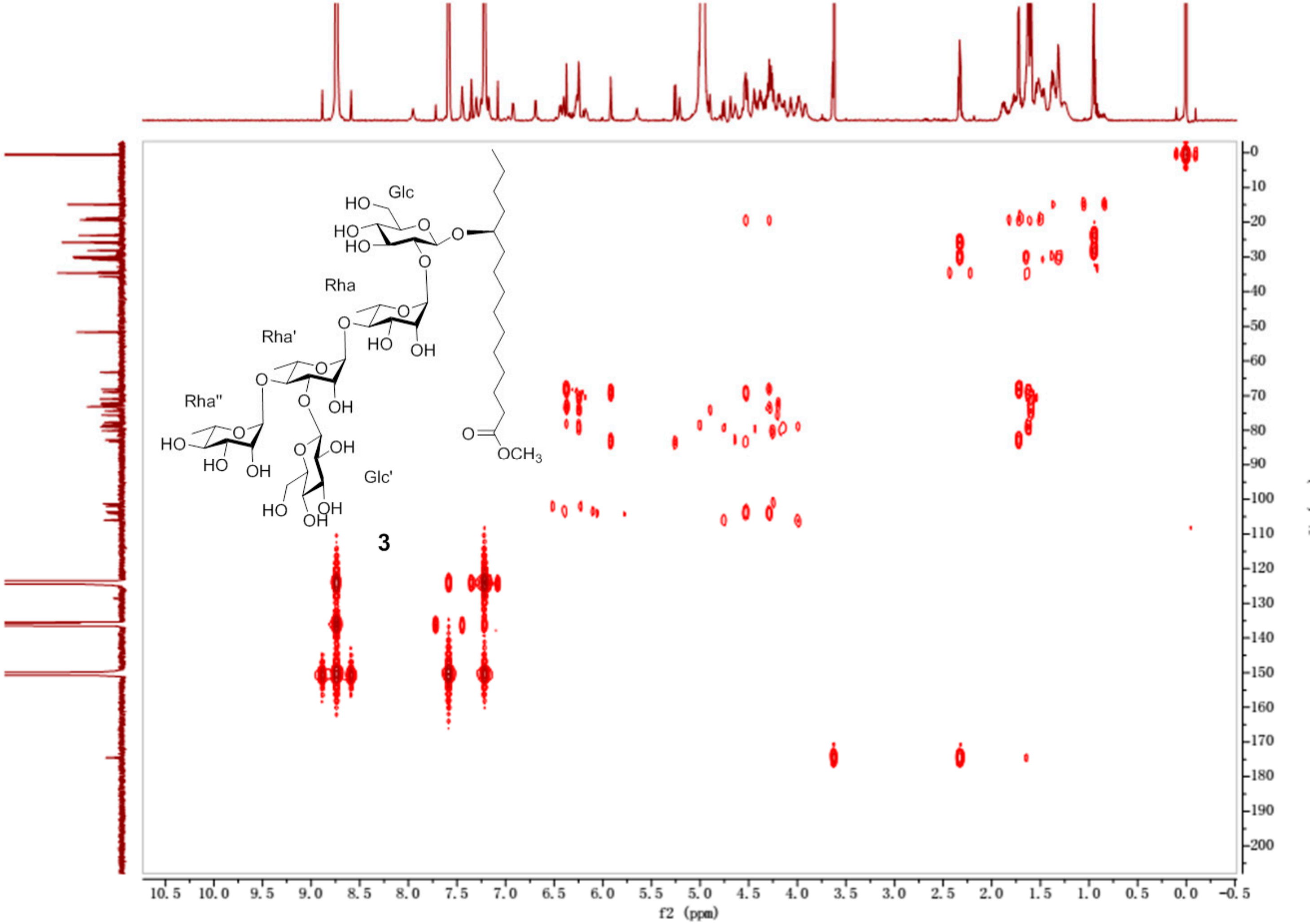

Figure S33. HMBC spectrum of compound **3** (600 MHz, pyridine-d_5)

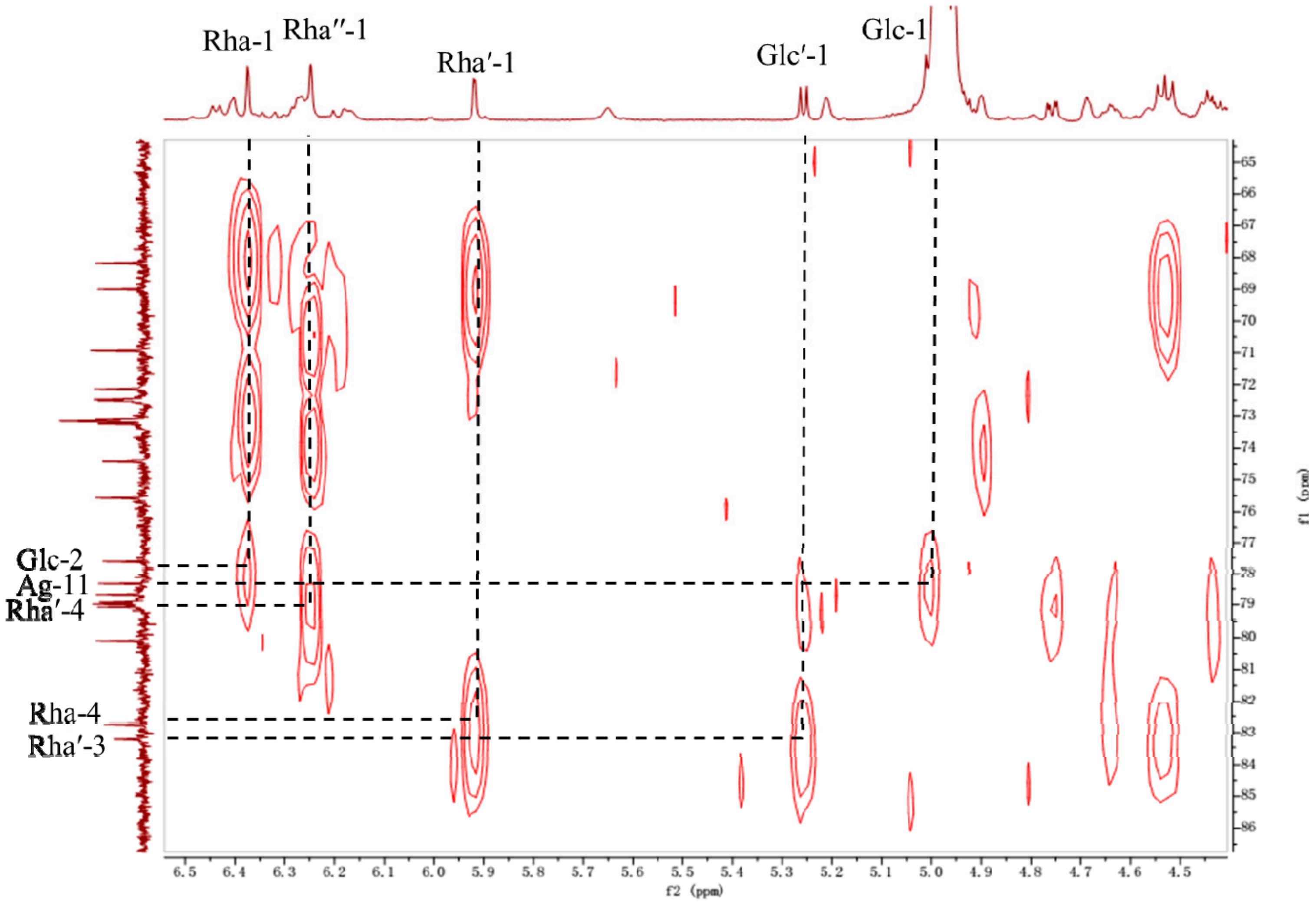

Figure S34. Expanded HMBC spectrum on the glycosidic linkages of compound **3** (600 MHz, pyridine-d_5)

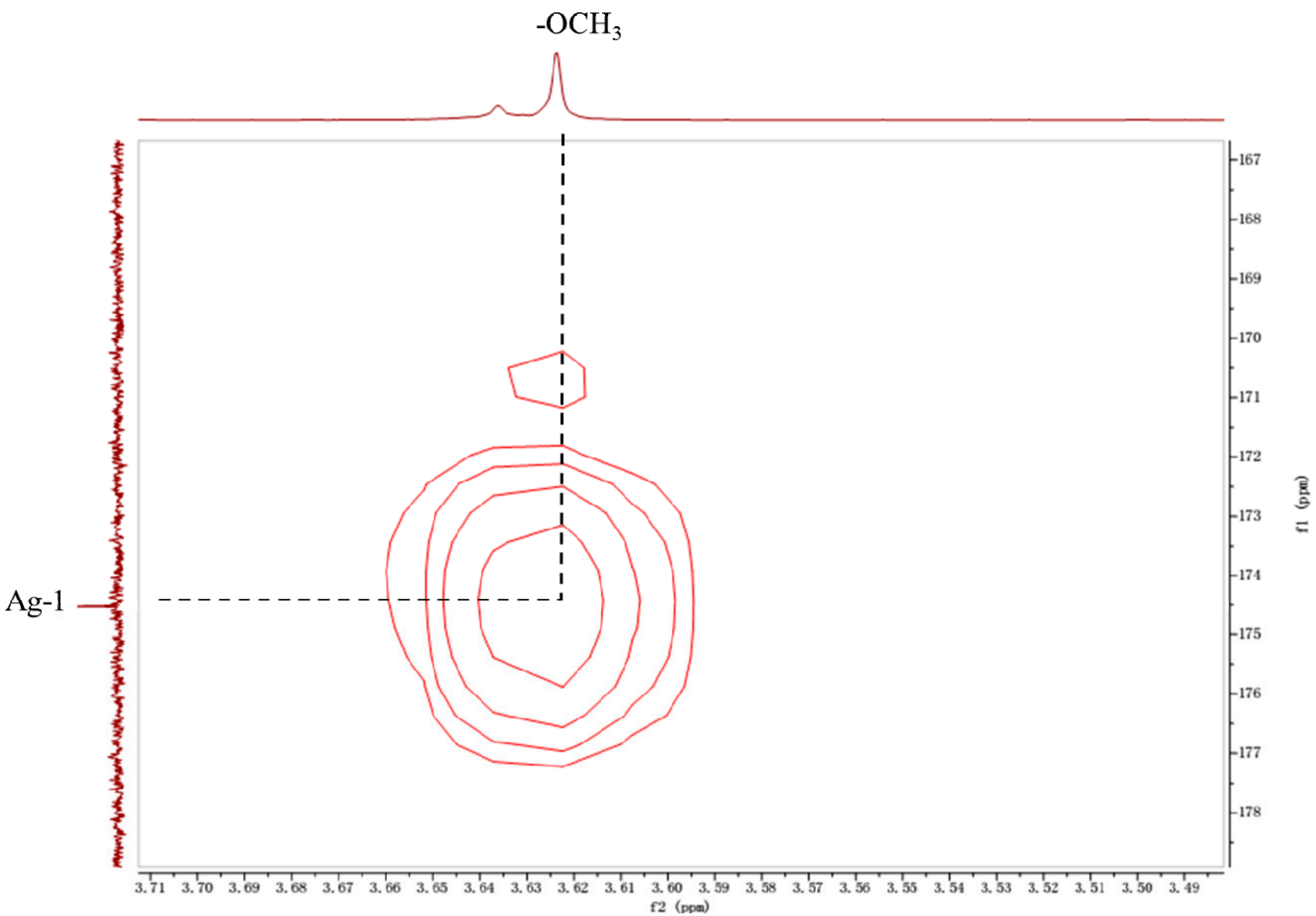

Figure S35. Expanded HMBC spectrum on the ester linkages of compound **3** (600 MHz, pyridine-*d*5)

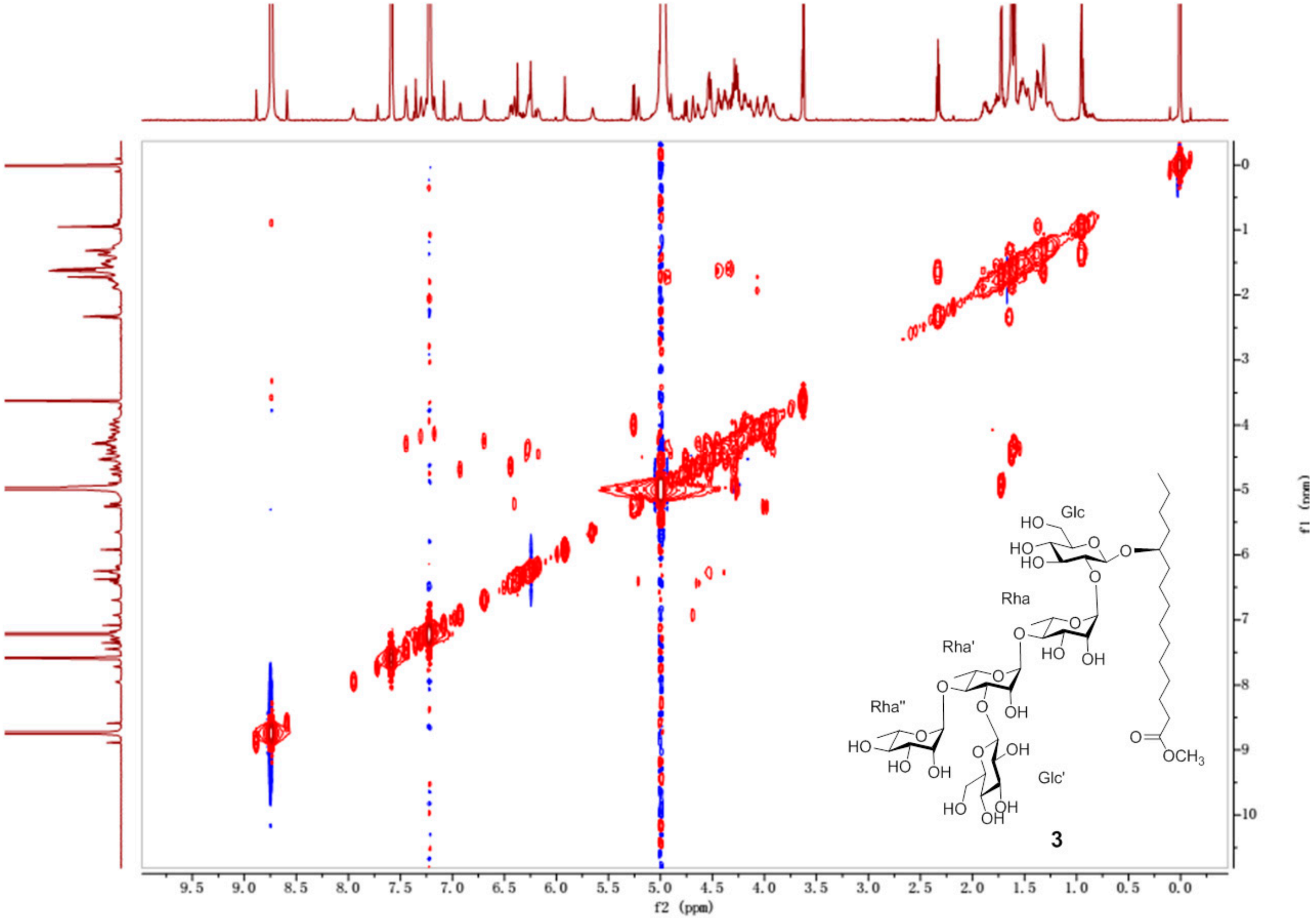

Figure S36. ^{1}H-^{1}H COSY spectrum of compound **3** (600 MHz, pyridine-d_5)

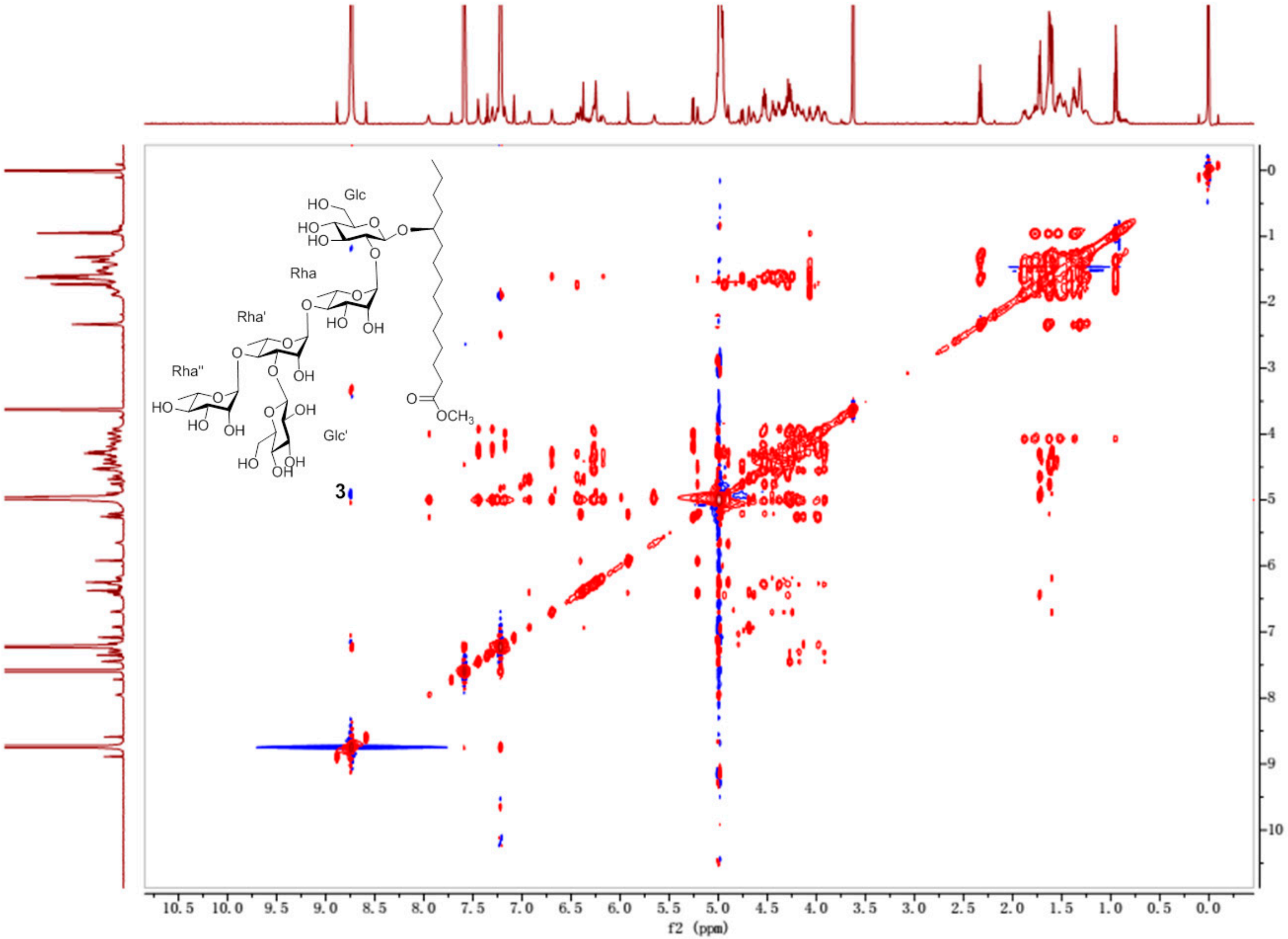

Figure S37. TOCSY spectrum of compound **3** (600 MHz, pyridine-d_5)

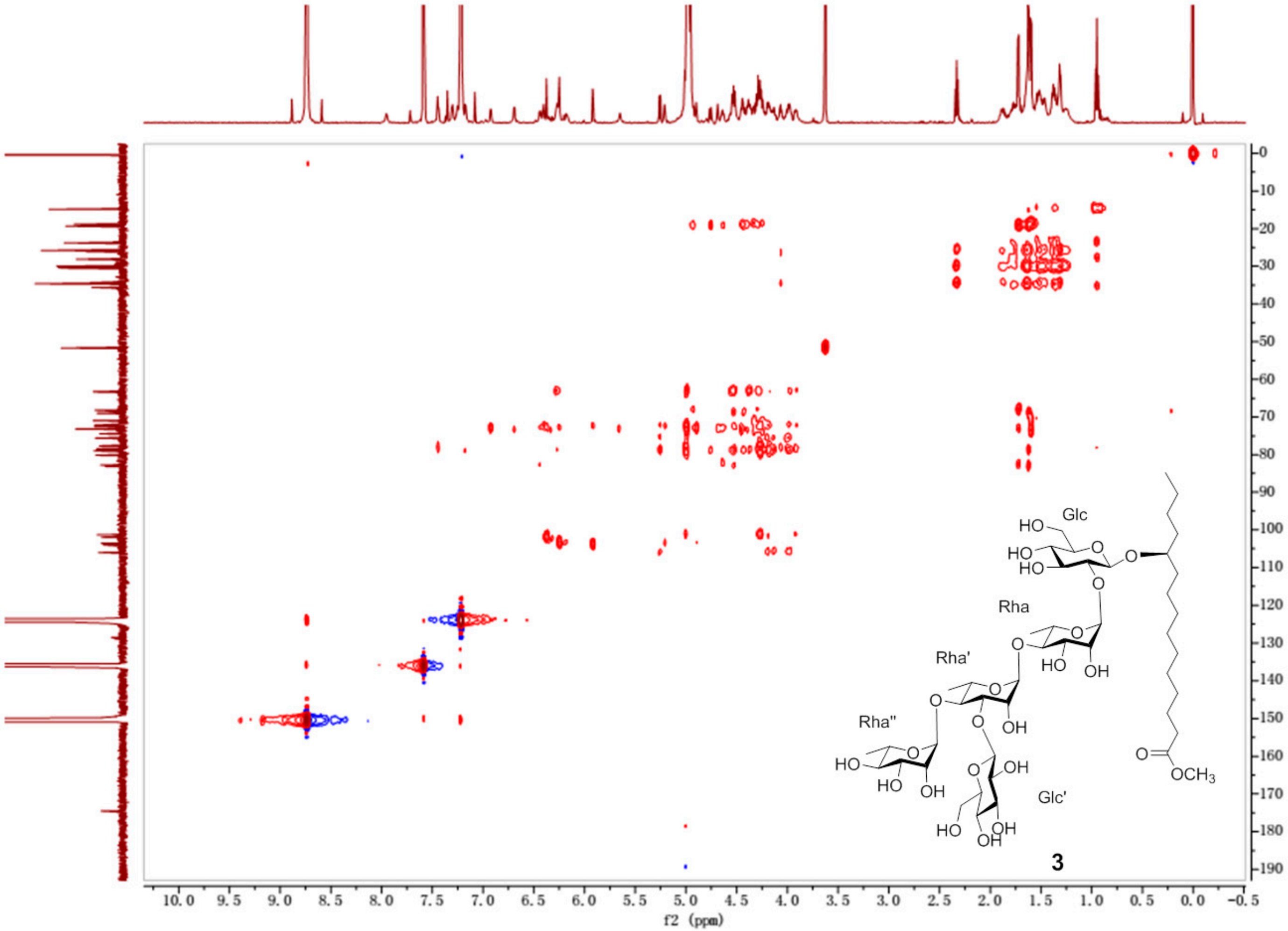

Figure S38. HSQC-TOCSY spectrum of compound **3** (600 MHz, pyridine-d_5)

Elemental Composition Report

Single Mass Analysis

Tolerance = 5.0 mDa / DBE: min = -1.5, max = 50.0
Element prediction: Off
Number of isotope peaks used for i-FIT = 3

Monoisotopic Mass, Even Electron Ions
10155 formula(e) evaluated with 1 results within limits (up to 50 closest results for each mass)
Elements Used:
C: 46-46 H: 82-82 N: 0-100 O: 0-100 Na: 0-4

4--P--N
240906-1-262-2-JDT-42 19 (0.136)

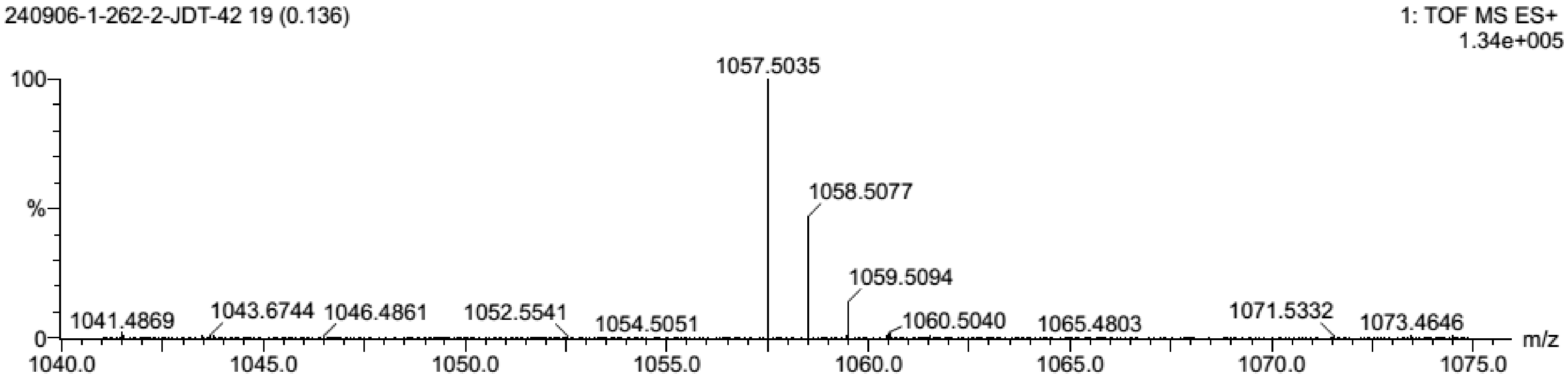

Minimum:				-1.5				
Maximum:		5.0	10.0	50.0				

Mass	Calc. Mass	mDa	PPM	DBE	i-FIT	Norm	Conf(%)	Formula
1057.5035	1057.5043	-0.8	-0.8	5.5	447.2	n/a	n/a	C46 H82 O25 Na

Figure S39. HRESIMS spectrum of compound **3**

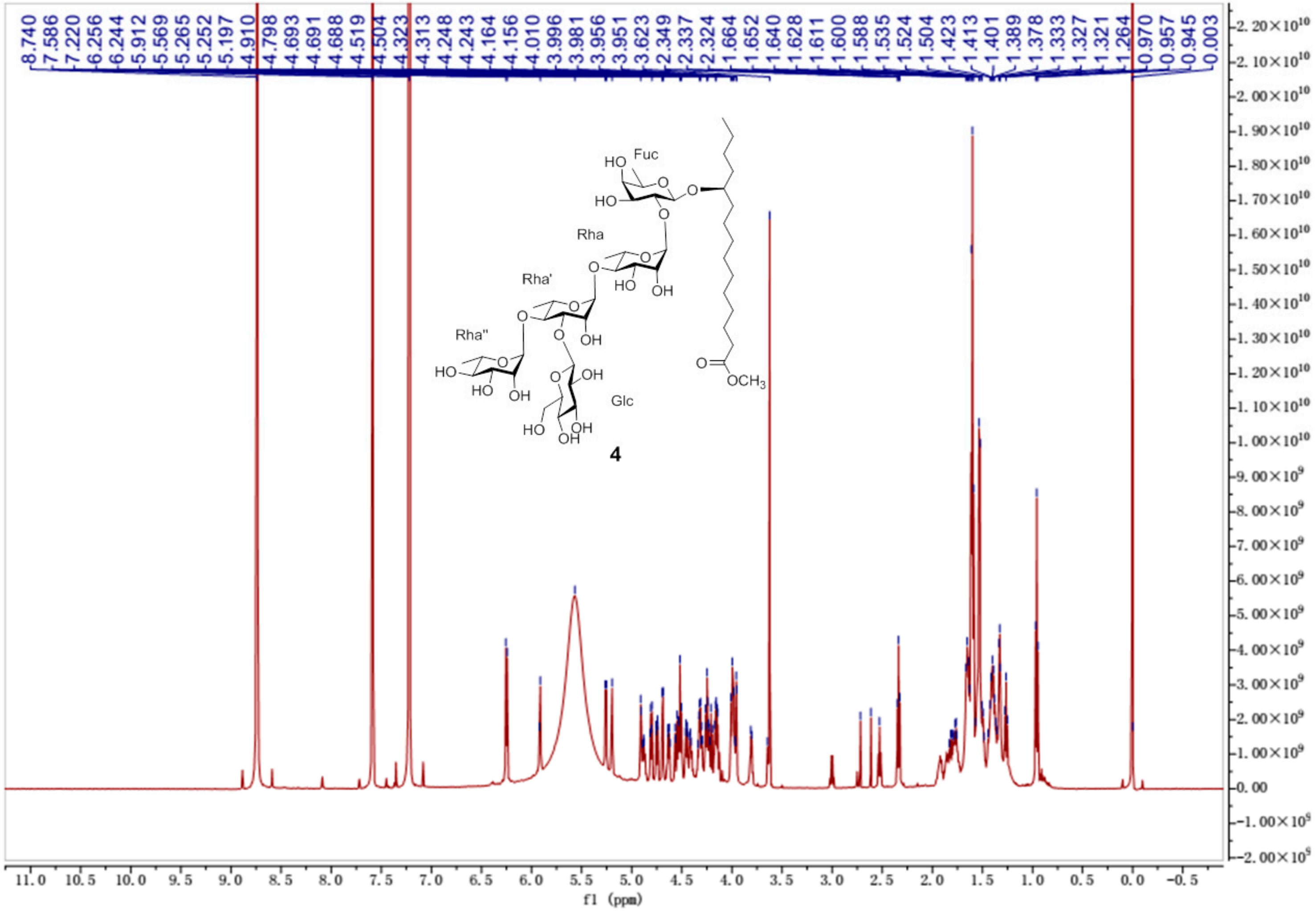

Figure S40. ^{1}H-NMR spectrum of compound **4** (600 MHz, pyridine-d_5)

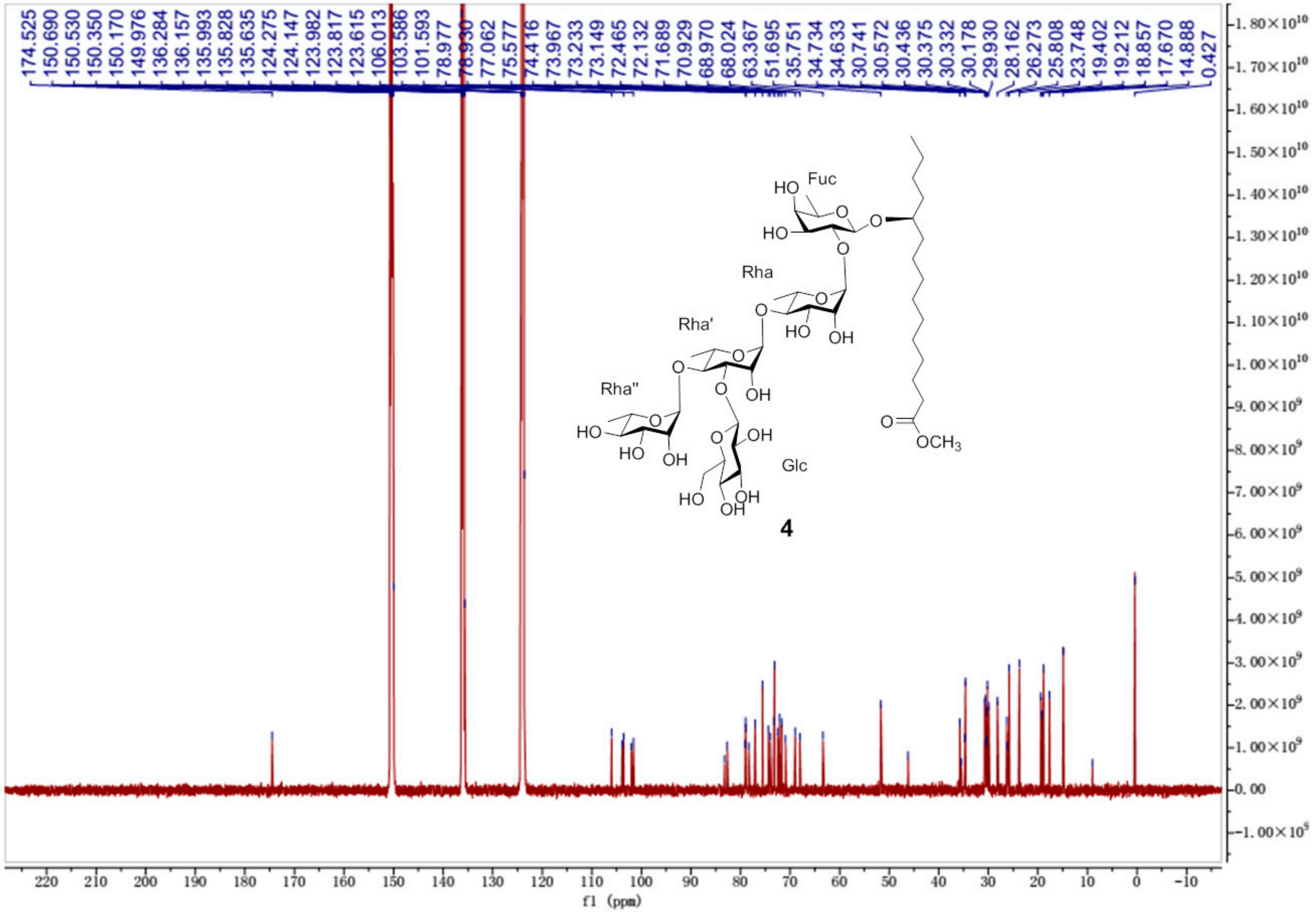

Figure S41. ^{13}C-NMR spectrum of compound **4** (151 MHz, pyridine-d_5)

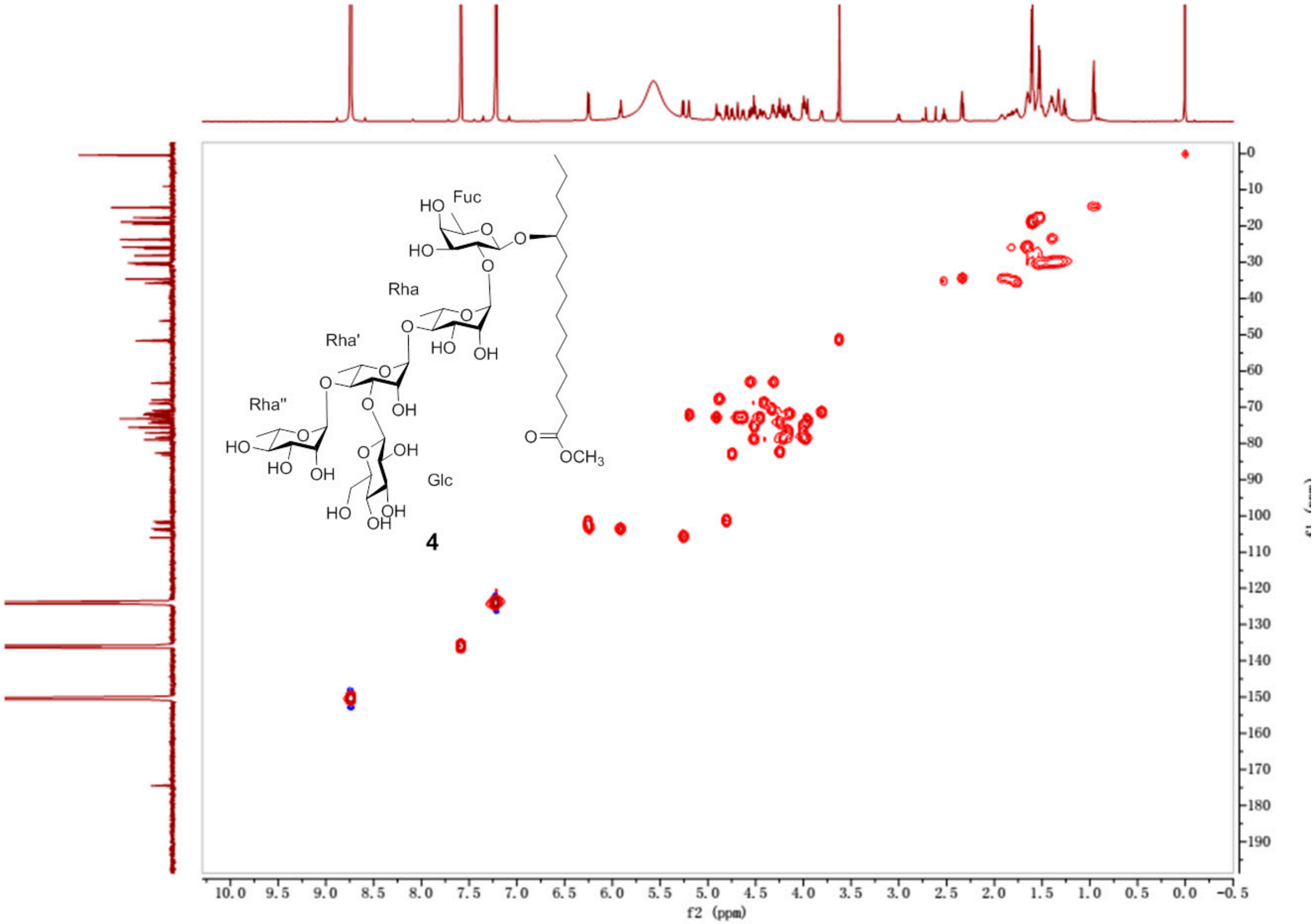

Figure S42. HSQC spectrum of compound **4** (600 MHz, pyridine-d_5)

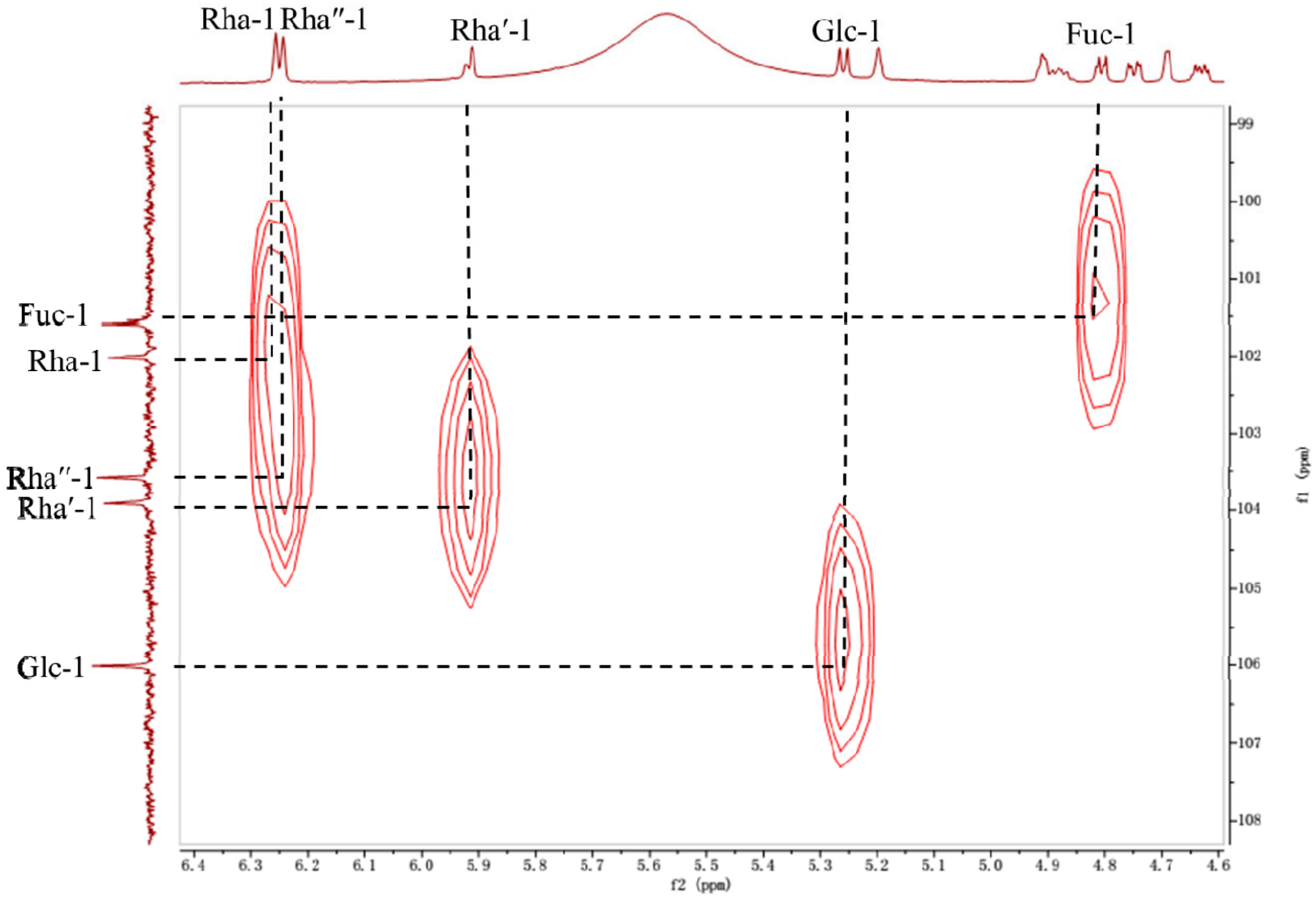

Figure S43. Expanded HSQC spectrum of compound **4** (600 MHz, pyridine-d_5)

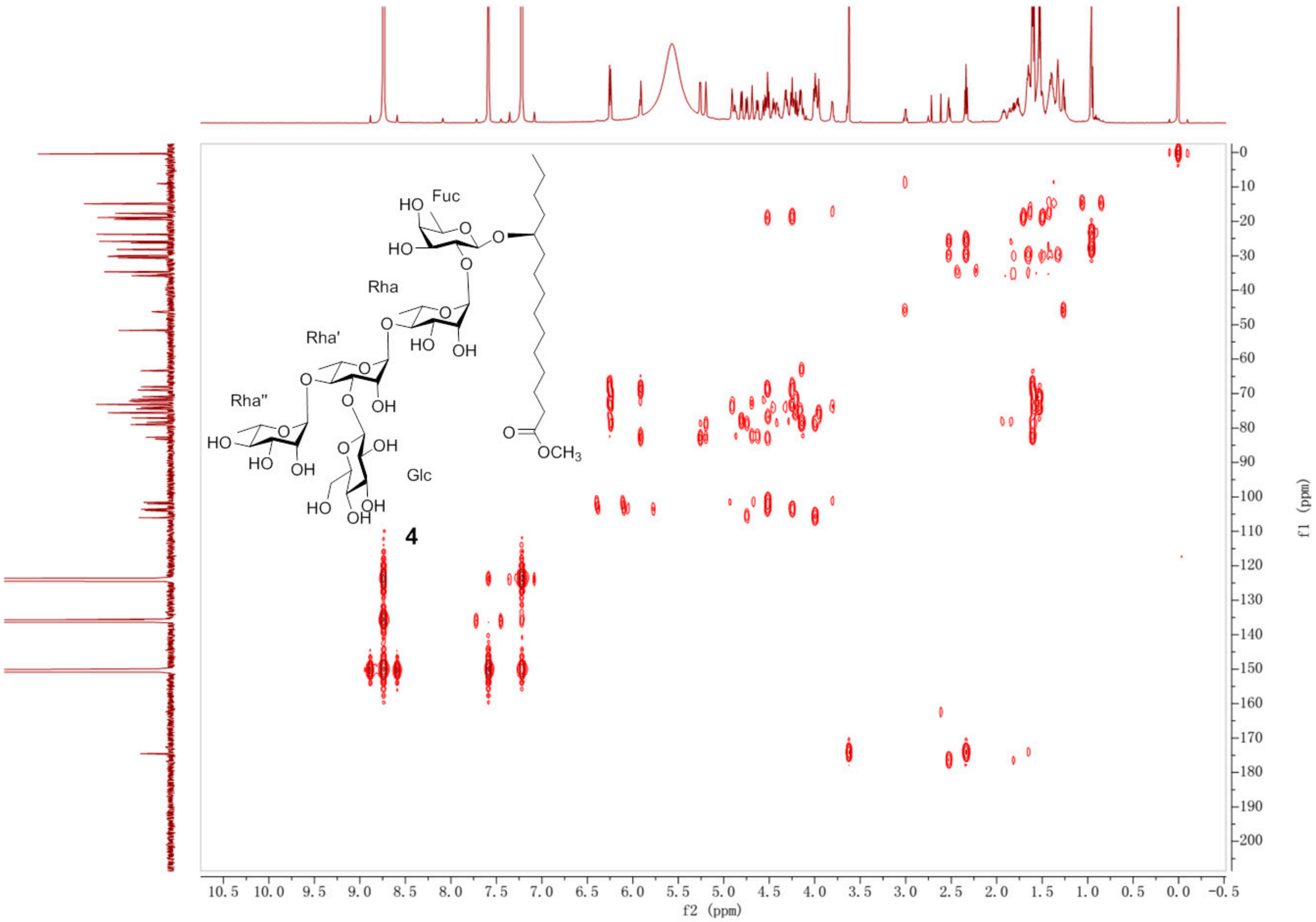

Figure S44. HMBC spectrum of compound **4** (600 MHz, pyridine-d_5)

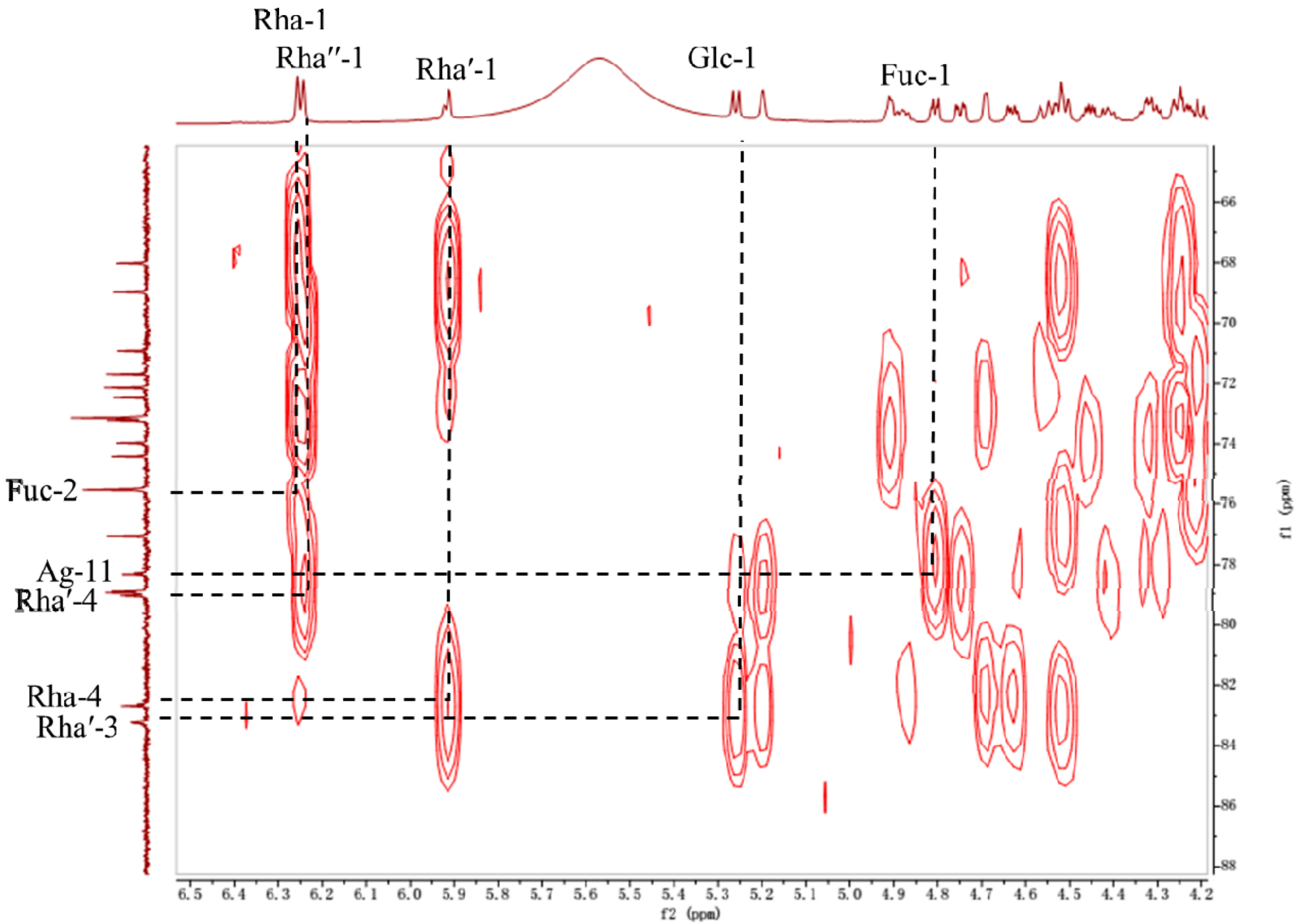

Figure S45. Expanded HMBC spectrum on the glycosidic linkages of compound **4** (600 MHz, pyridine-d_5)

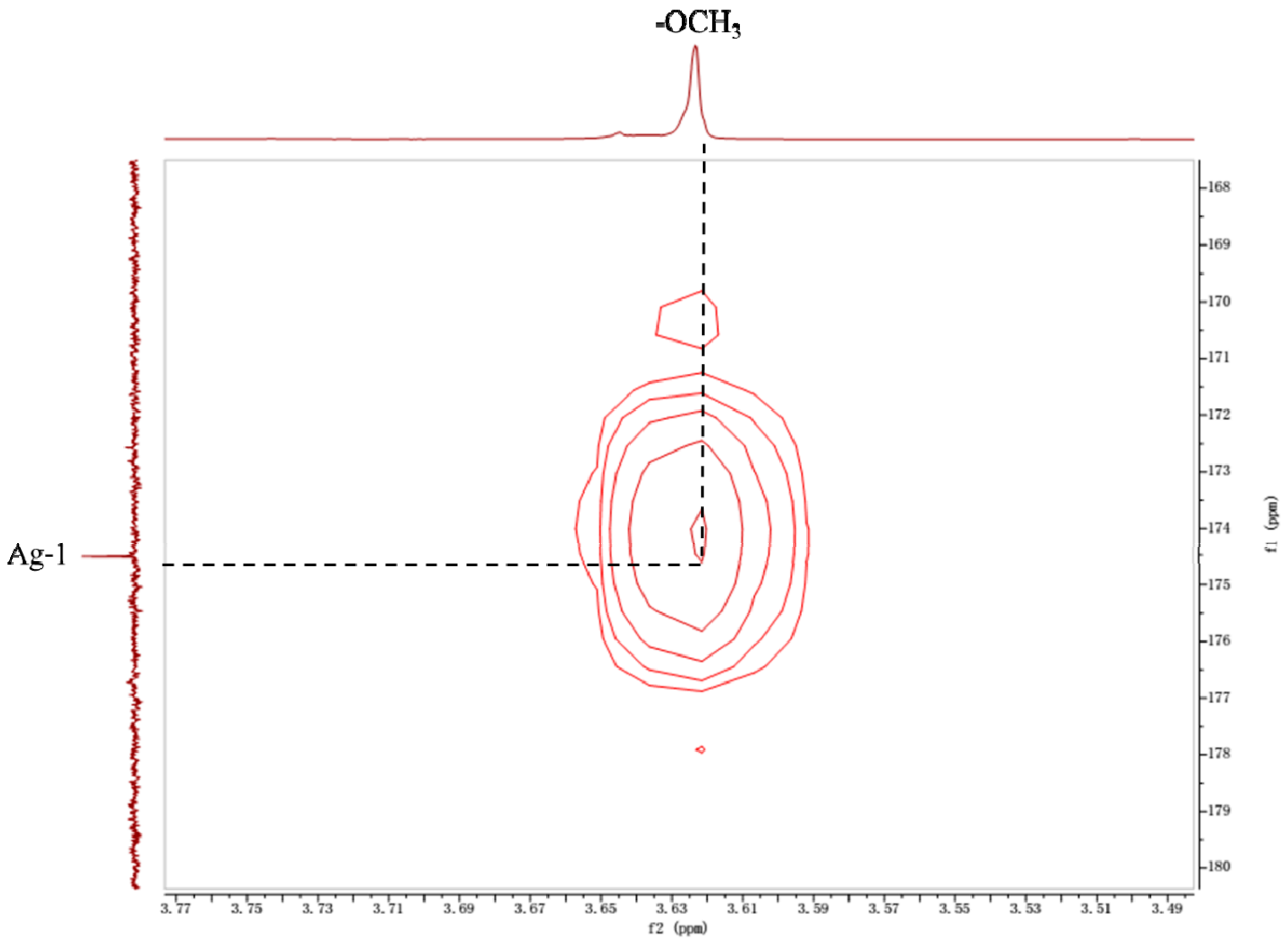

Figure S46. Expanded HMBC spectrum on the ester linkages of compound **4** (600 MHz, pyridine-*d*5)

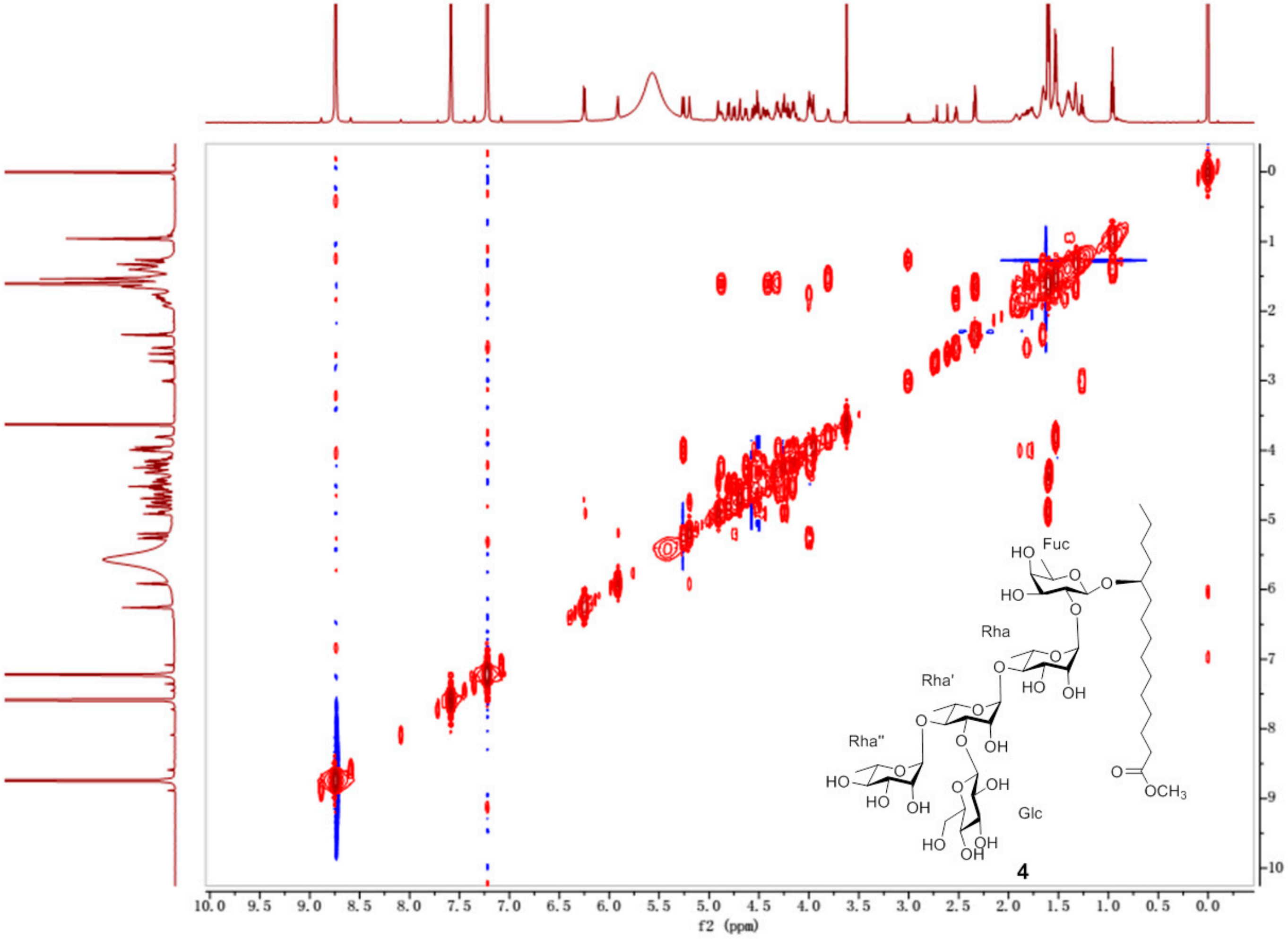

Figure S47. ^{1}H-^{1}H COSY spectrum of compound **4** (600 MHz, pyridine-d_5)

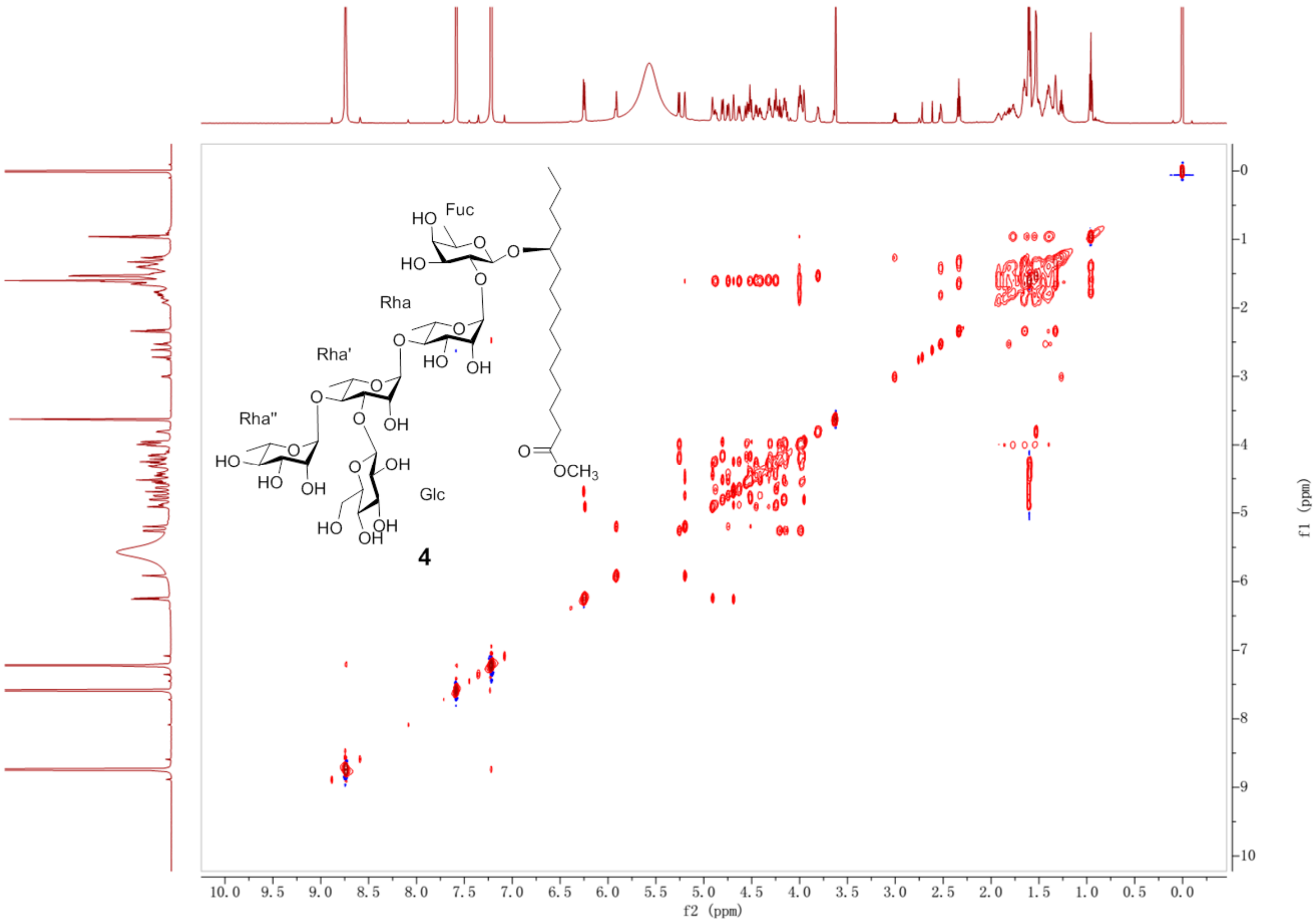

Figure S48. TOCSY spectrum of compound **4** (600 MHz, pyridine-d_5)

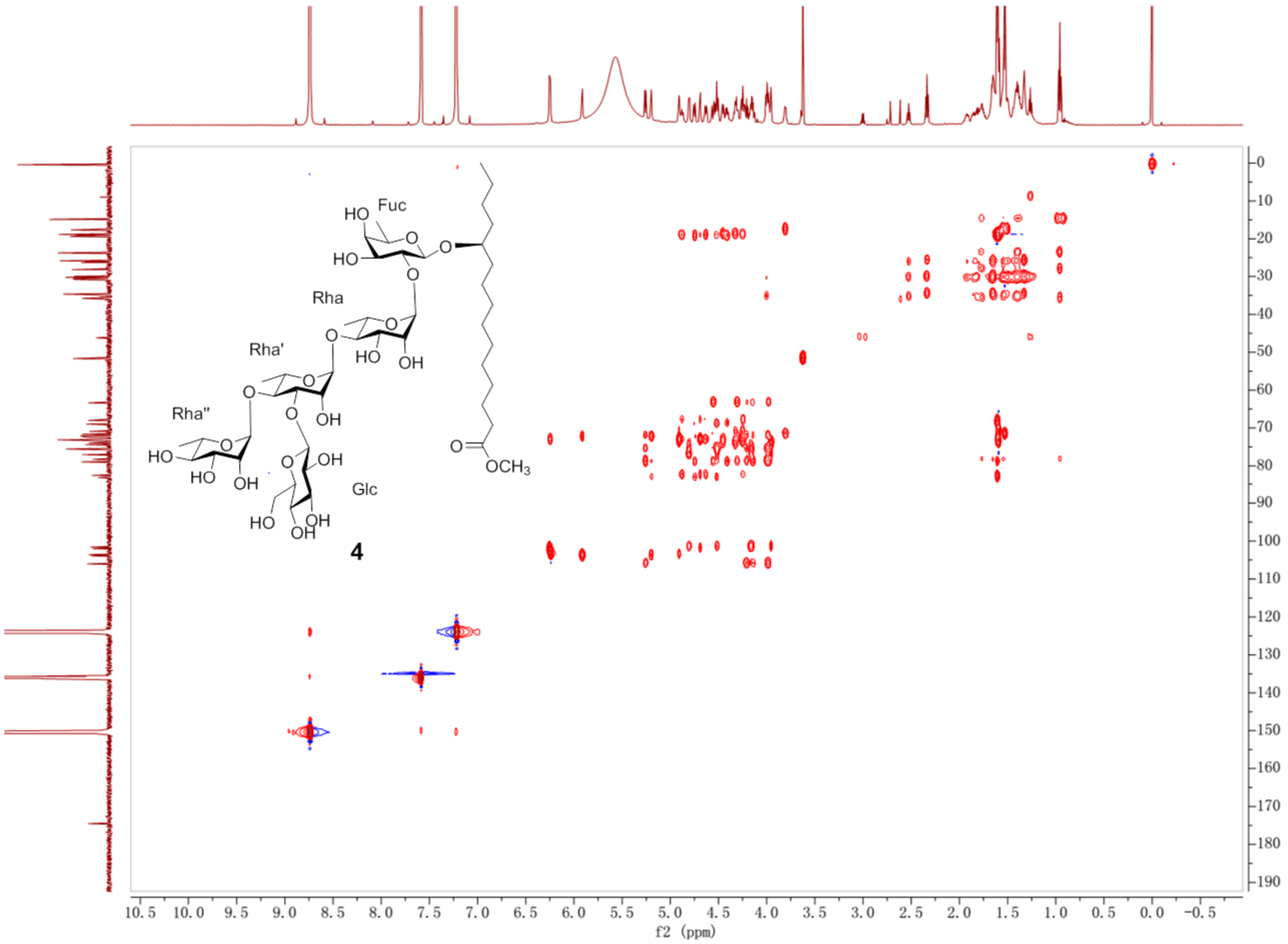

Figure S49. HSQC-TOCSY spectrum of compound **4** (600 MHz, pyridine-d_5)

Elemental Composition Report

Single Mass Analysis

Tolerance = 5.0 mDa / DBE: min = -1.5, max = 50.0
Element prediction: Off
Number of isotope peaks used for i-FIT = 3

Monoisotopic Mass, Even Electron Ions
6166 formula(e) evaluated with 1 results within limits (up to 50 closest results for each mass)
Elements Used:
C: 46-46 H: 82-82 N: 0-100 O: 0-100 Na: 0-2

1--P--N
240810-8-250-2-JDT-38 8 (0.079)

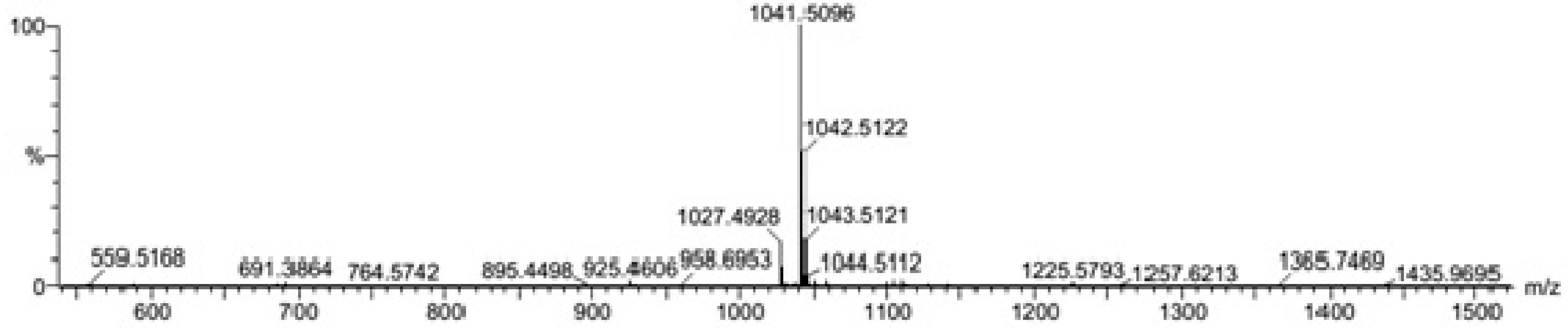

Minimum:				-1.5				
Maximum:		5.0	10.0	50.0				
Mass	Calc. Mass	mDa	PPM	DBE	i-FIT	Norm	Conf(%)	Formula
1041.5096	1041.5094	0.2	0.2	5.5	582.7	n/a	n/a	C46 H82 O24 Na

Figure S50. HRESIMS spectrum of compound **4**

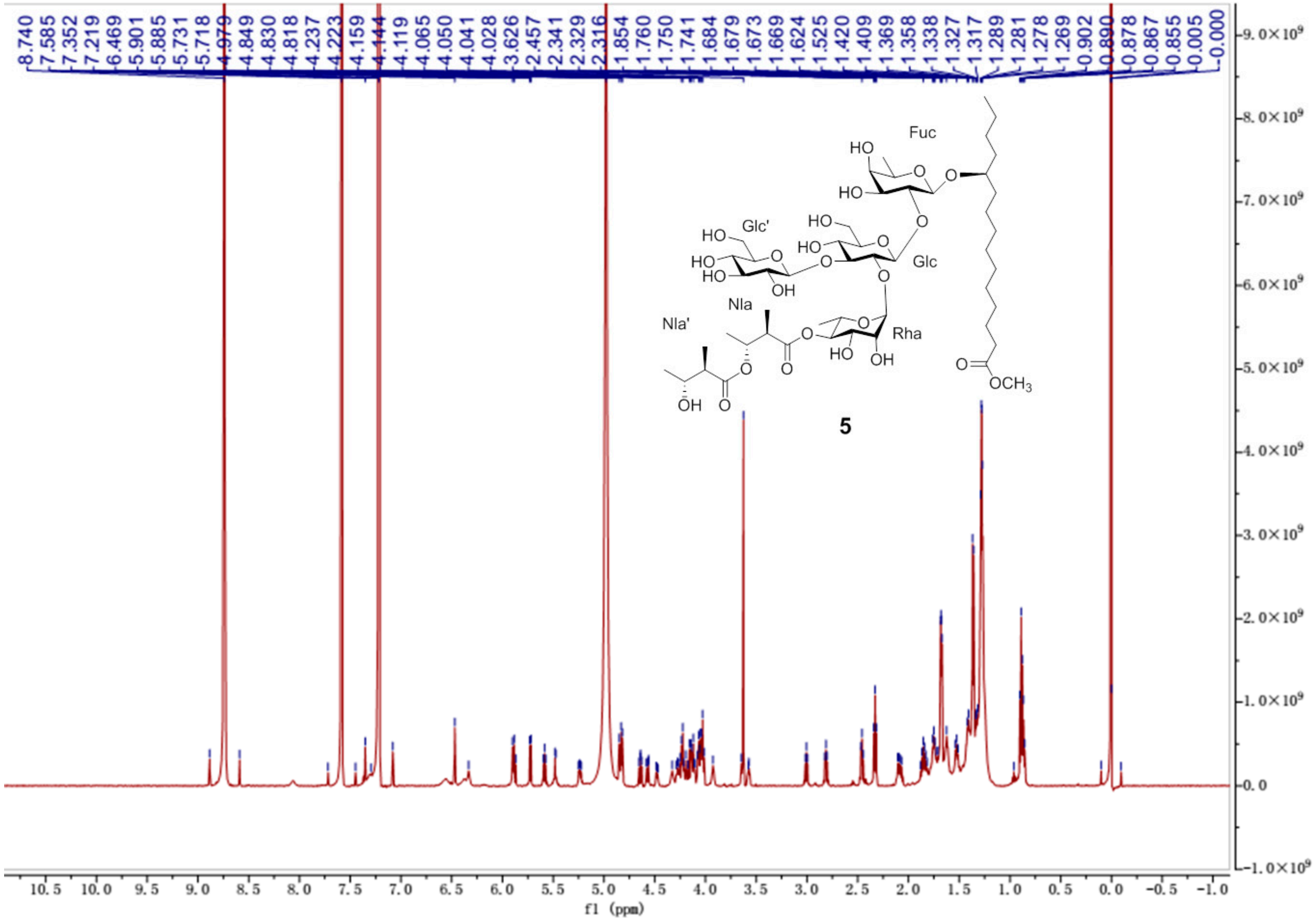

Figure S51. ^{1}H-NMR spectrum of compound **5** (600 MHz, pyridine-d_5)

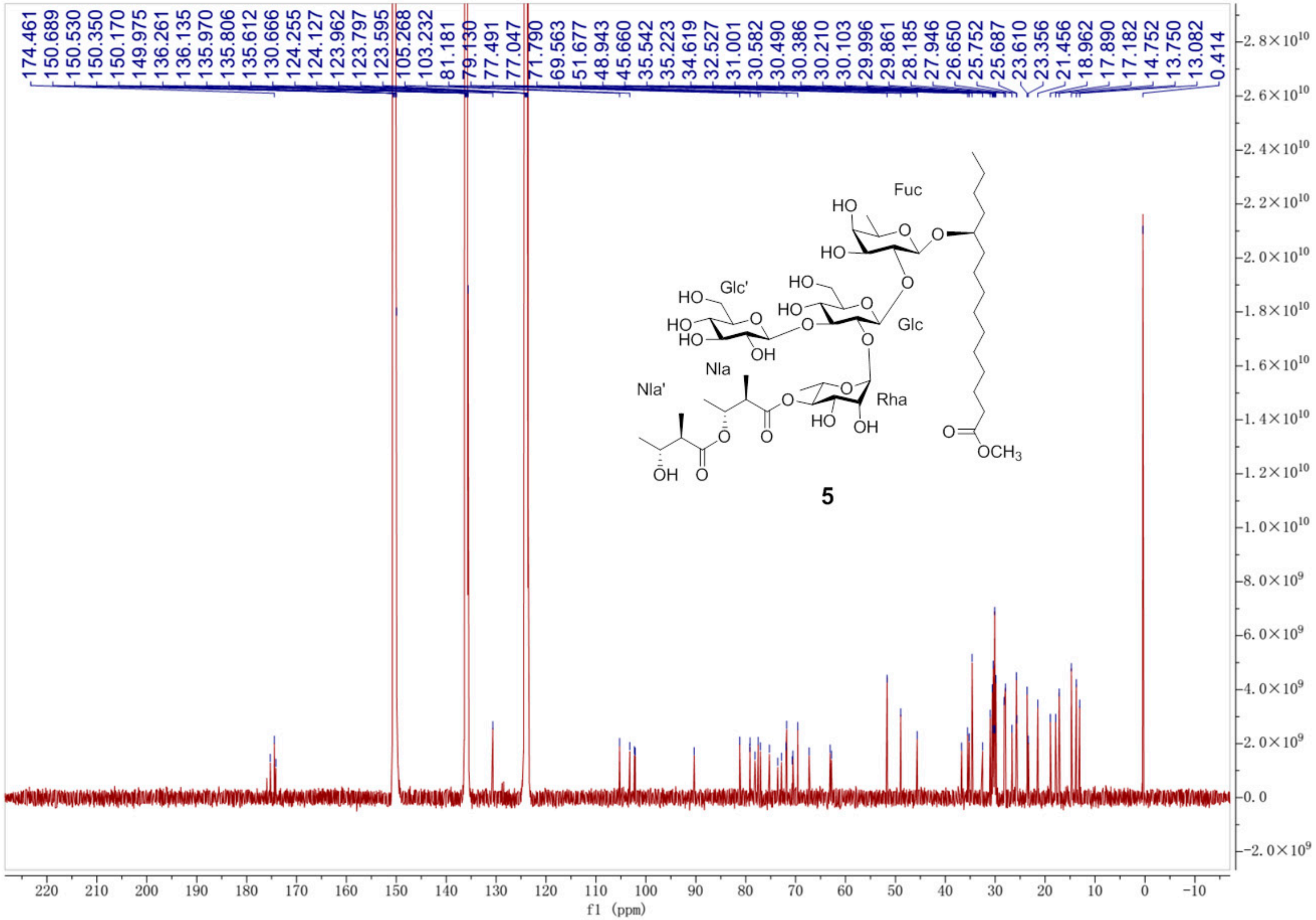

Figure S52. ^{13}C-NMR spectrum of compound **5** (151 MHz, pyridine-d_5)

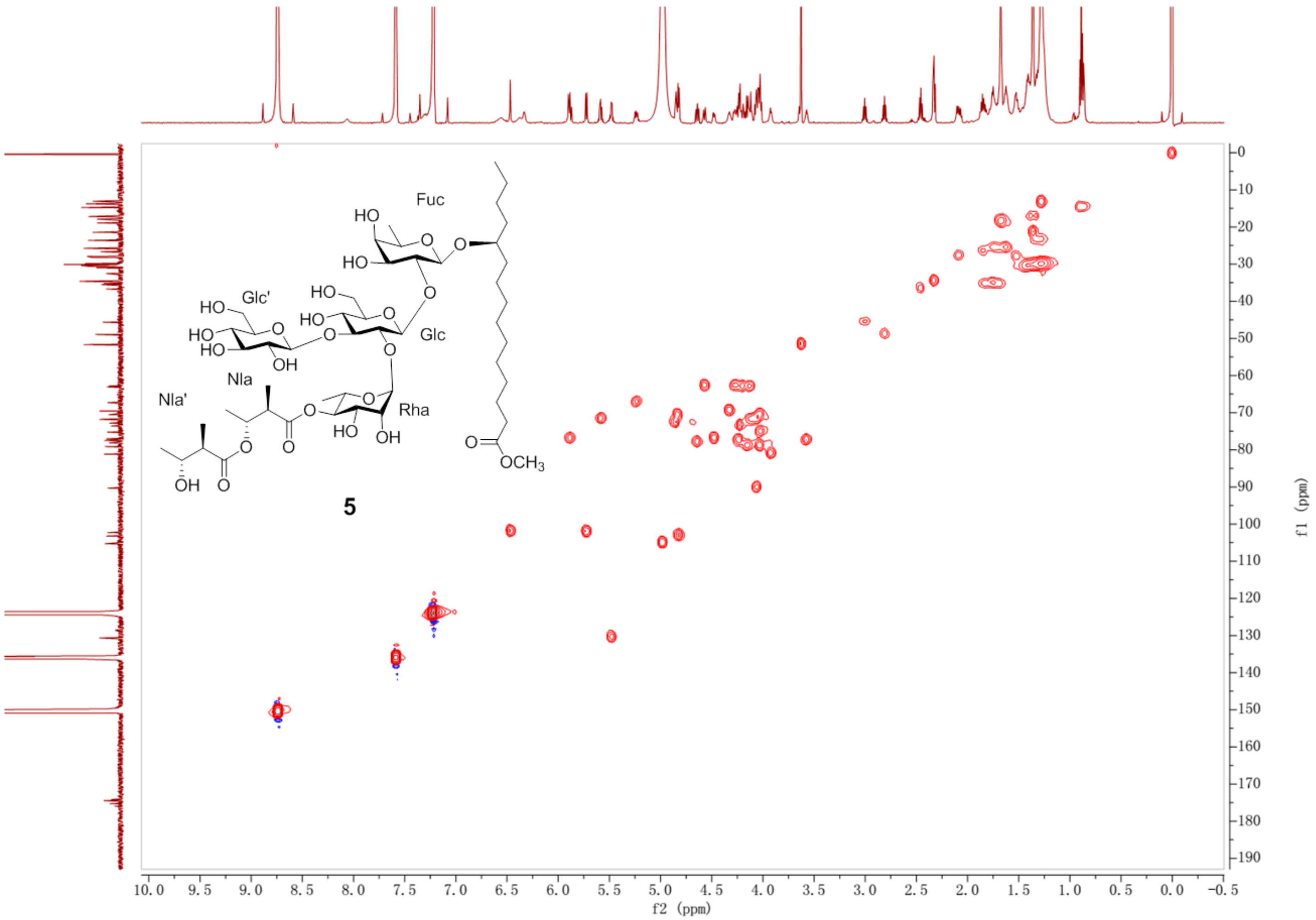

Figure S53. HSQC spectrum of compound **5** (600 MHz, pyridine-d_5)

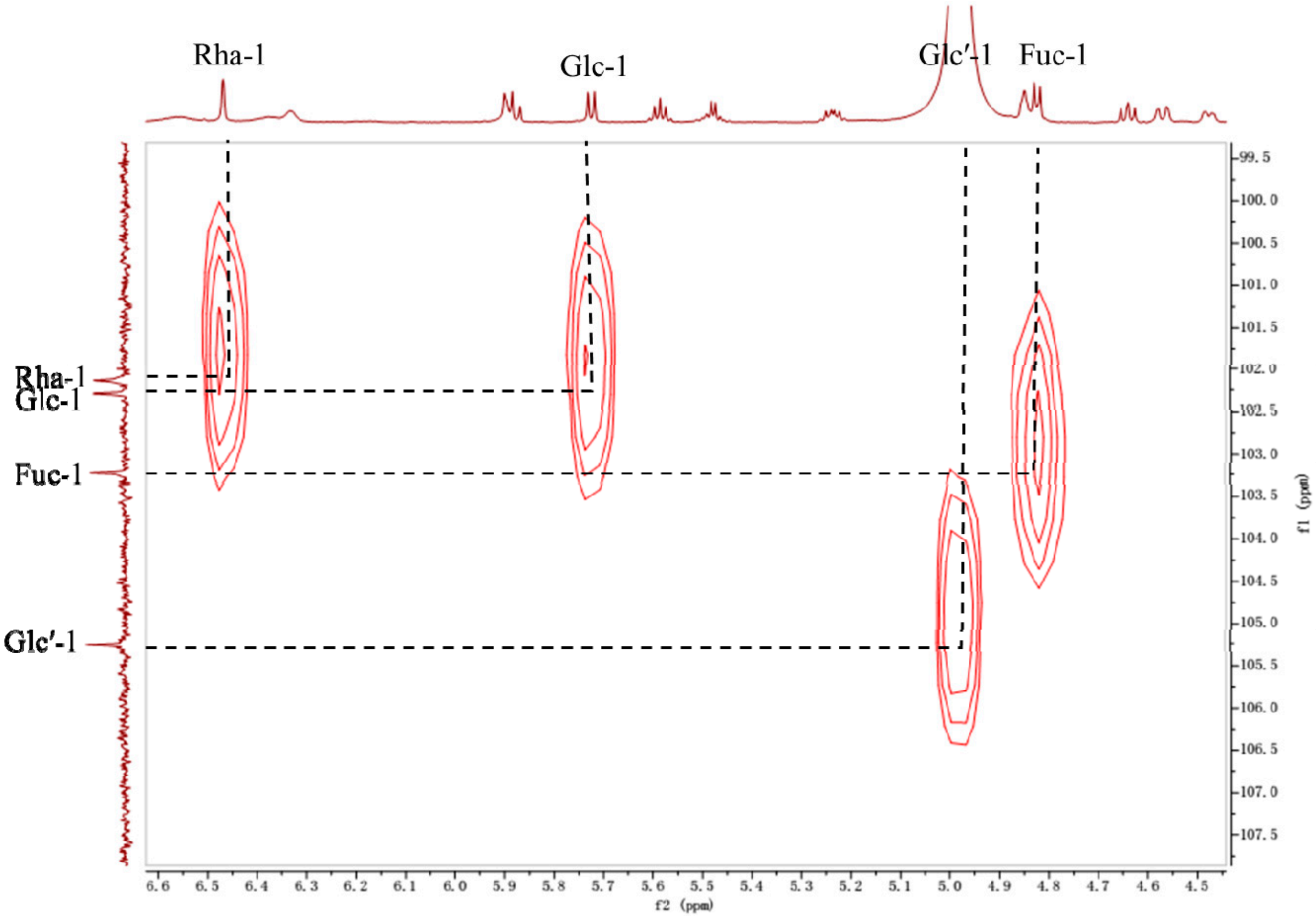

Figure S54. Expanded HSQC spectrum of compound **5** (600 MHz, pyridine-d_5)

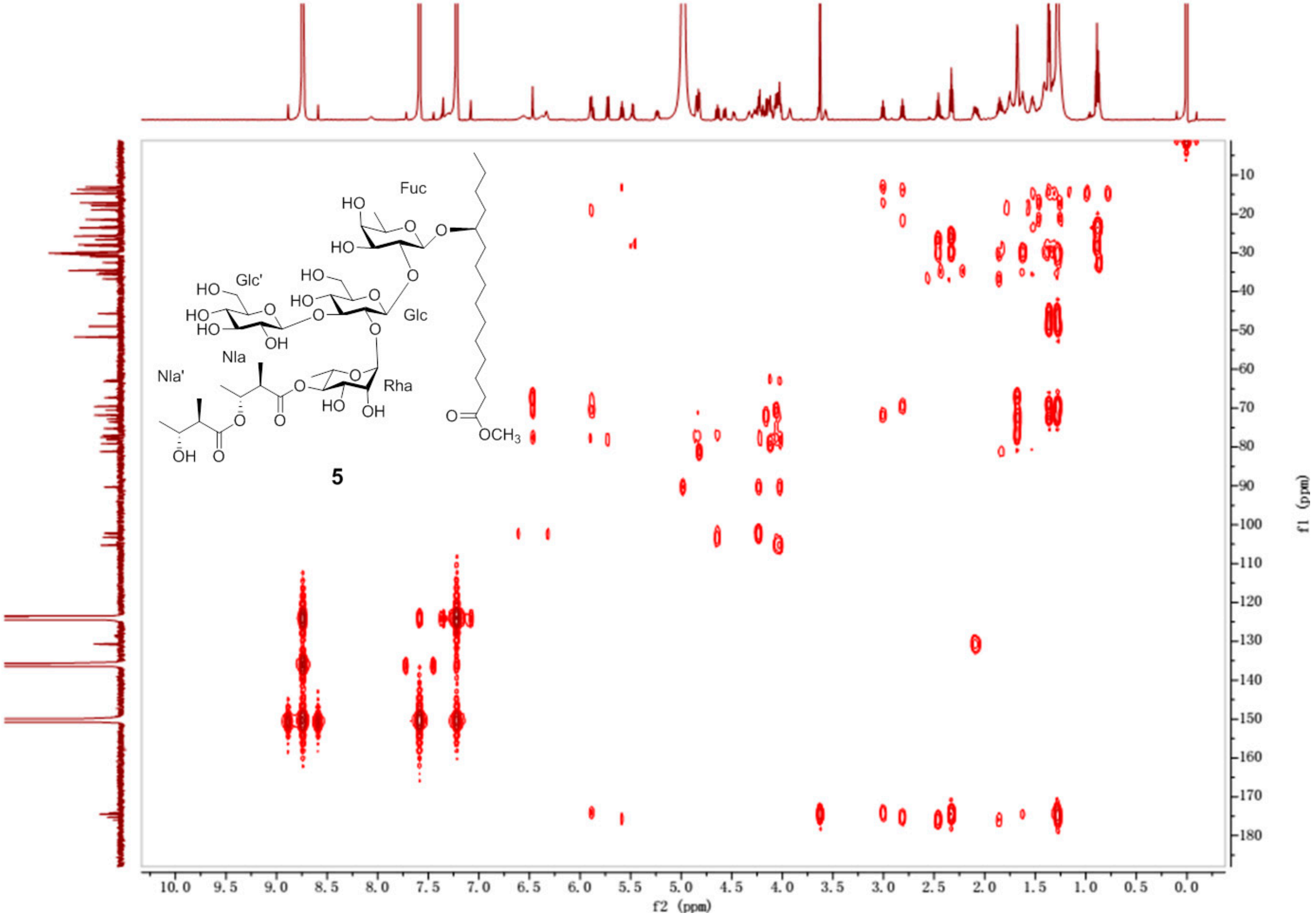

Figure S55. HMBC spectrum of compound **5** (600 MHz, pyridine-d_5)

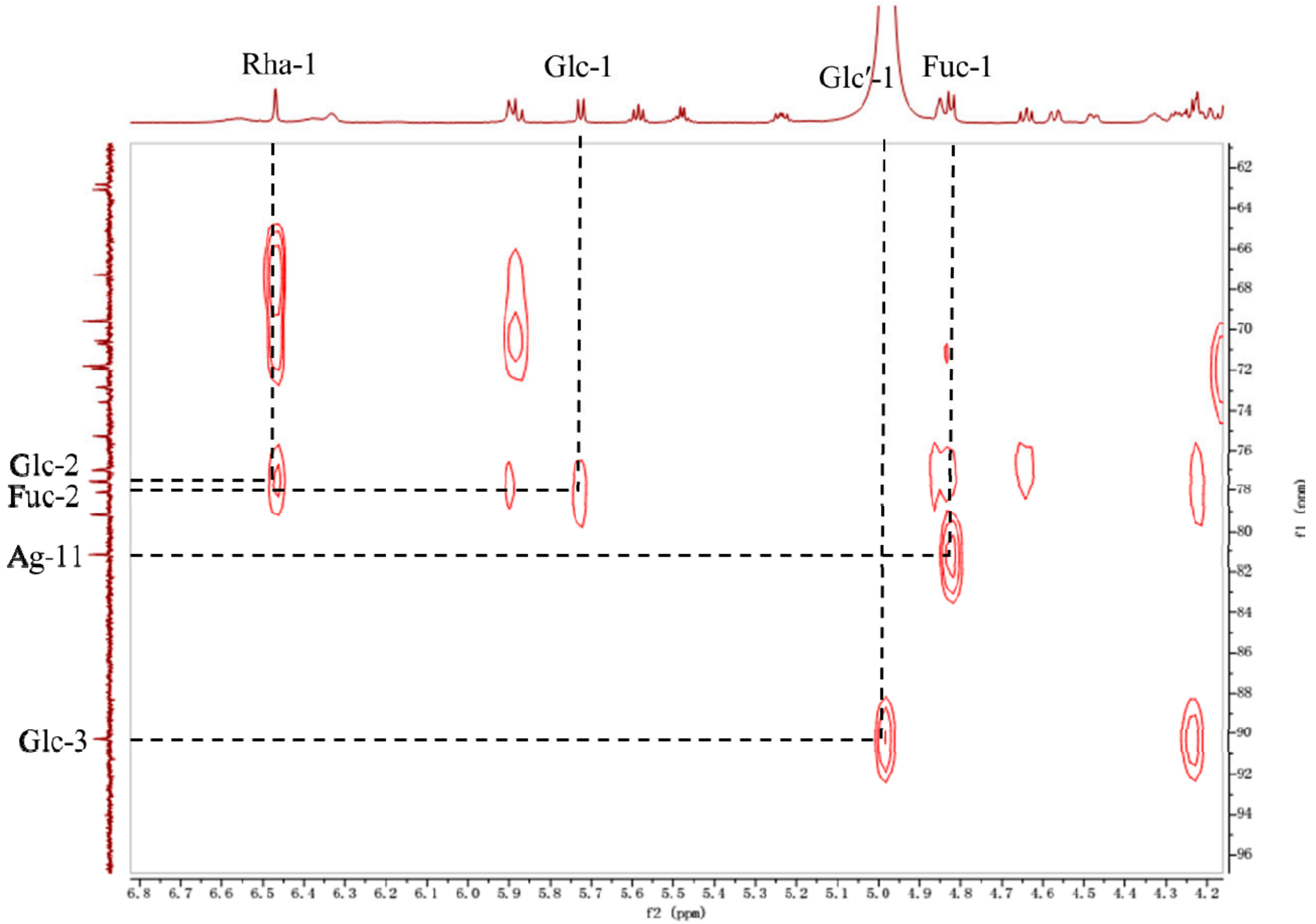

Figure S56. Expanded HMBC spectrum on the glycosidic linkages of compound **5** (600 MHz, pyridine-d_5)

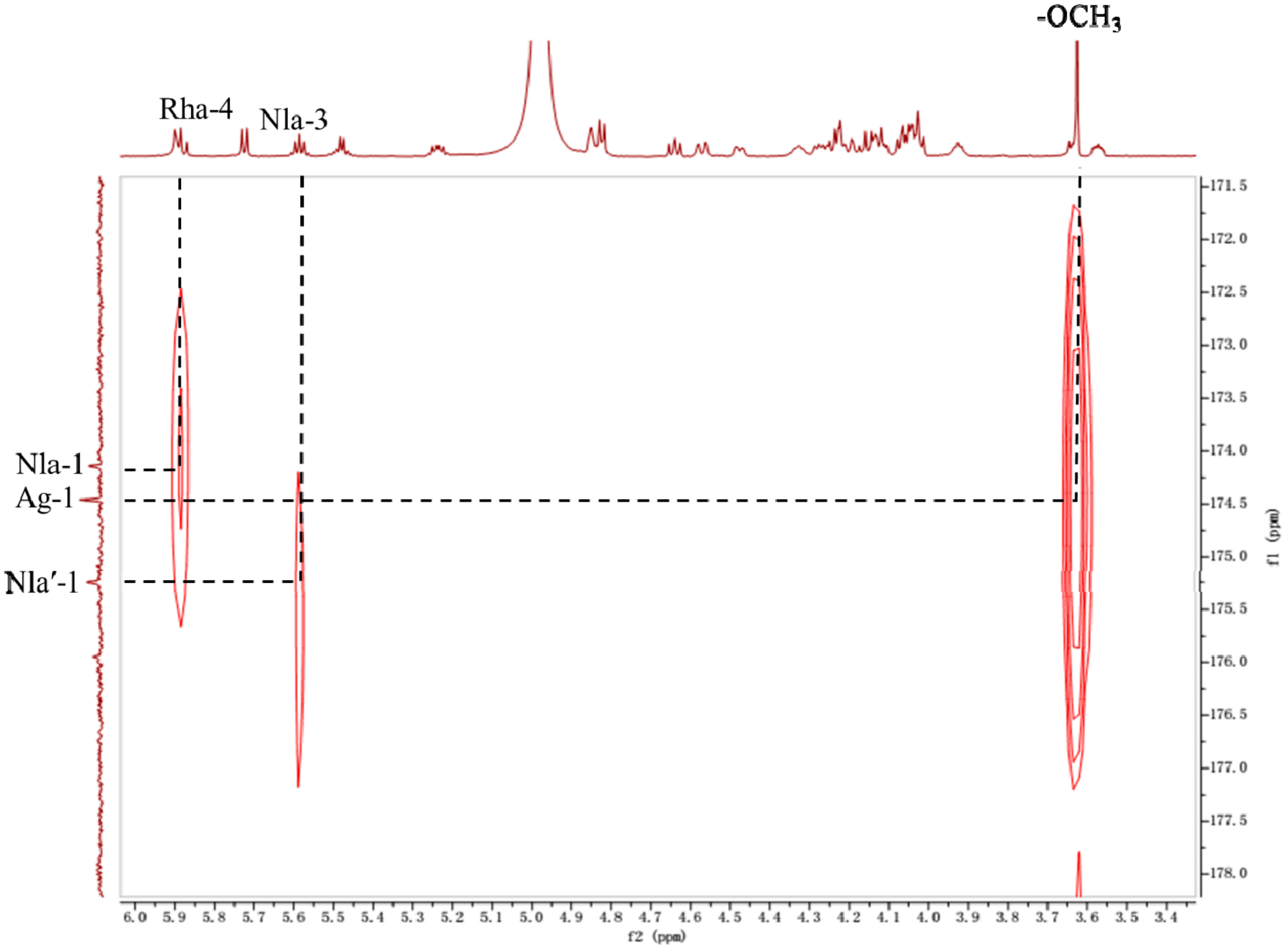

Figure S57. Expanded HMBC spectrum on the ester linkages of compound **5** (600 MHz, pyridine-*d*5)

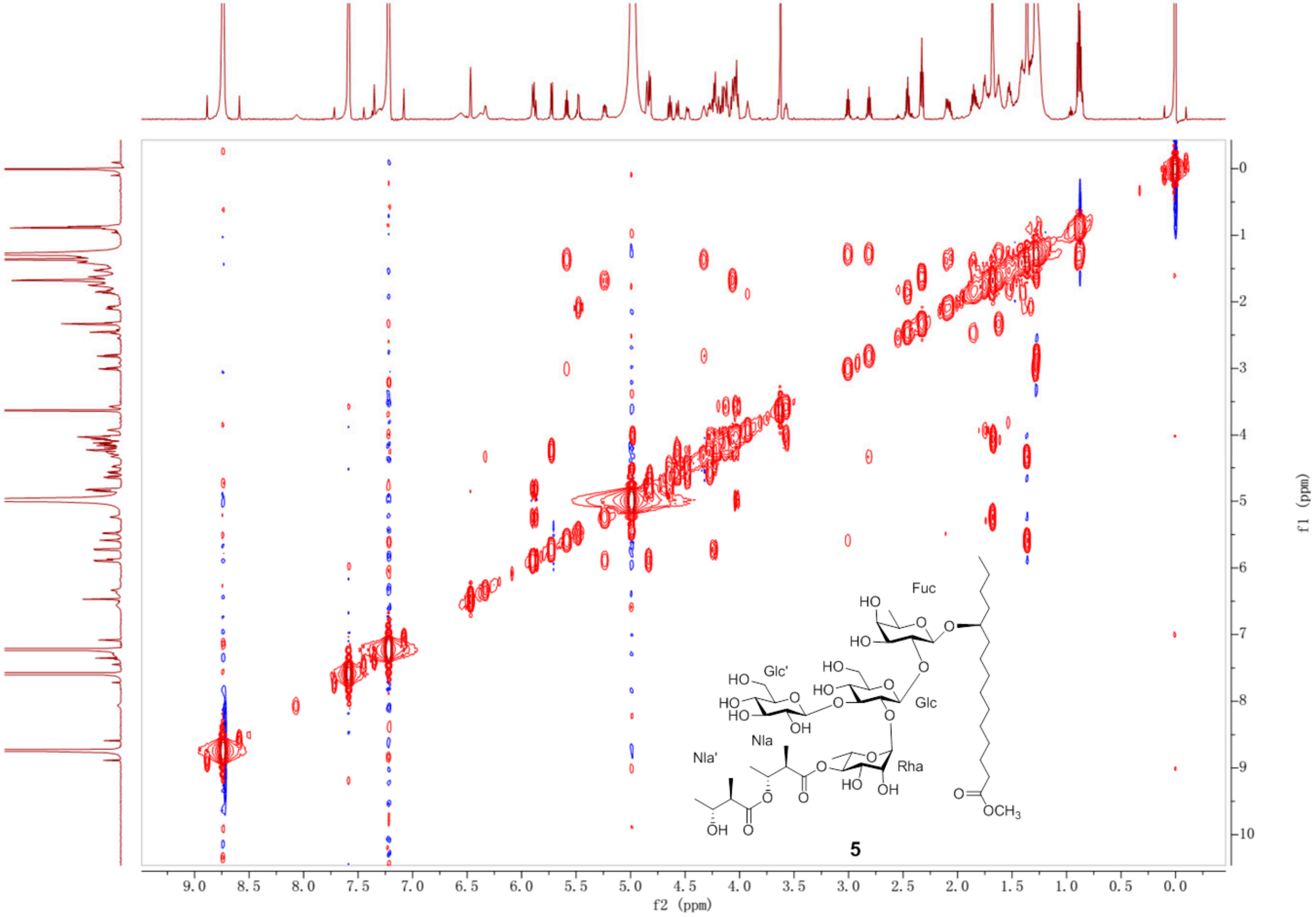

Figure S58. ^{1}H-^{1}H COSY spectrum of compound **5** (600 MHz, pyridine-d_5)

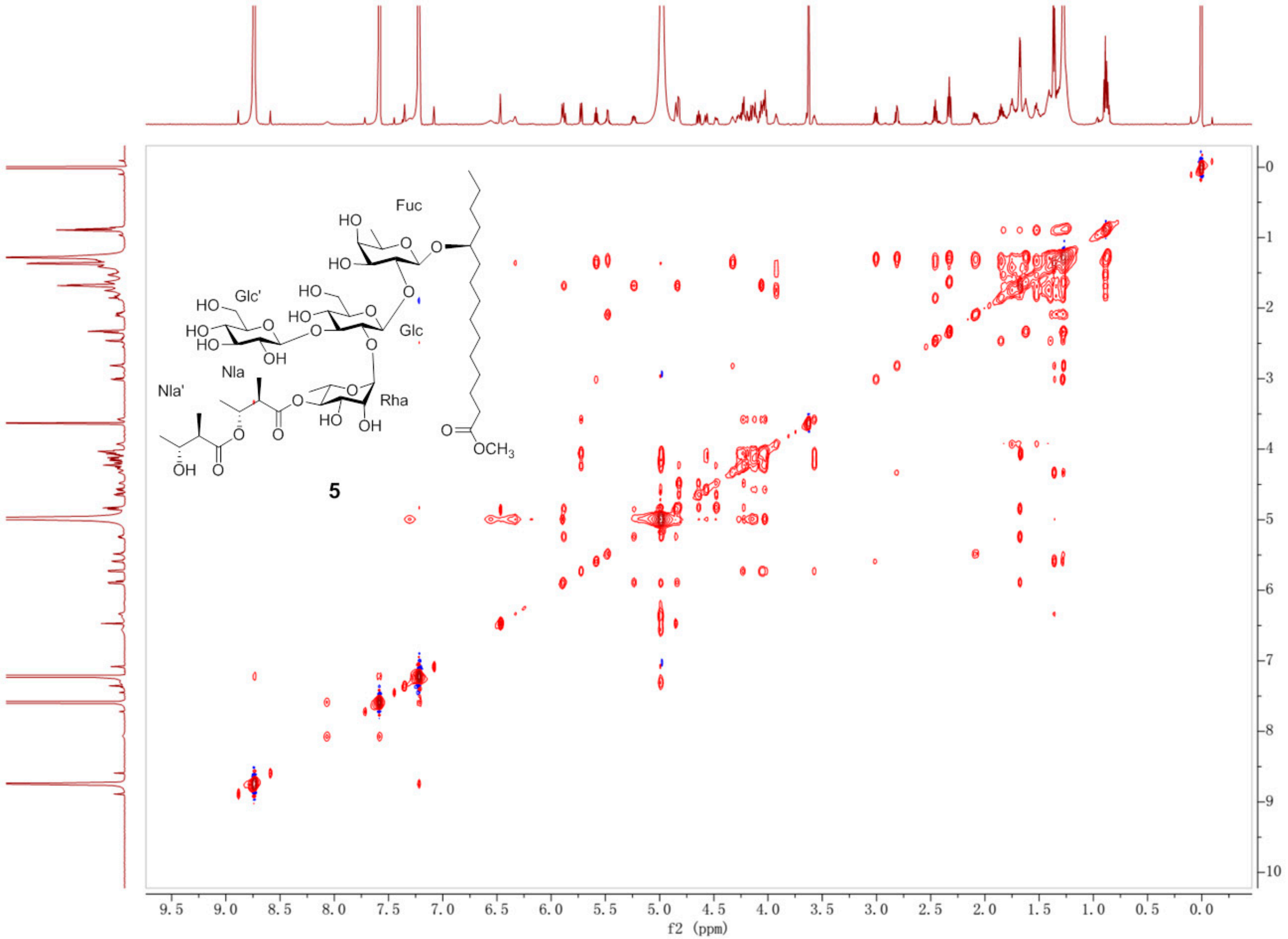

Figure S59. TOCSY spectrum of compound **5** (600 MHz, pyridine-*d*5)

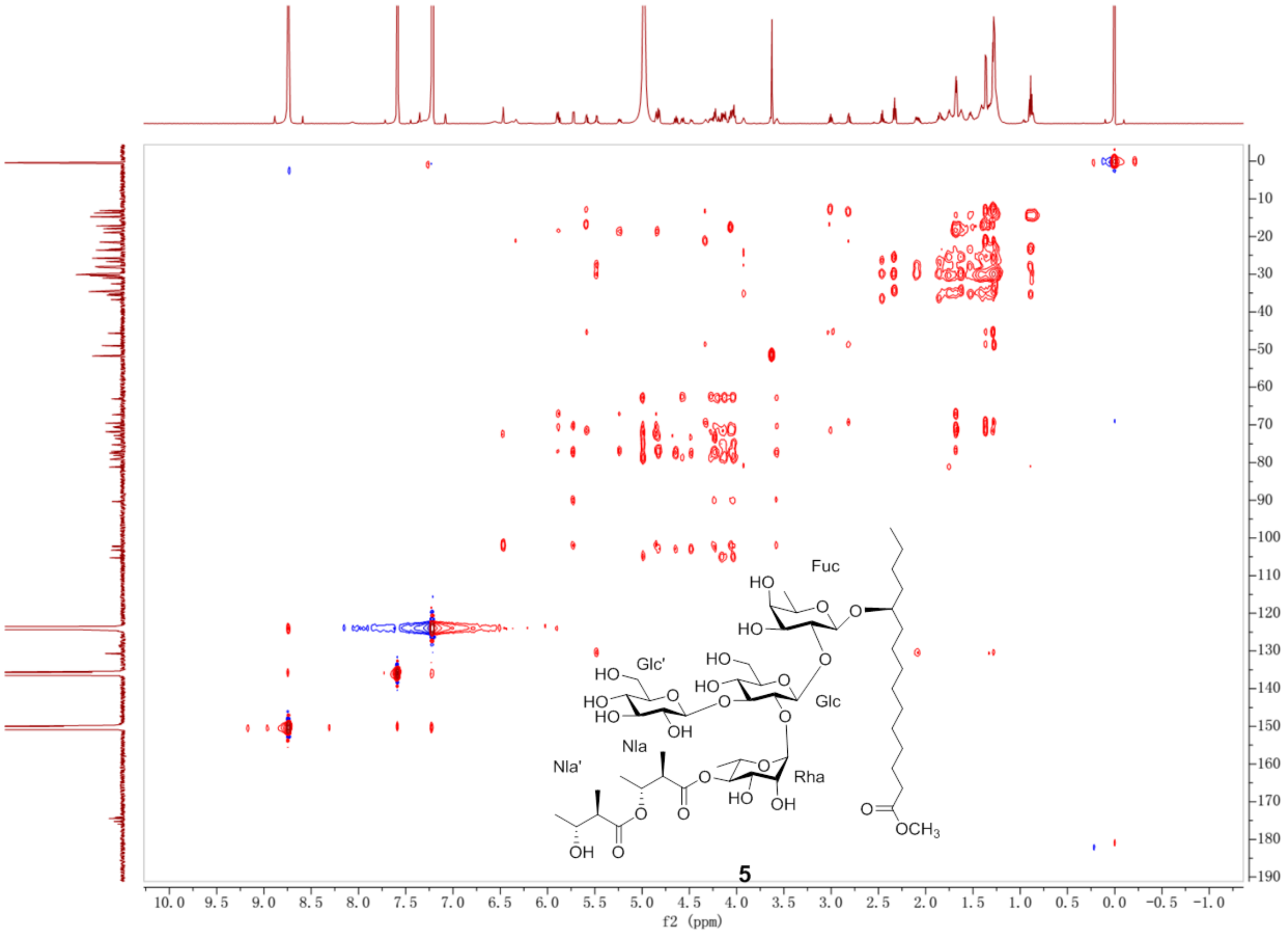

Figure S60. HSQC-TOCSY spectrum of compound **5** (600 MHz, pyridine-d_5)

Elemental Composition Report

Single Mass Analysis
Tolerance = 5.0 mDa / DBE: min = -1.5, max = 50.0
Element prediction: Off
Number of isotope peaks used for i-FIT = 3

Monoisotopic Mass, Even Electron Ions
9227 formula(e) evaluated with 1 results within limits (up to 50 best isotopic matches for each mass)
Elements Used:
C: 50-50 H: 88-88 N: 0-100 O: 0-100 Na: 0-3

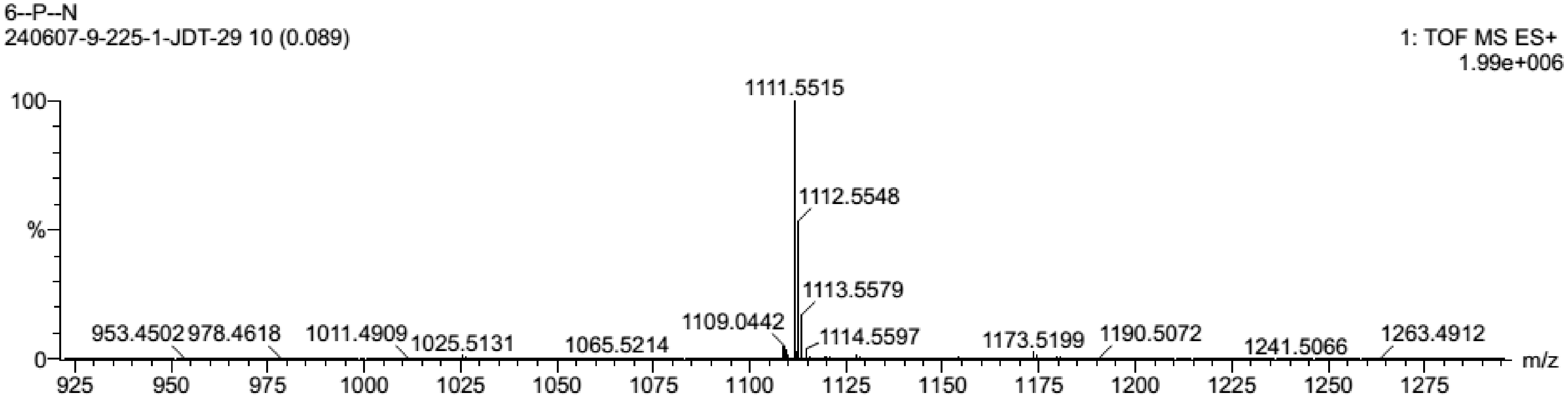

Minimum:				-1.5				
Maximum:		5.0	10.0	50.0				
Mass	Calc. Mass	mDa	PPM	DBE	i-FIT	Norm	Conf(%)	Formula
1111.5515	1111.5512	0.3	0.3	6.5	561.1	n/a	n/a	C50 H88 O25 Na

Figure S61. HRESIMS spectrum of compound **5**

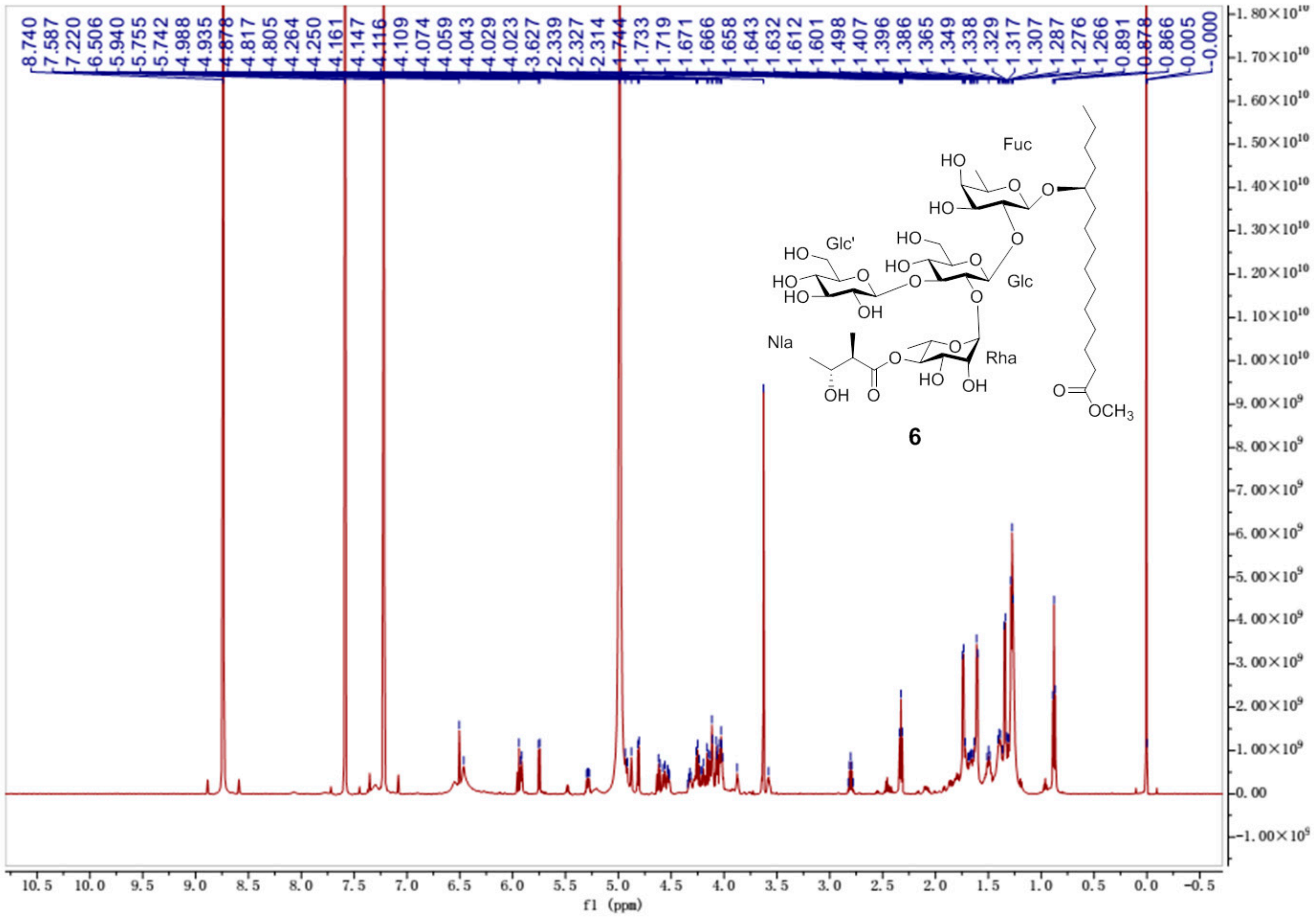

Figure S62. ^{1}H-NMR spectrum of compound **6** (600 MHz, pyridine-d_5)

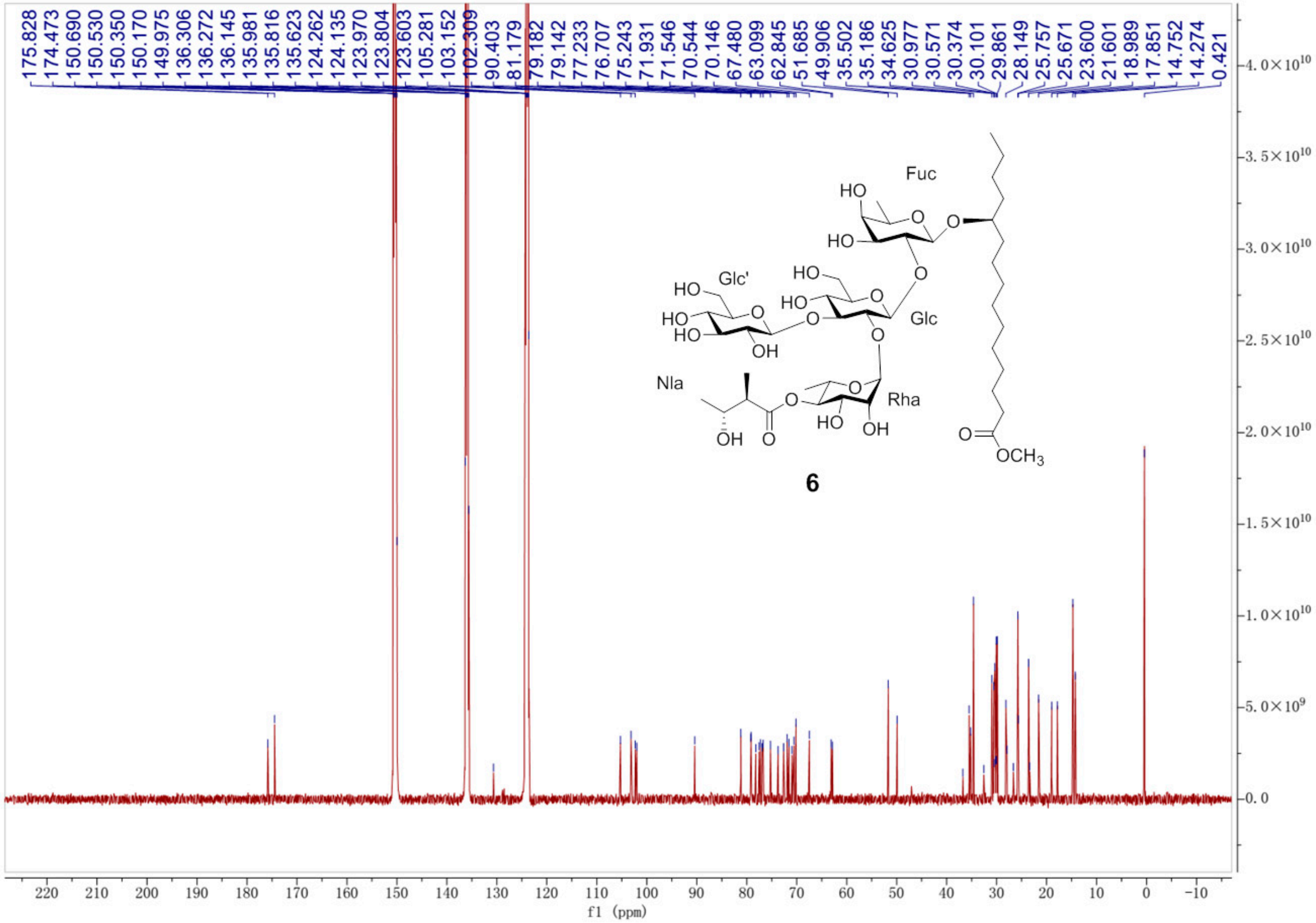

Figure S63. ^{13}C-NMR spectrum of compound **6** (151 MHz, pyridine-d_5)

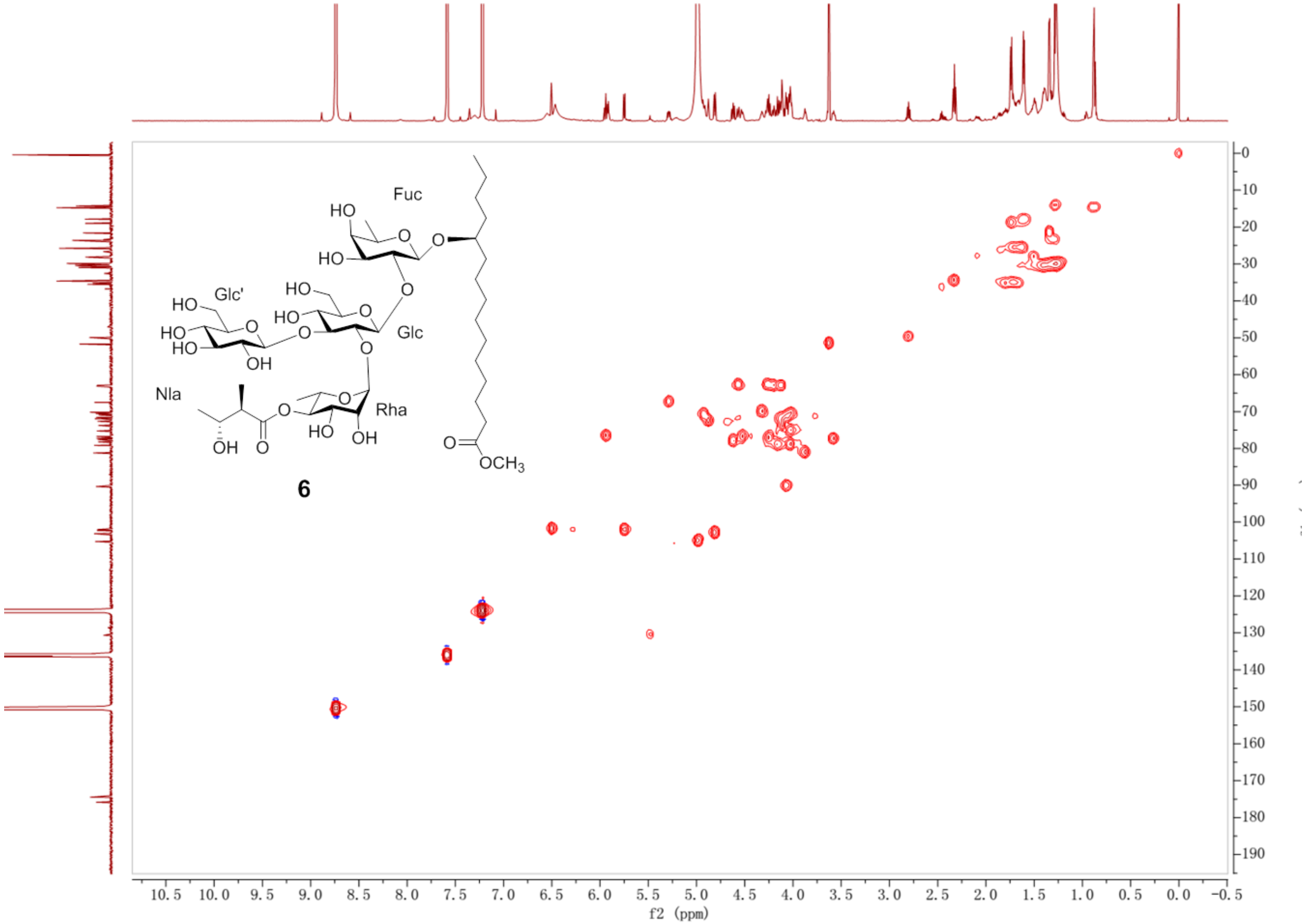

Figure S64. HSQC spectrum of compound **6** (600 MHz, pyridine-*d*5)

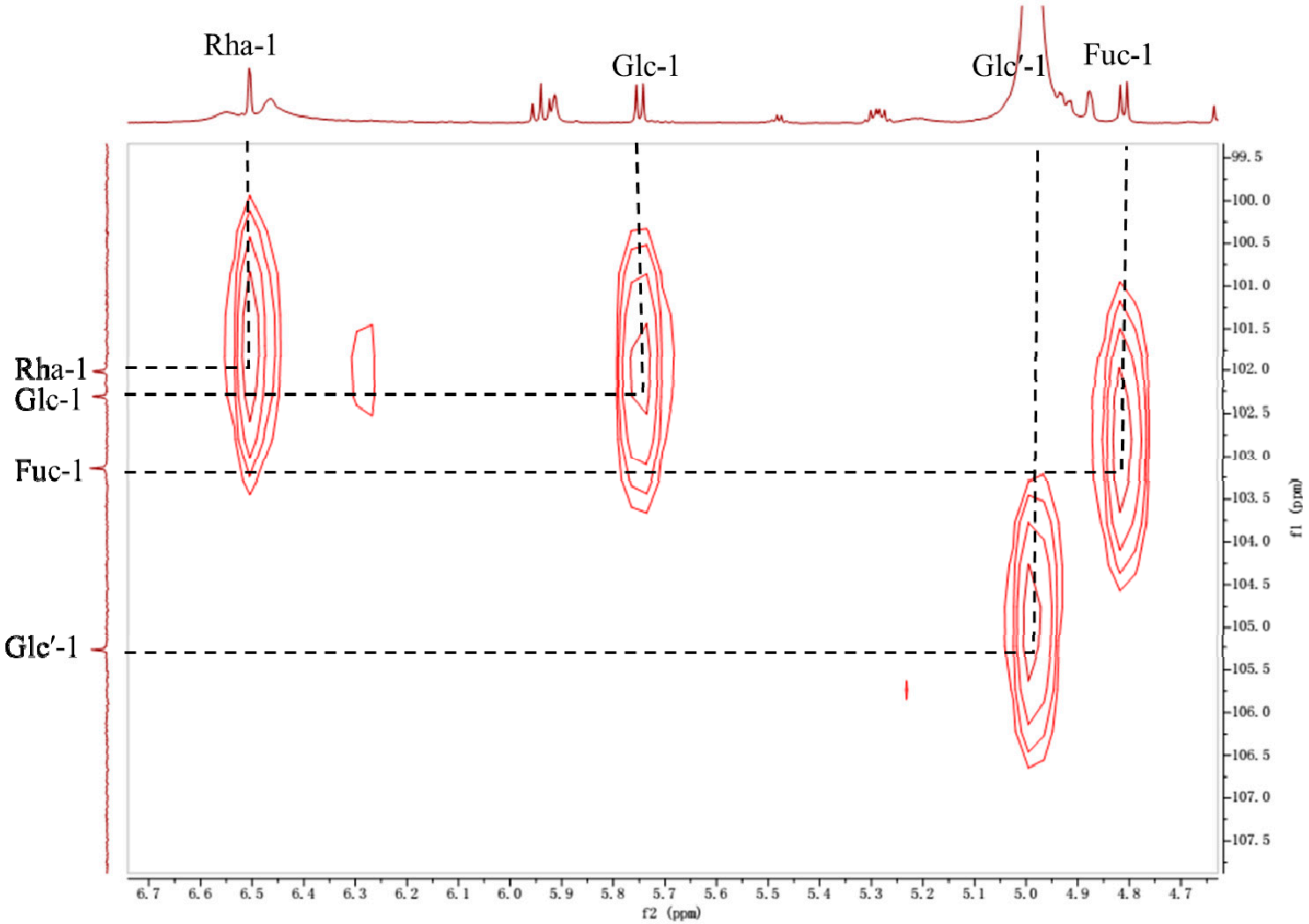

Figure S65. Expanded HSQC spectrum of compound **6** (600 MHz, pyridine-d_5)

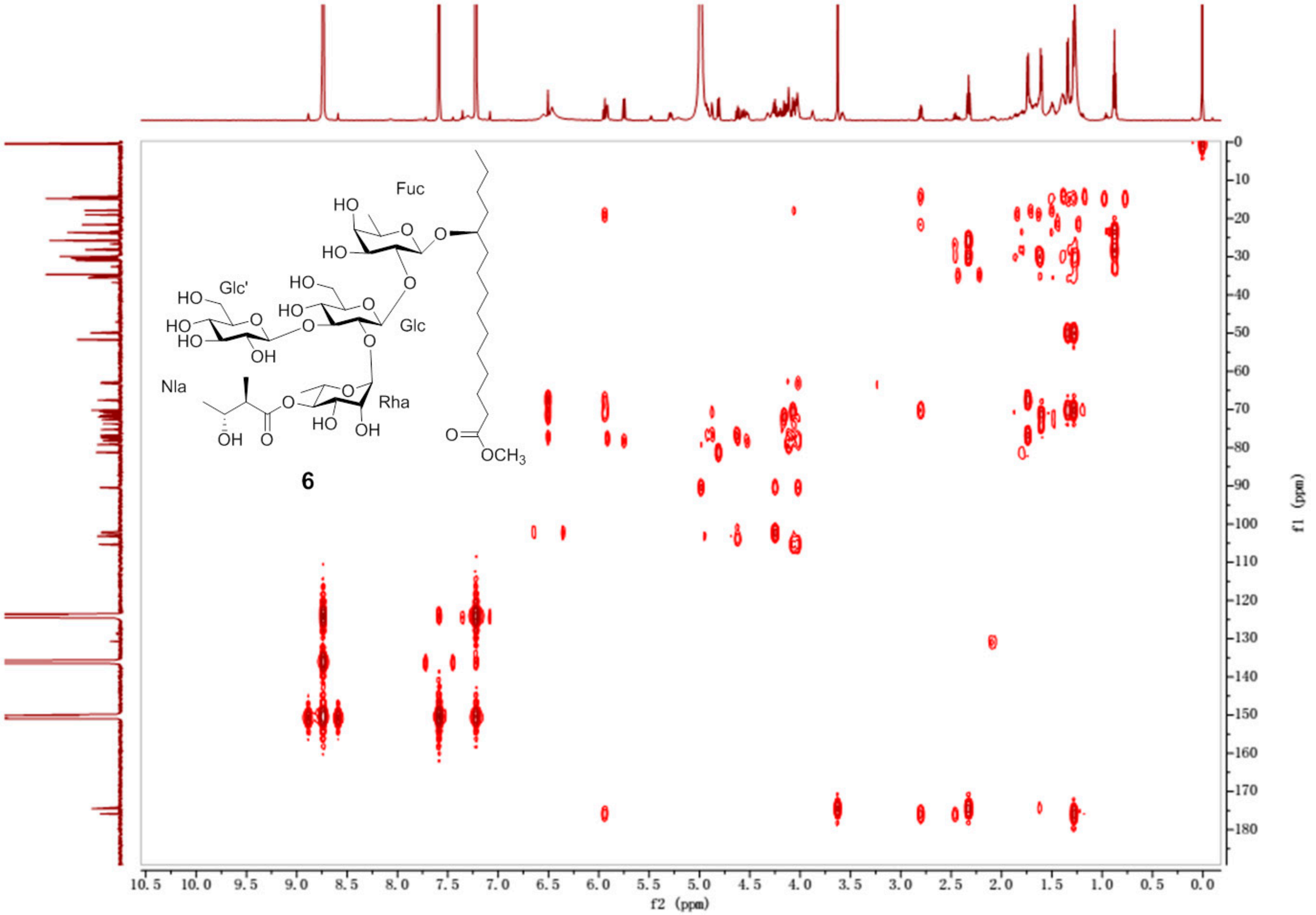

Figure S66. HMBC spectrum of compound **6** (600 MHz, pyridine-*d*5)

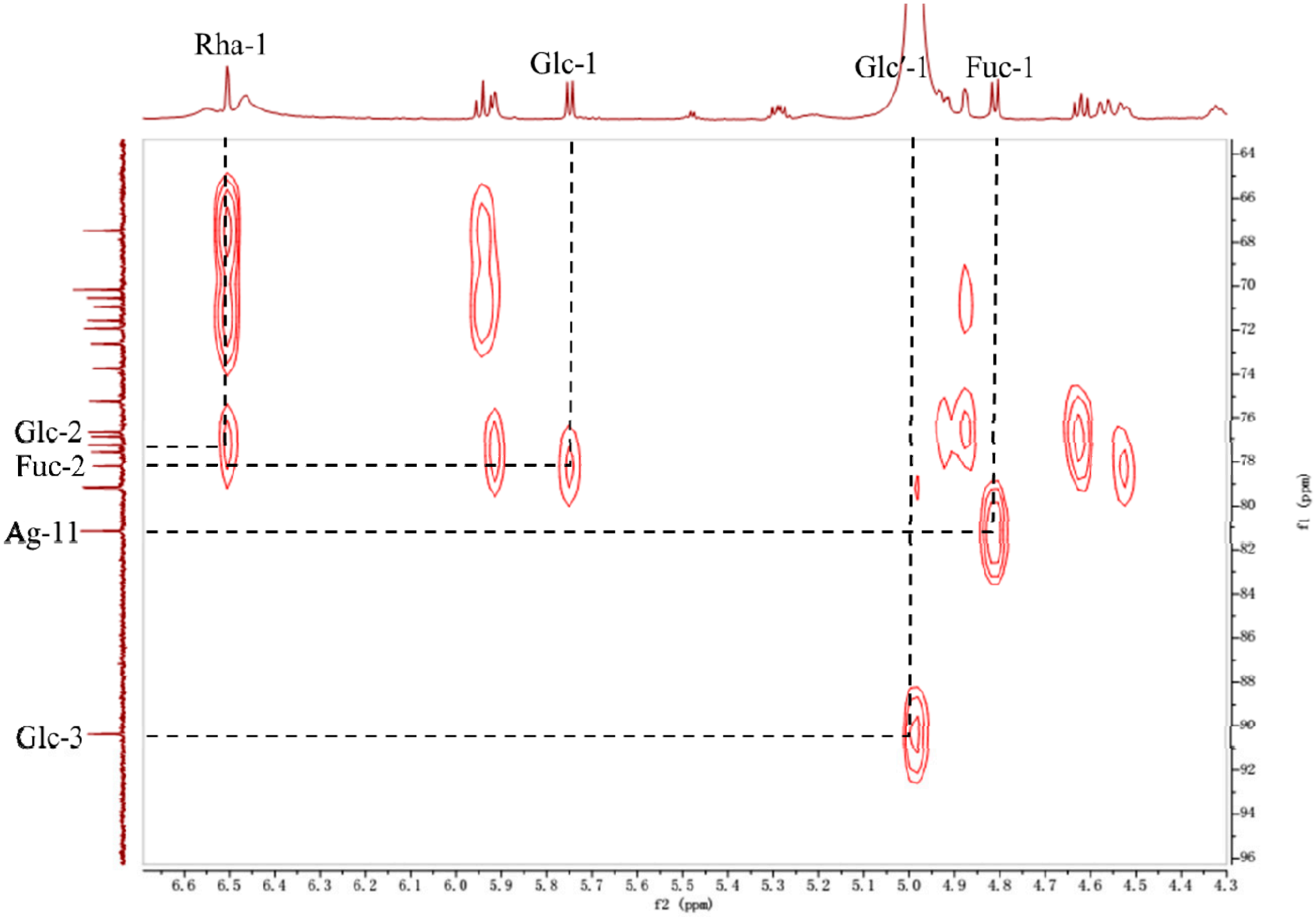

Figure S67. Expanded HMBC spectrum on the glycosidic linkages of compound **6** (600 MHz, pyridine-d_5)

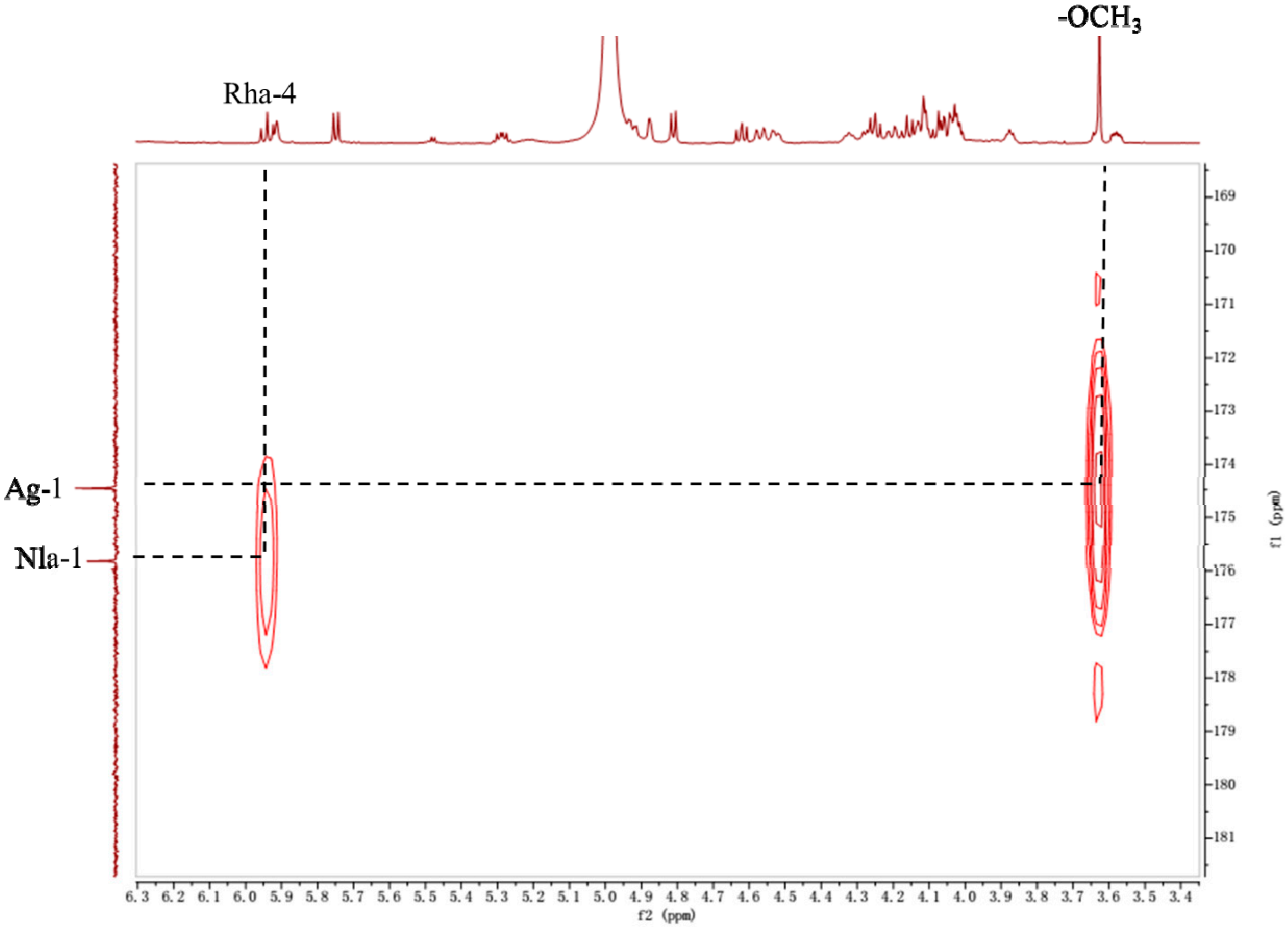

Figure S68. Expanded HMBC spectrum on the ester linkages of compound **6** (600 MHz, pyridine-d_5)

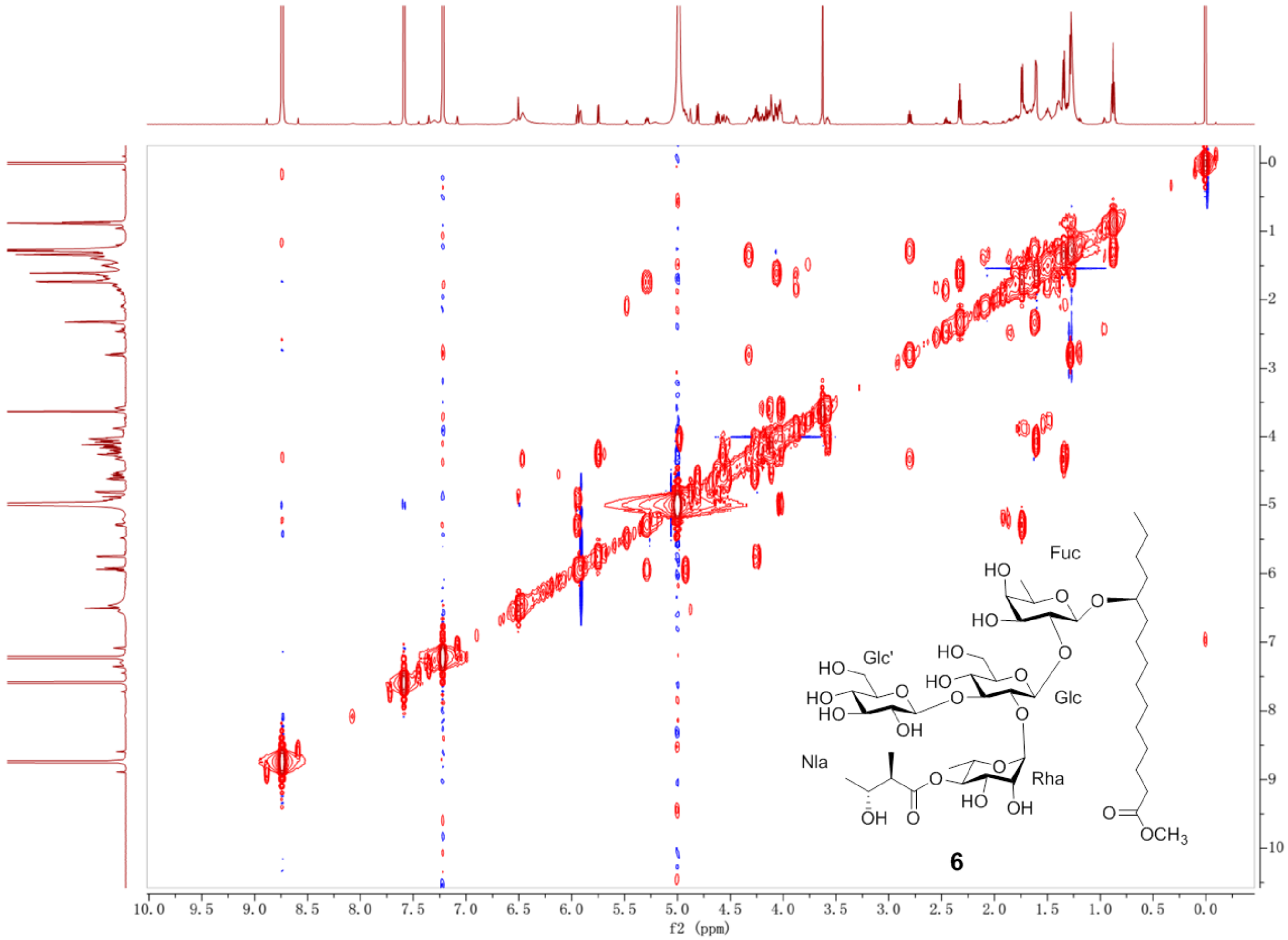

Figure S69. ^{1}H-^{1}H COSY spectrum of compound **6** (600 MHz, pyridine-*d*5)

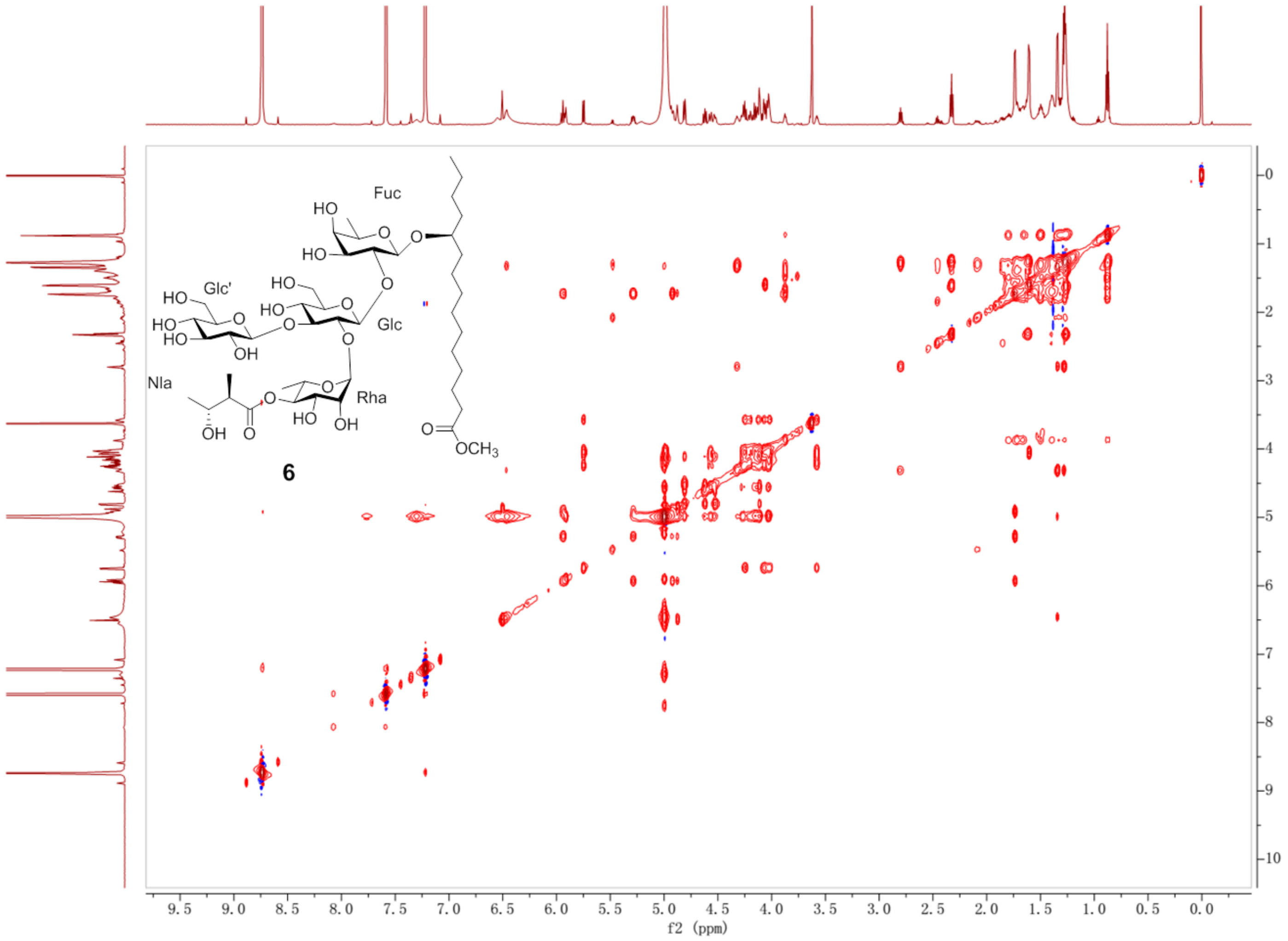

Figure S70. TOCSY spectrum of compound **6** (600 MHz, pyridine-*d*5)

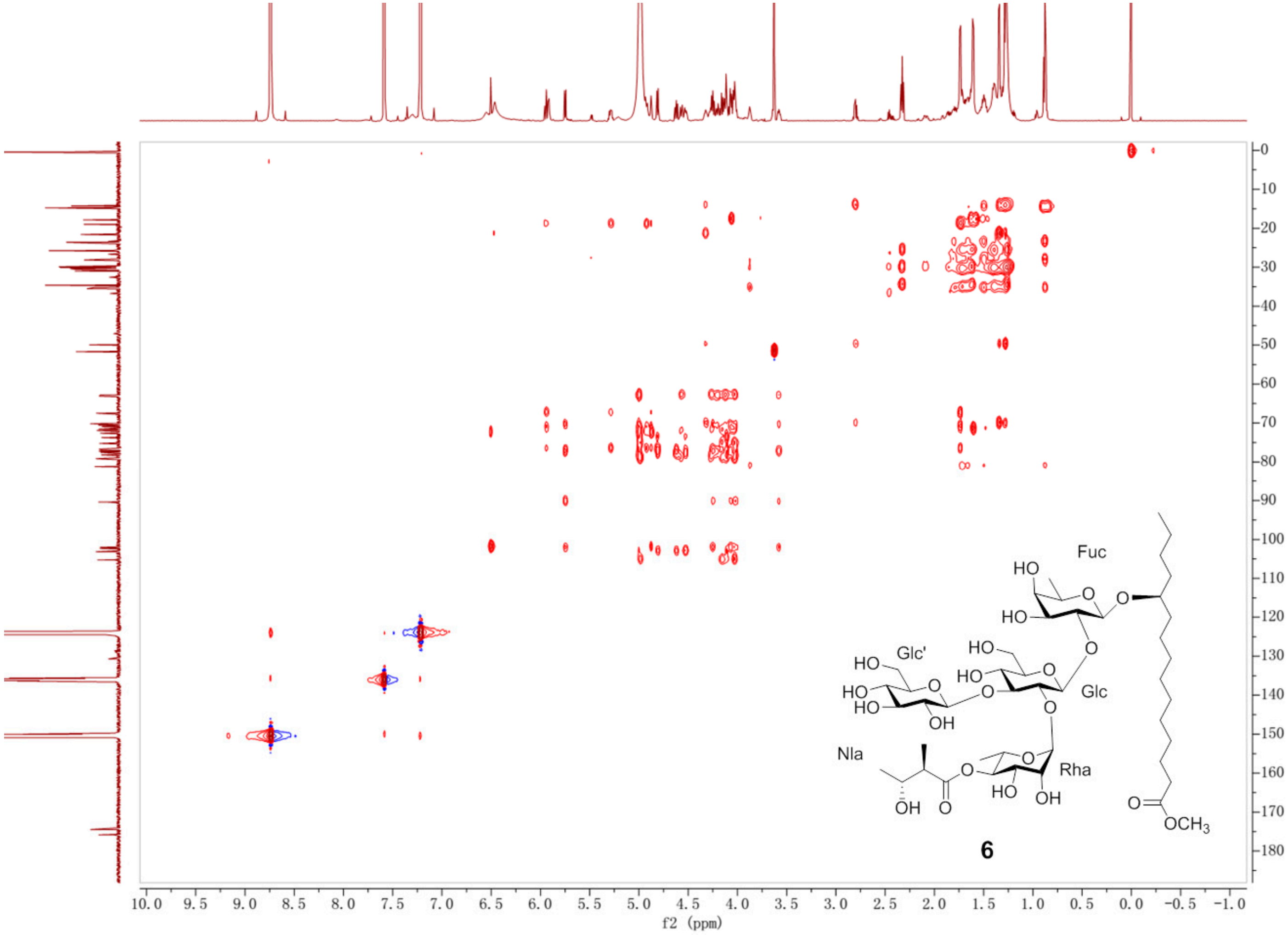

Figure S71. HSQC-TOCSY spectrum of compound **6** (600 MHz, pyridine-*d*5)

Elemental Composition Report **Page 1**

Single Mass Analysis
Tolerance = 5.0 mDa / DBE: min = -1.5, max = 50.0
Element prediction: Off
Number of isotope peaks used for i-FIT = 3

Monoisotopic Mass, Even Electron Ions
7597 formula(e) evaluated with 1 results within limits (up to 50 best isotopic matches for each mass)
Elements Used:
C: 45-45 H: 80-80 N: 0-100 O: 0-100 Na: 0-3
6--P--N
240607-9-225-1-JDT-27 31 (0.219) 1: TOF MS ES+ 6.36e+003

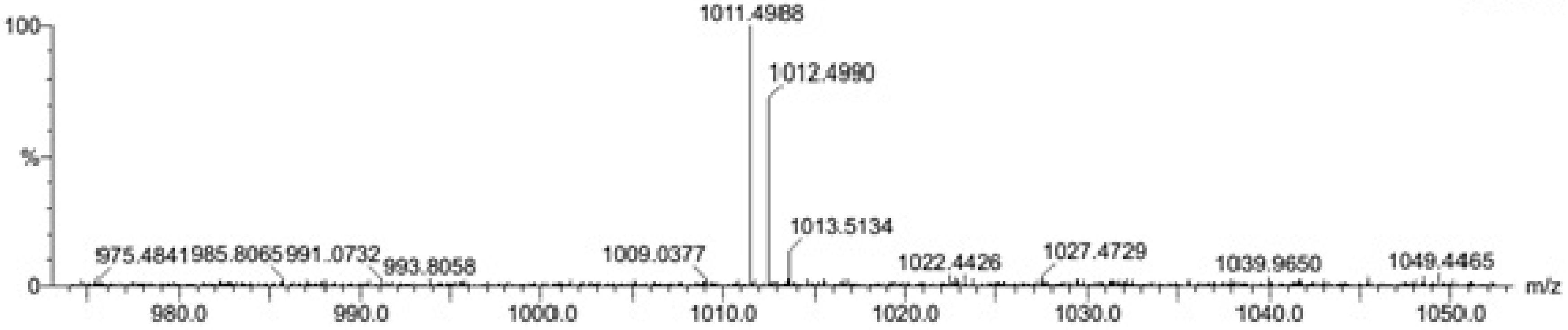

Minimum:				-1.5				
Maximum:		5.0	10.0	50.0				
Mass	Calc. Mass	mDa	PPM	DBE	i-FIT	Norm	Conf (%)	Formula
1011.4988	1011.4988	0.0	0.0	5.5	246.9	n/a	n/a	C45 H80 O23 Na

Figure S72. HRESIMS spectrum of compound **6**

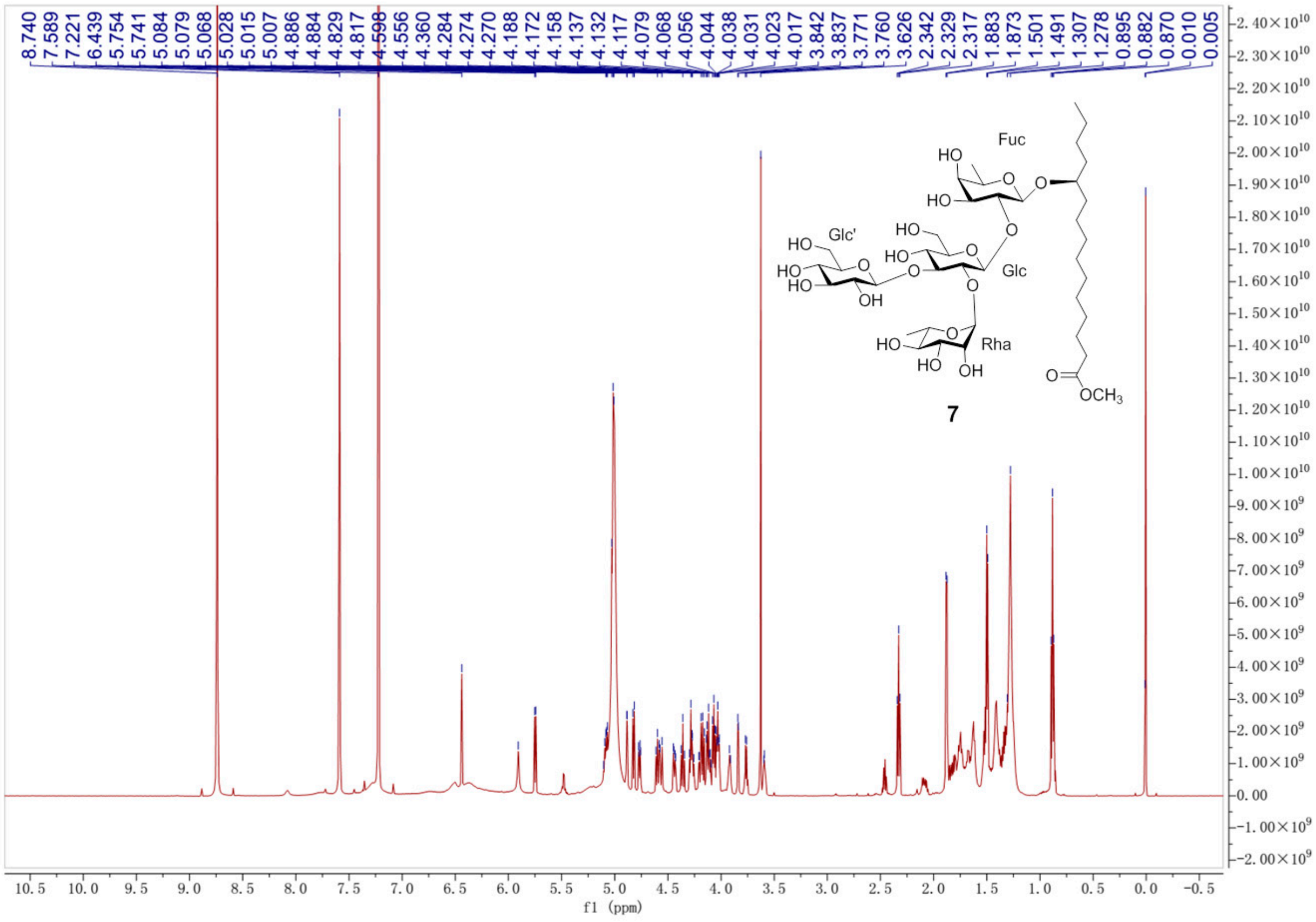

Figure S73. ^{1}H-NMR spectrum of compound **7** (600 MHz, pyridine-d_5)

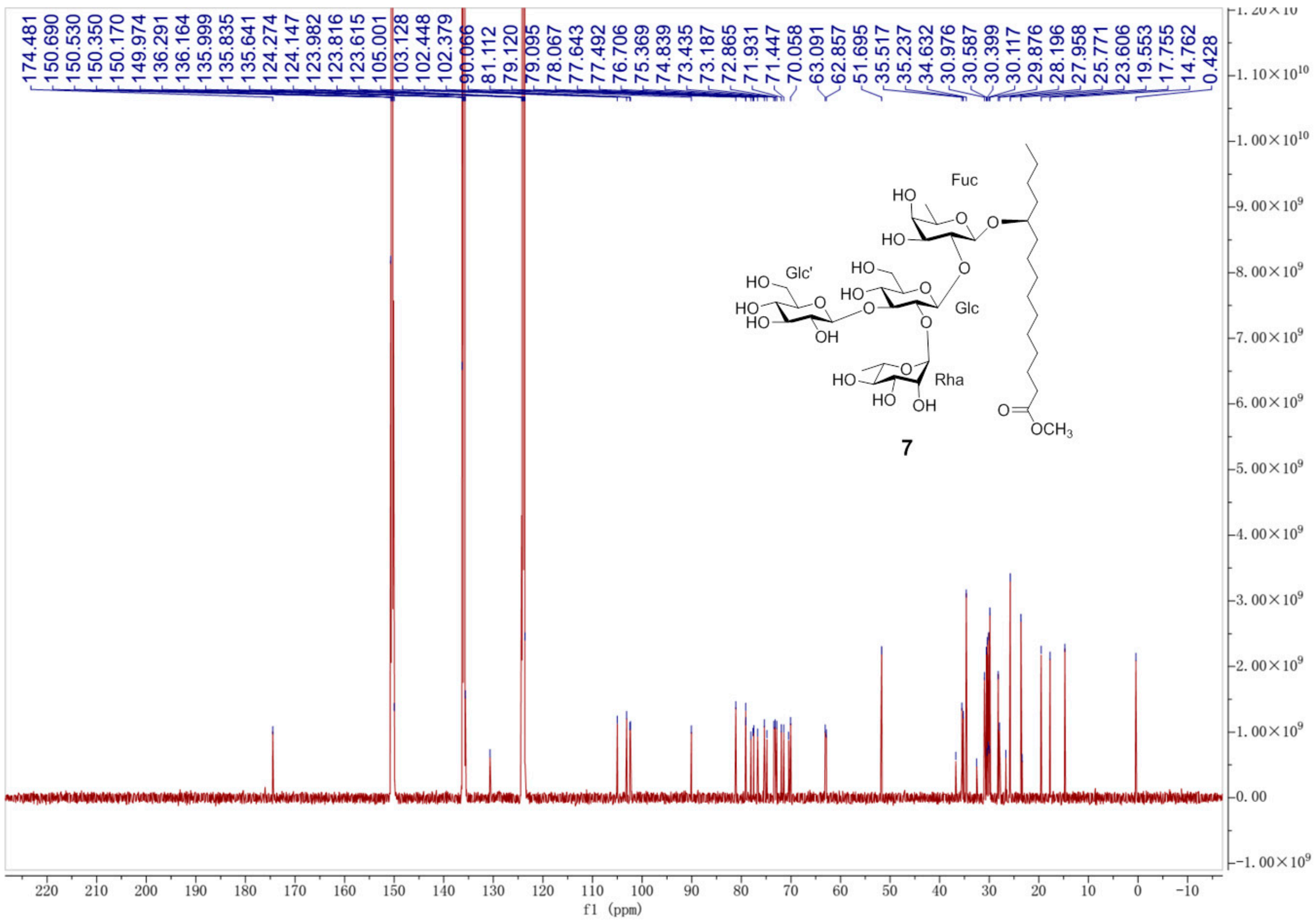

Figure S74. ^{13}C-NMR spectrum of compound **7** (151 MHz, pyridine-d_5)

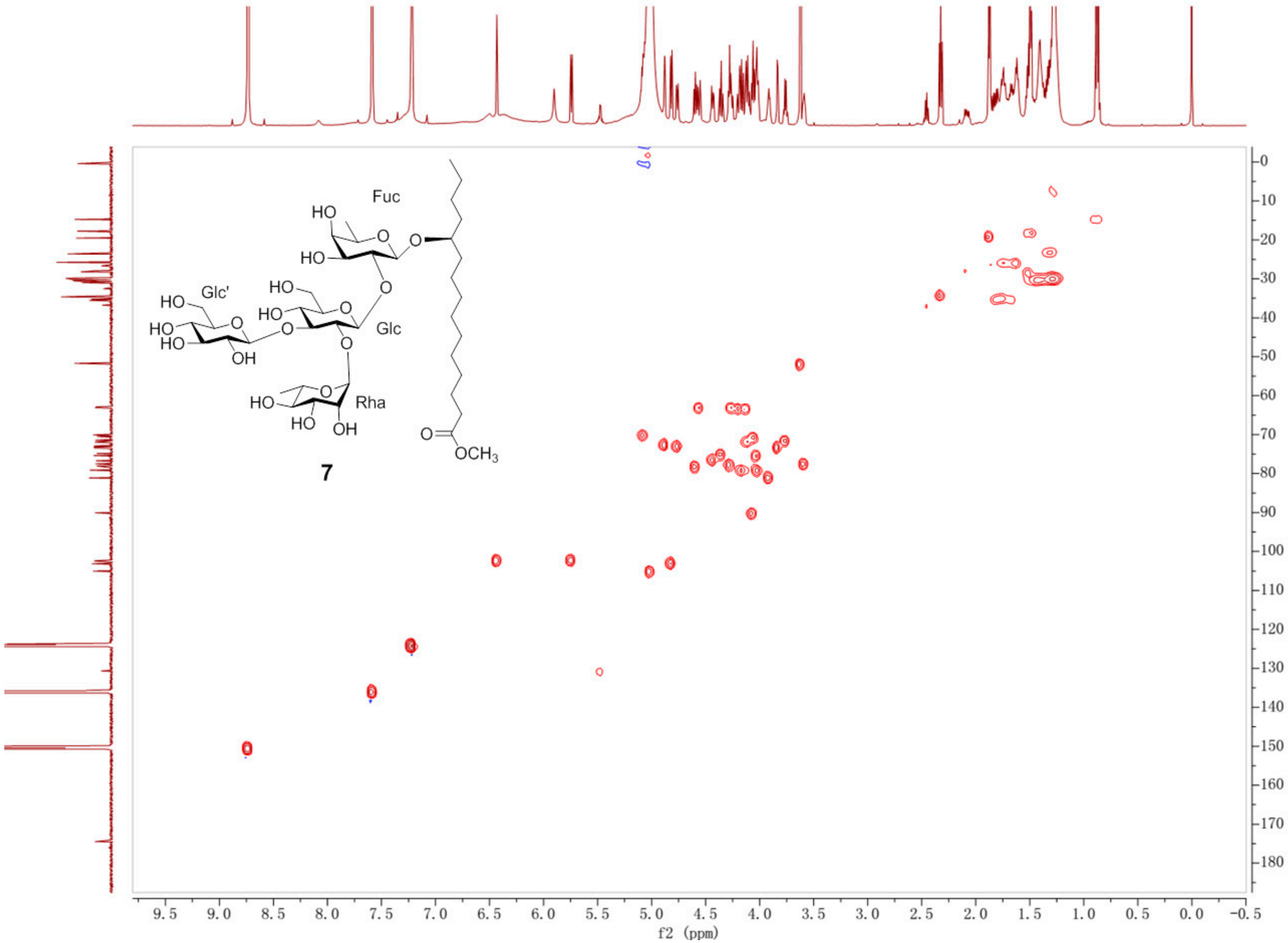

Figure S75. HSQC spectrum of compound **7** (600 MHz, pyridine-d_5)

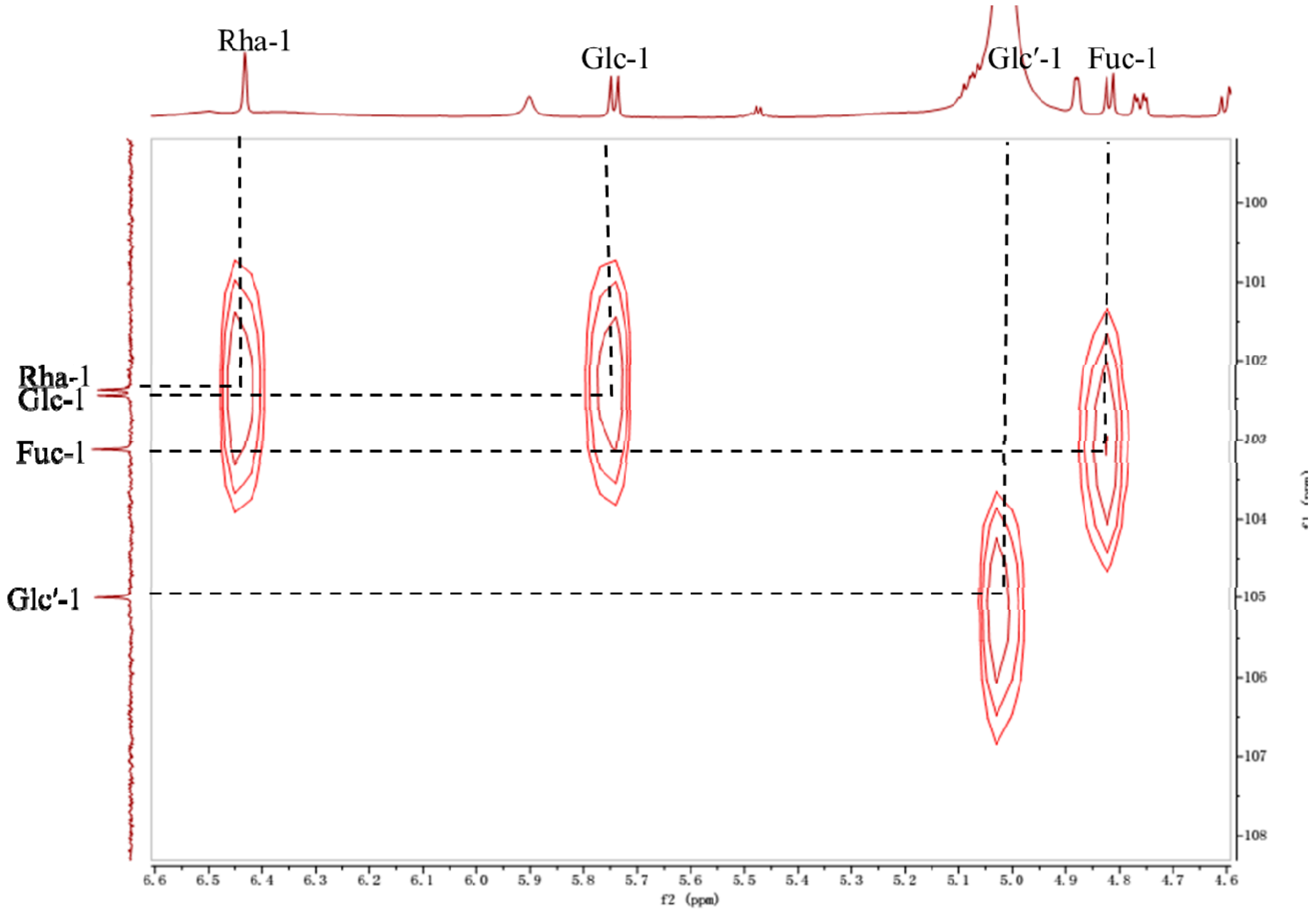

Figure S76. Expanded HSQC spectrum of compound **7** (600 MHz, pyridine-*d*5)

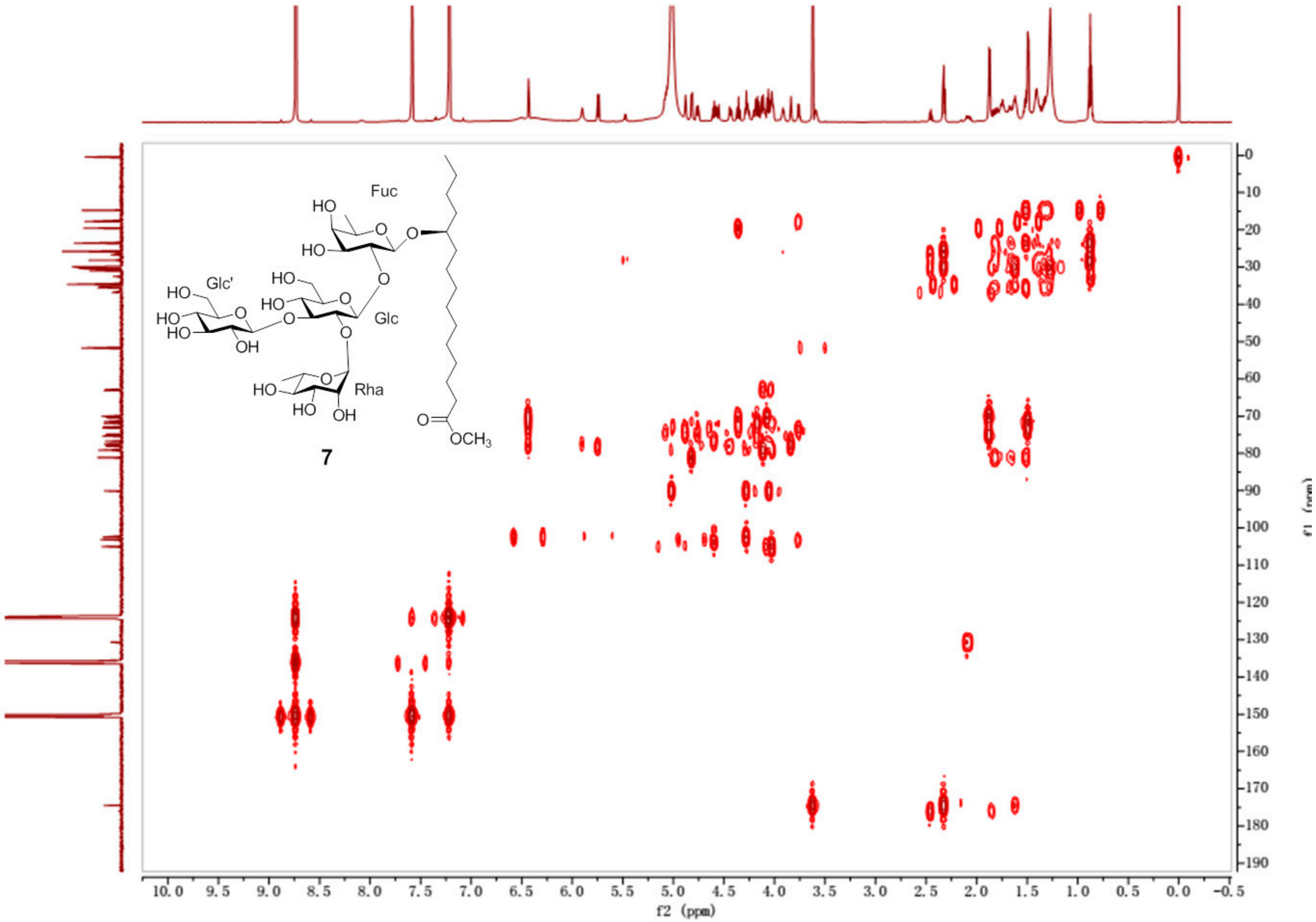

Figure S77. HMBC spectrum of compound **7** (600 MHz, pyridine-d_5)

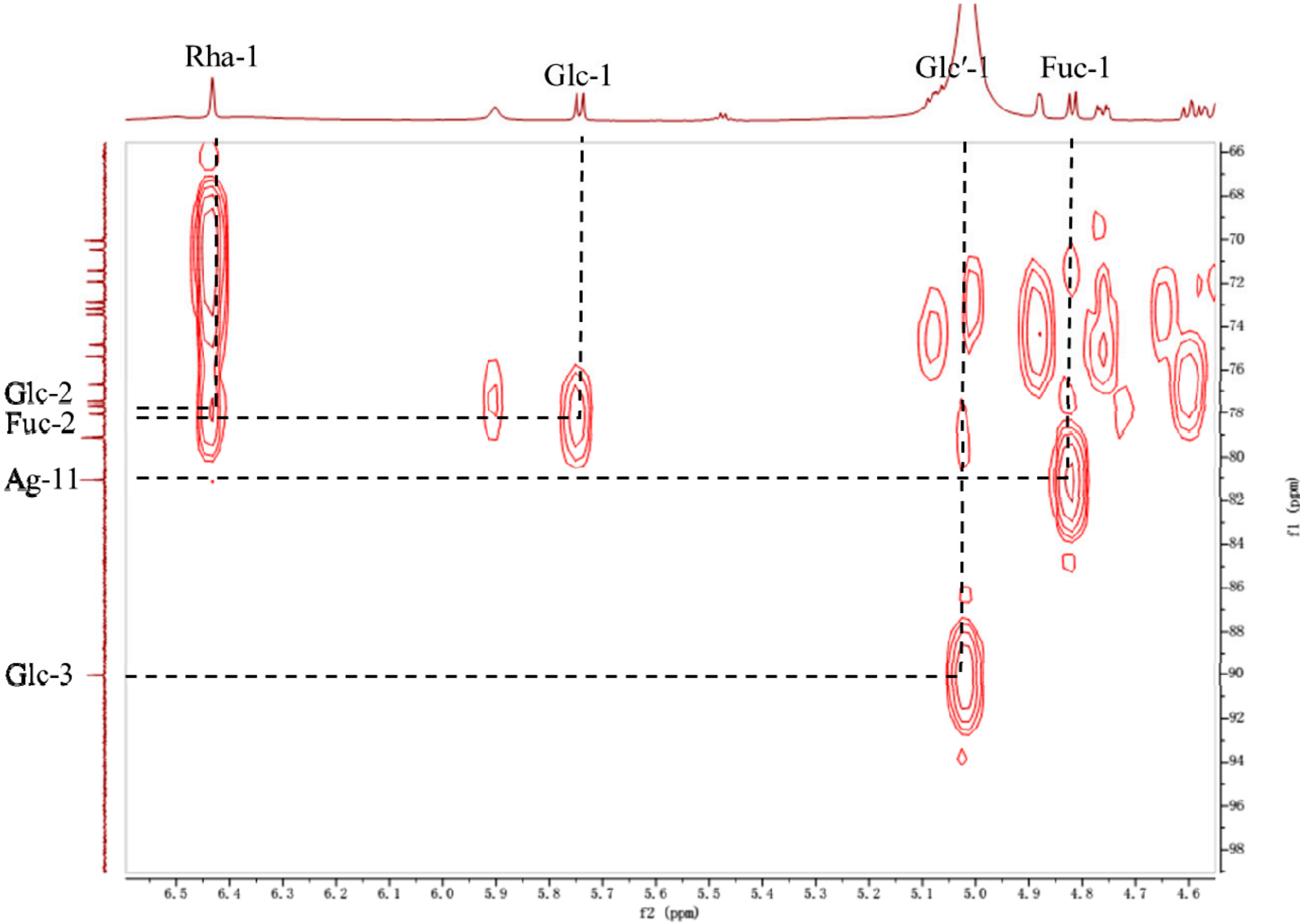

Figure S78. Expanded HMBC spectrum on the glycosidic linkages of compound **7** (600 MHz, pyridine-d_5)

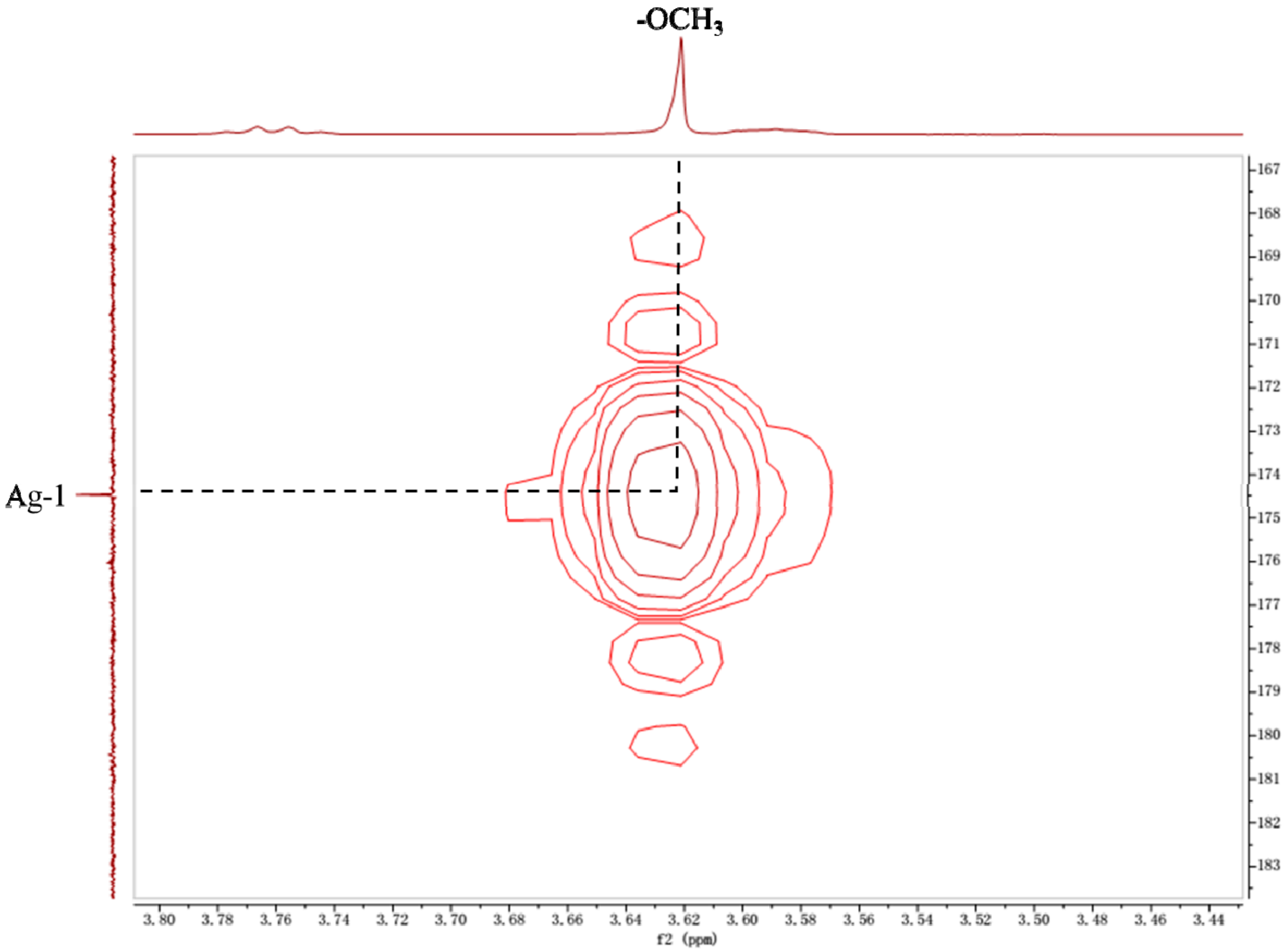

Figure S79. Expanded HMBC spectrum on the ester linkages of compound **7** (600 MHz, pyridine-d_5)

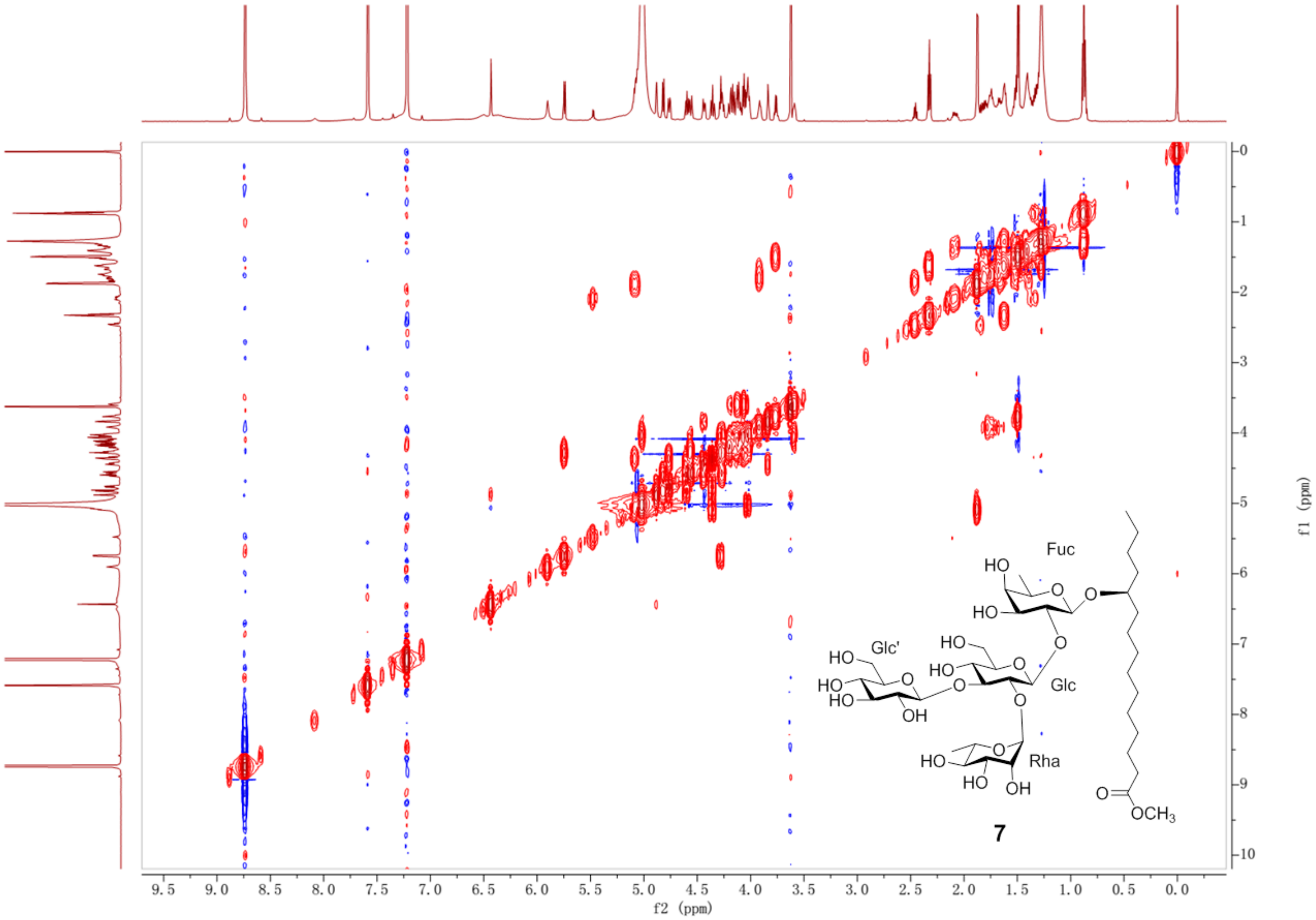

Figure S80. ^{1}H-^{1}H COSY spectrum of compound **7** (600 MHz, pyridine-d_5)

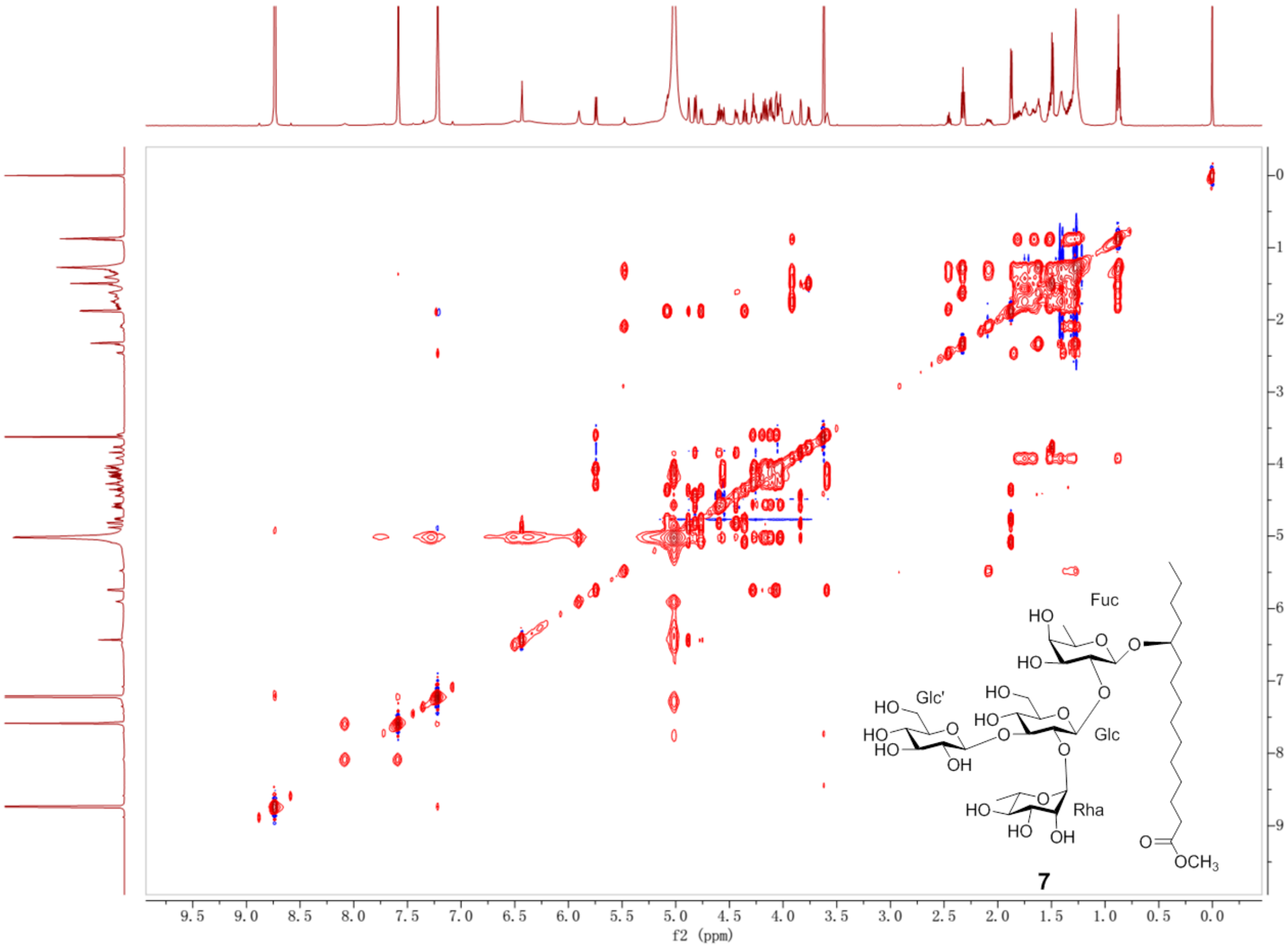

Figure S81. TOCSY spectrum of compound **7** (600 MHz, pyridine-*d*5)

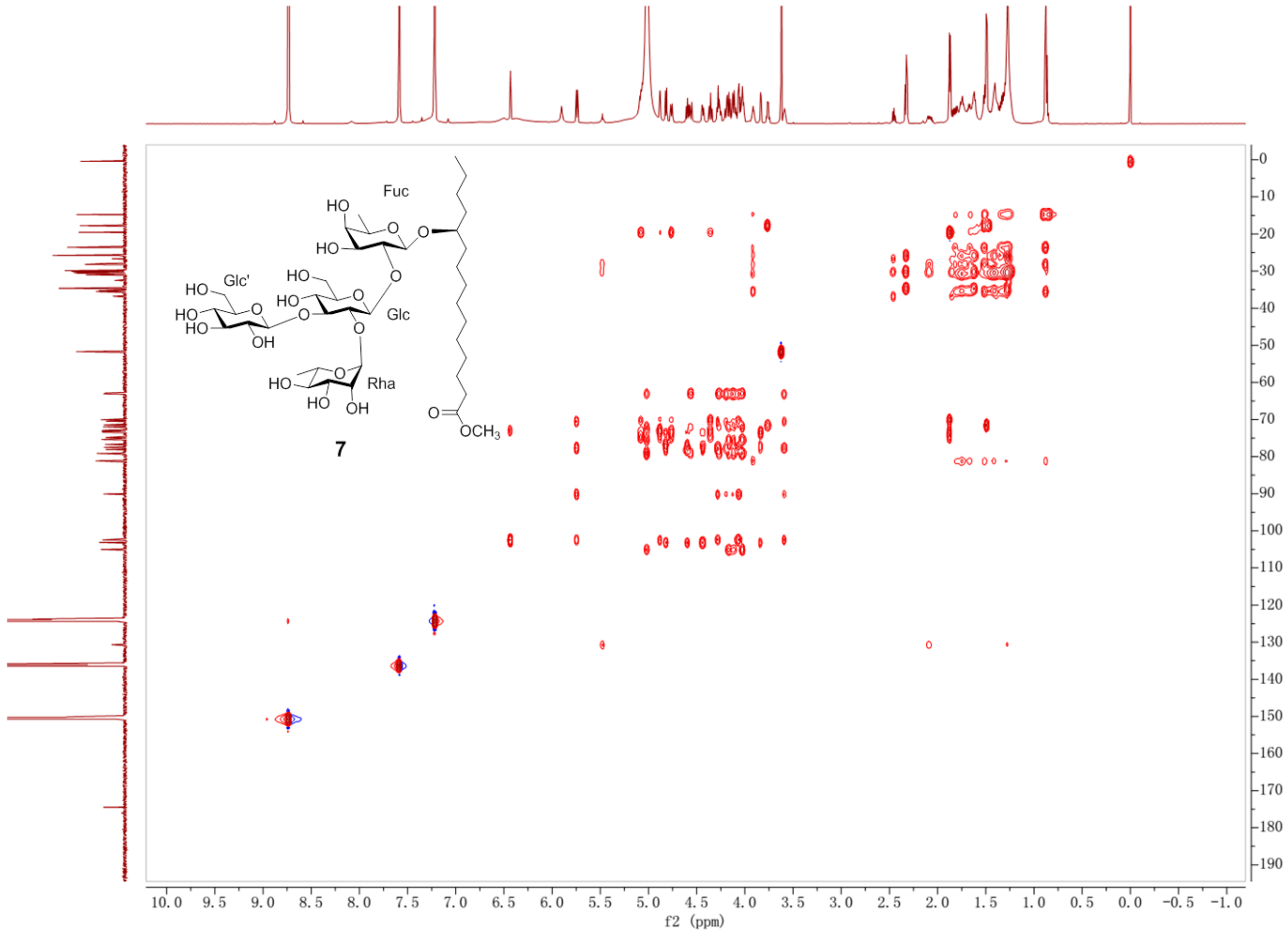

Figure S82. HSQC-TOCSY spectrum of compound **7** (600 MHz, pyridine-*d*5)

Elemental Composition Report

Single Mass Analysis

Tolerance = 5.0 mDa / DBE: min = -1.5, max = 50.0
Element prediction: Off
Number of isotope peaks used for i-FIT = 3

Monoisotopic Mass, Even Electron Ions
6123 formula(e) evaluated with 1 results within limits (up to 50 best isotopic matches for each mass)
Elements Used:
C: 40-40 H: 72-72 N: 0-100 O: 0-100 Na: 0-3
6--P--N
240607-9-225-1-JDT-25 31 (0.219)

1: TOF MS ES+
1.90e+005

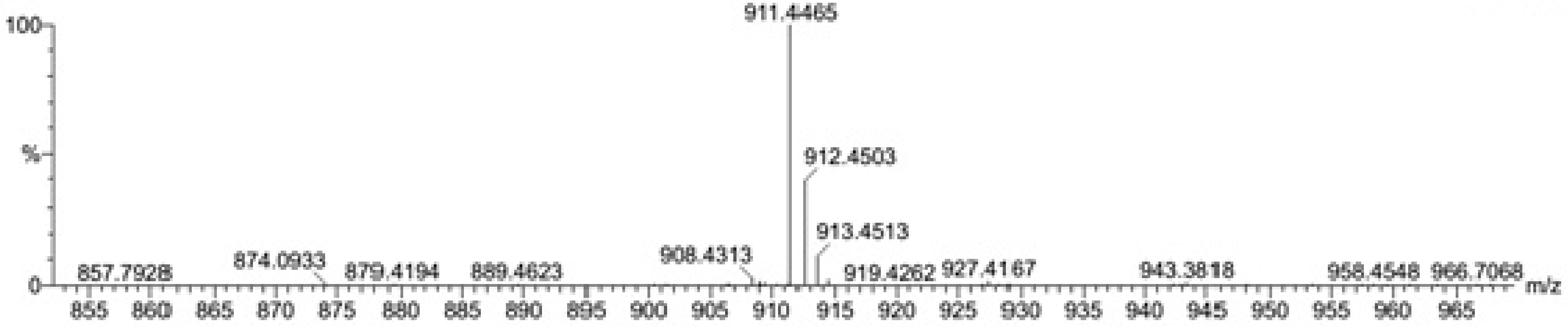

Minimum:				-1.5				
Maximum:		5.0	10.0	50.0				

Mass	Calc. Mass	mDa	PPM	DBE	i-FIT	Norm	Conf(%)	Formula
911.4465	911.4464	0.1	0.1	4.5	380.6	n/a	n/a	C40 H72 O21 Na

Figure S83. HRESIMS spectrum of compound **7**

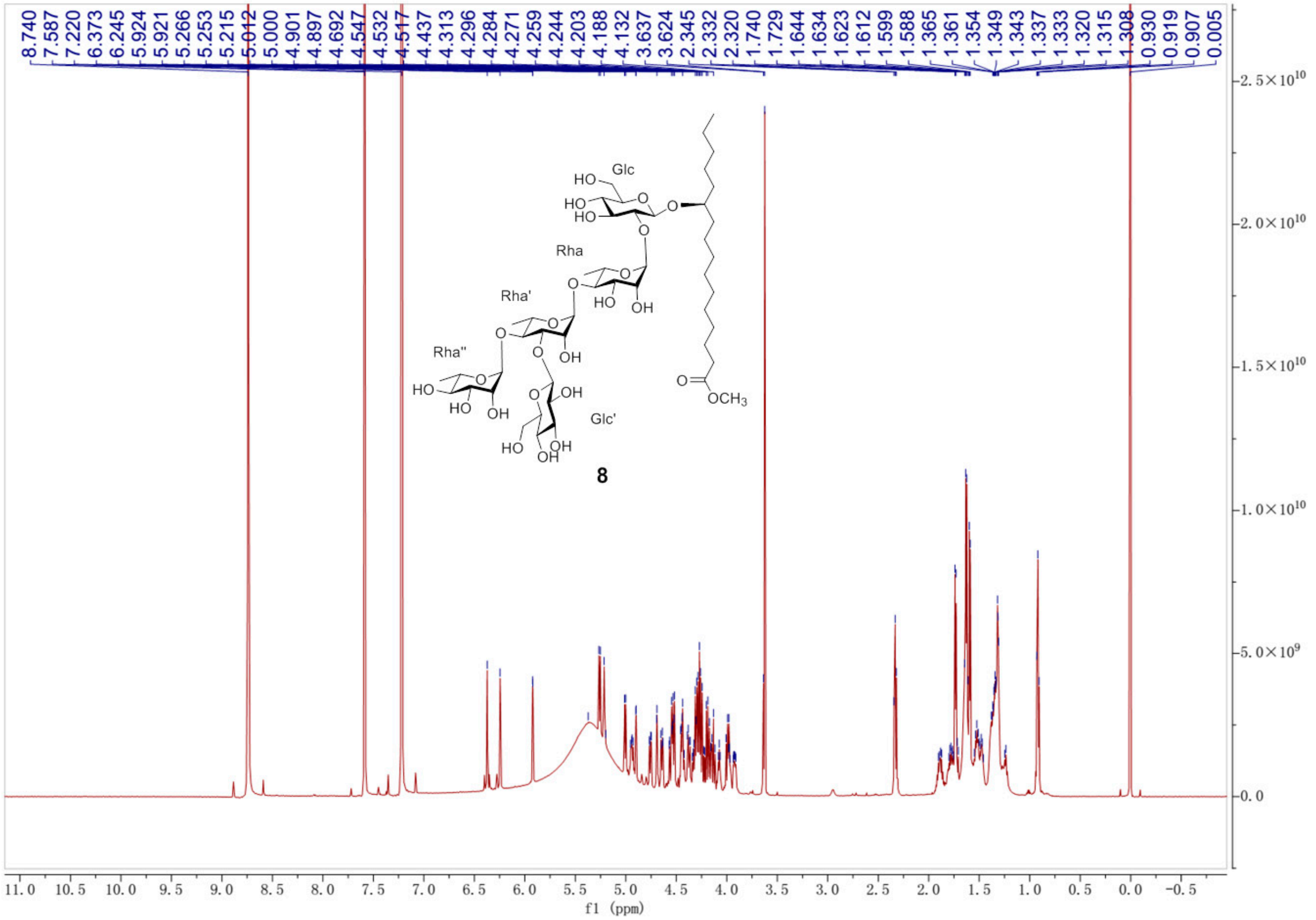

Figure S84. ^{1}H-NMR spectrum of compound **8** (600 MHz, pyridine-d_5)

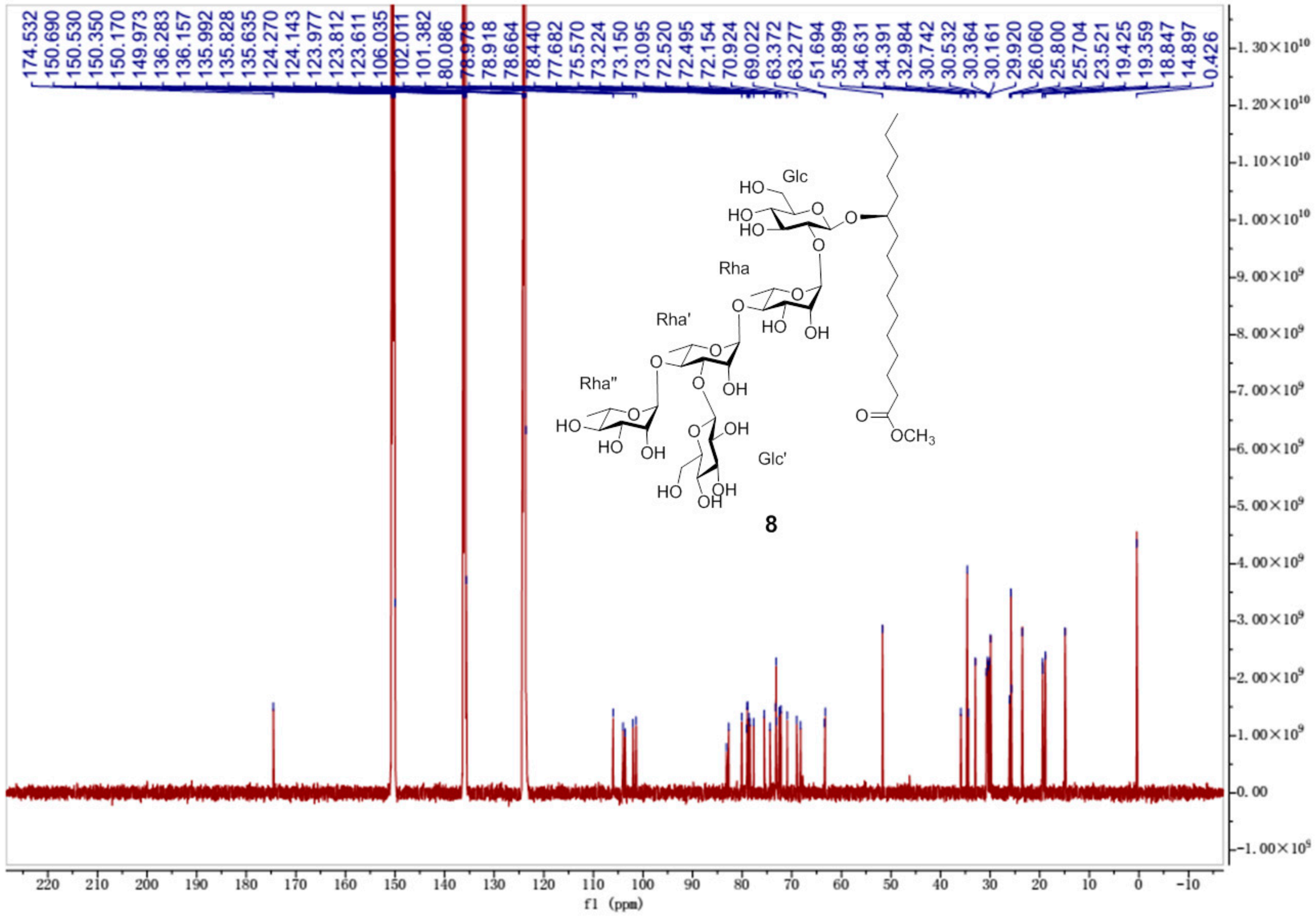

Figure S85. ^{13}C-NMR spectrum of compound **8** (151 MHz, pyridine-*d*$_5$)

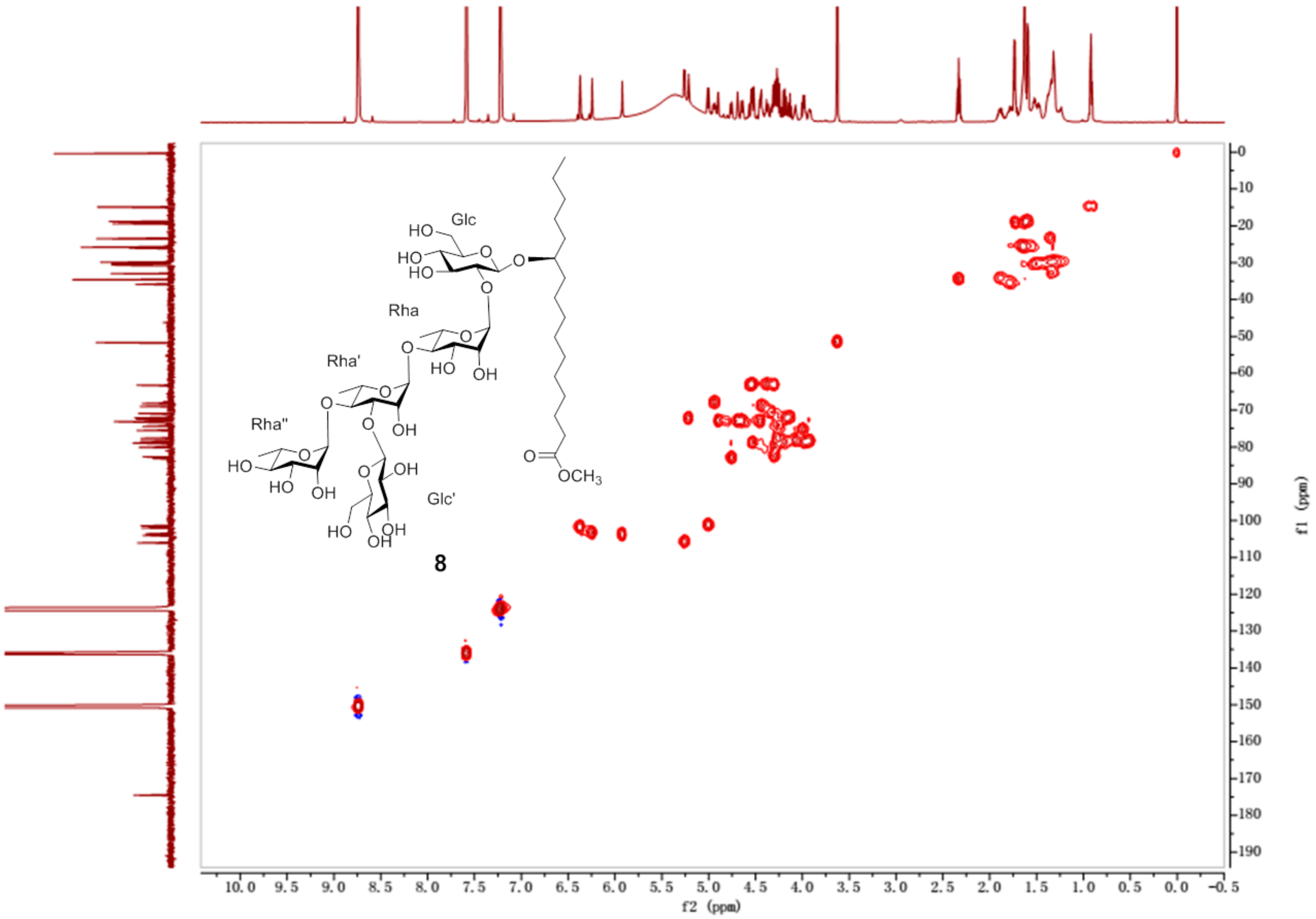

Figure S86. HSQC spectrum of compound **8** (600 MHz, pyridine-d_5)

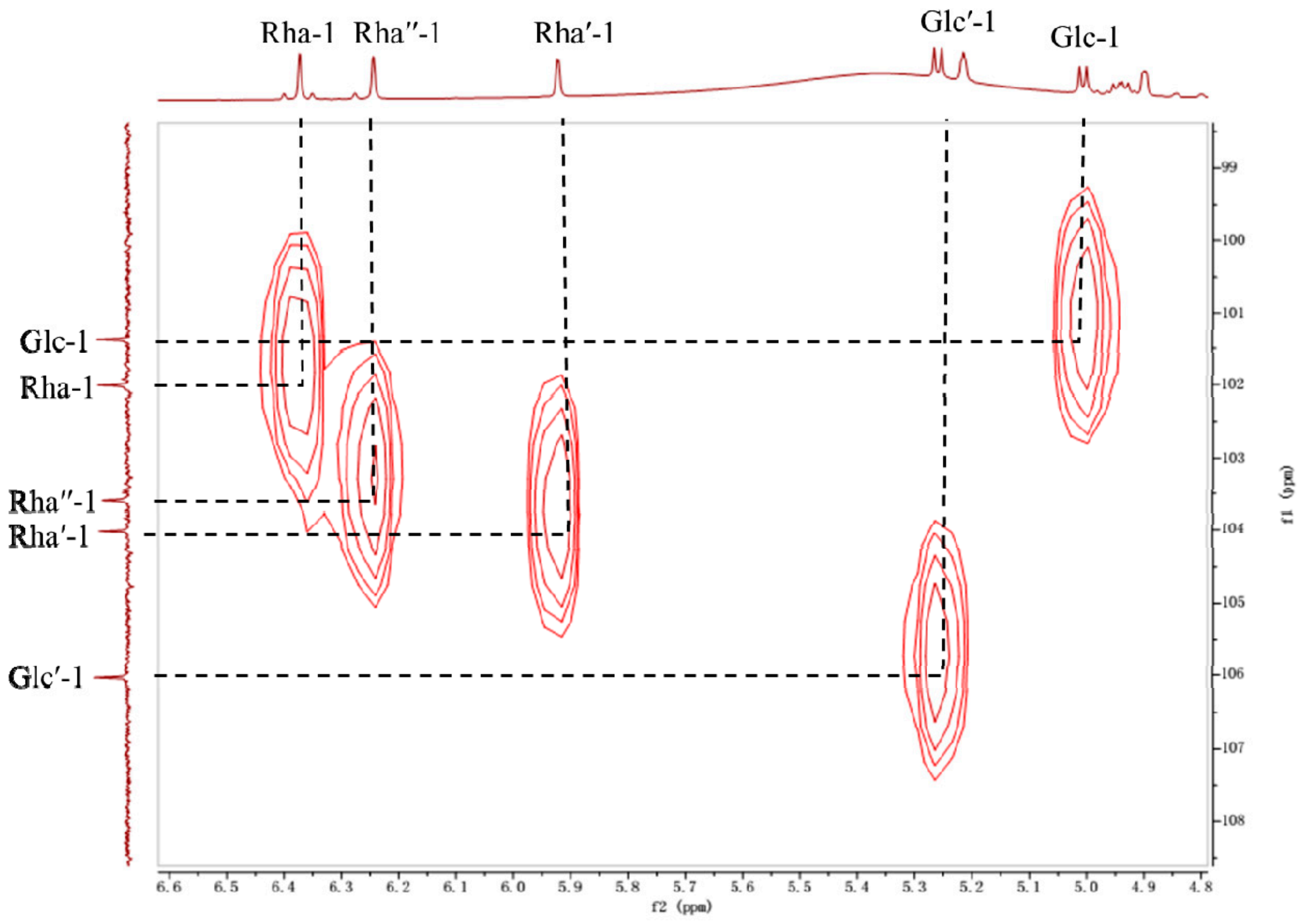

Figure S87. Expanded HSQC spectrum of compound **8** (600 MHz, pyridine-d_5)

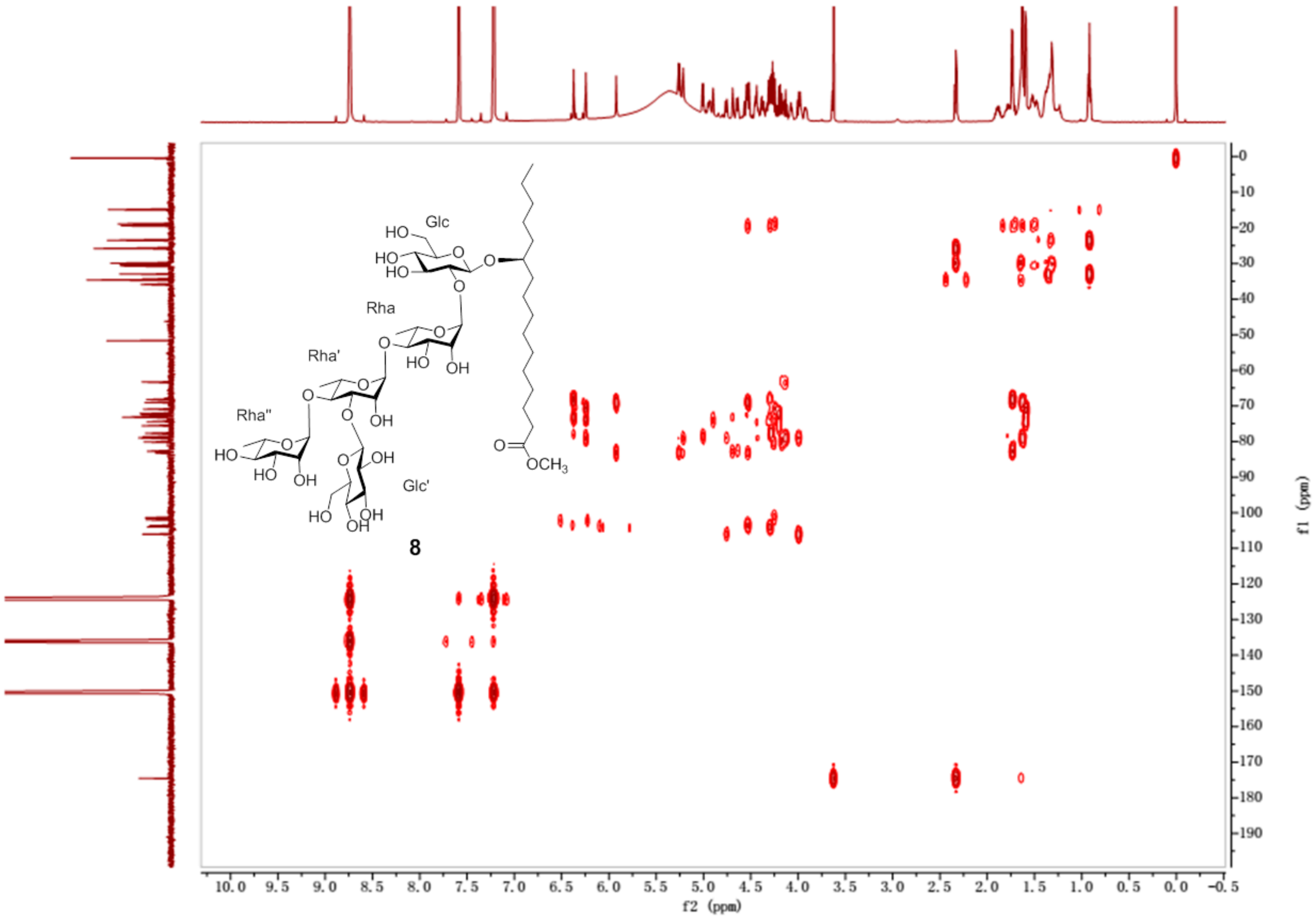

Figure S88. HMBC spectrum of compound **8** (600 MHz, pyridine-d_5)

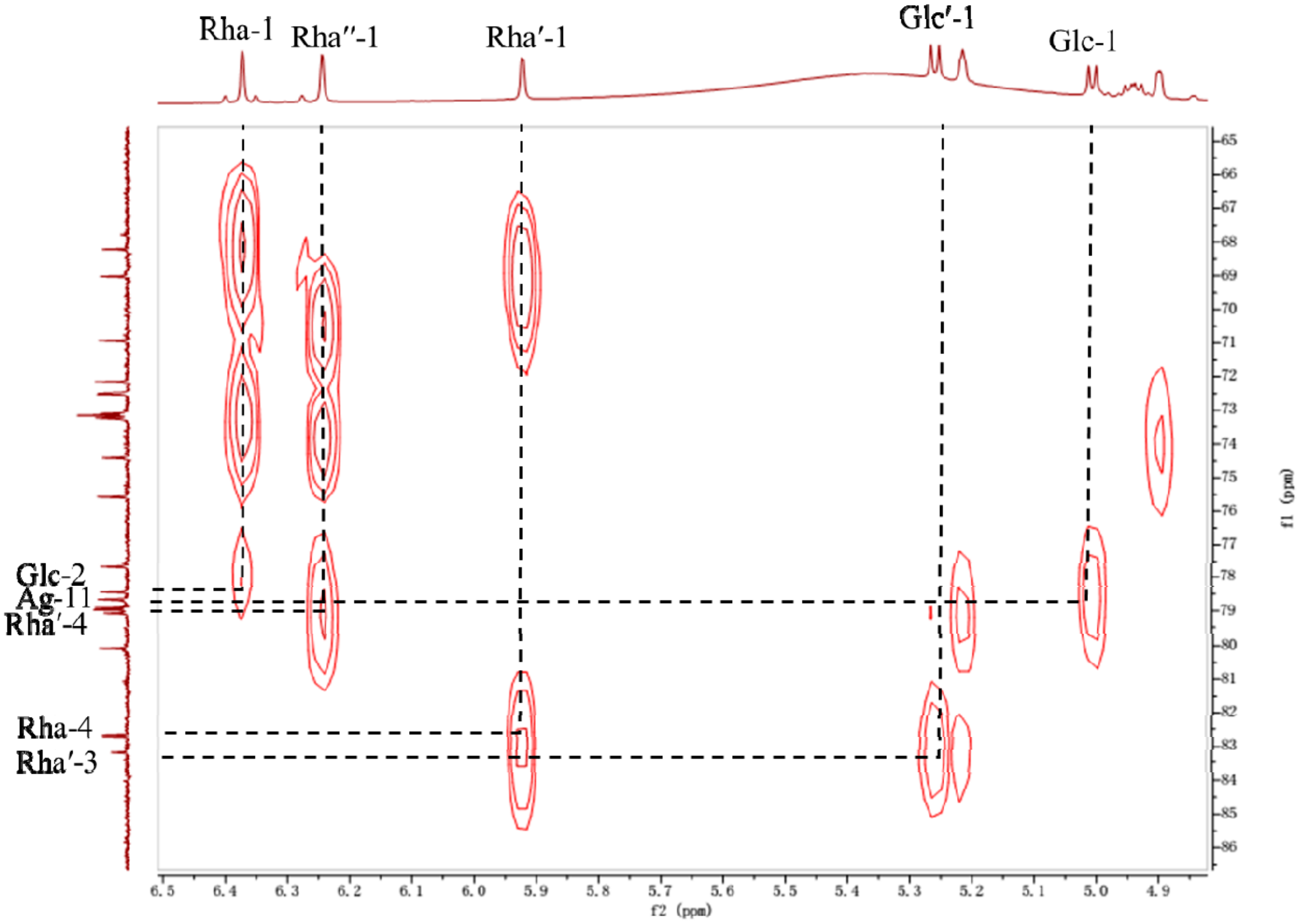

Figure S89. Expanded HMBC spectrum on the glycosidic linkages of compound **8** (600 MHz, pyridine-d_5)

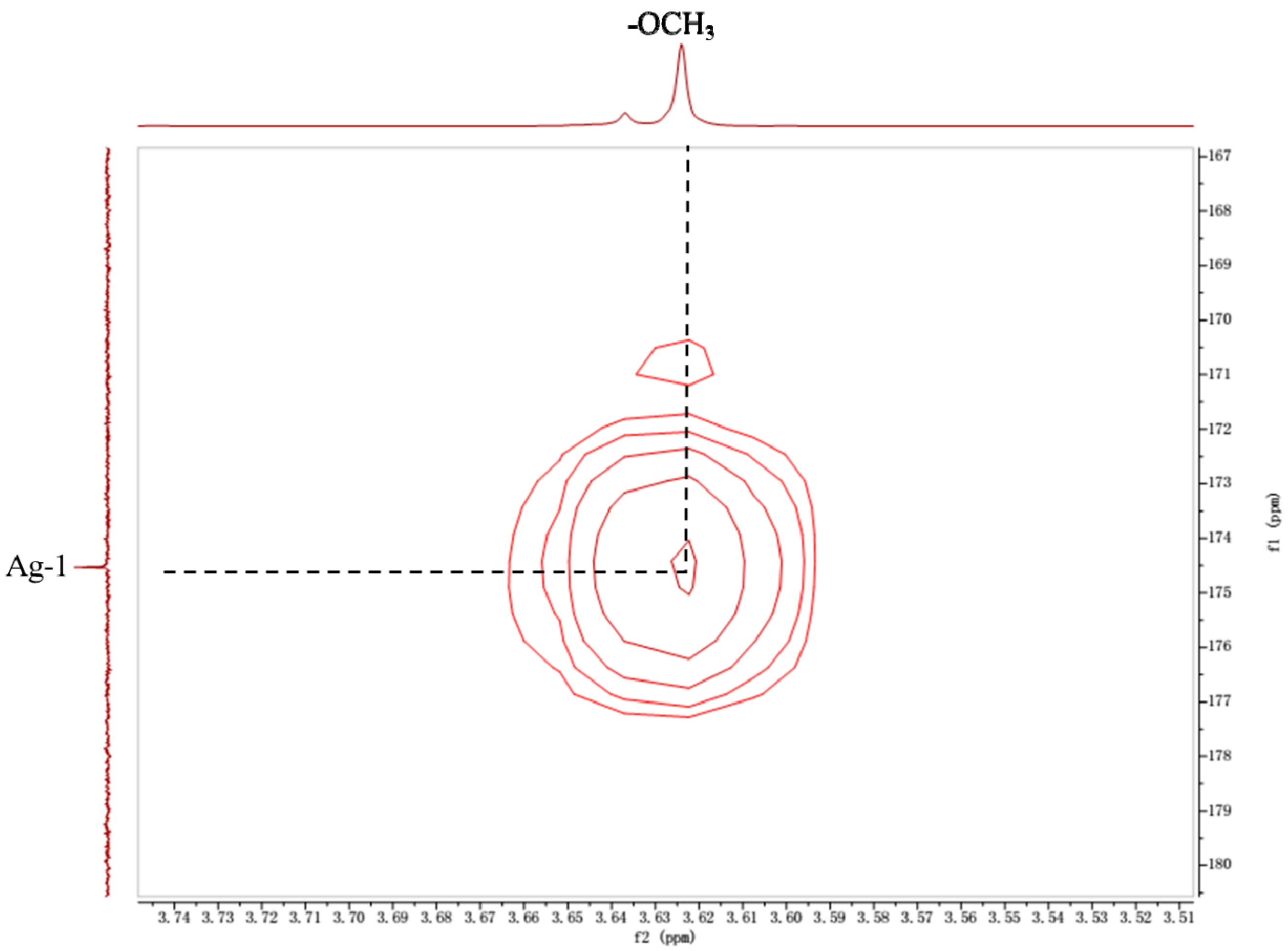

Figure S90. Expanded HMBC spectrum on the ester linkages of compound **8** (600 MHz, pyridine-*d*5)

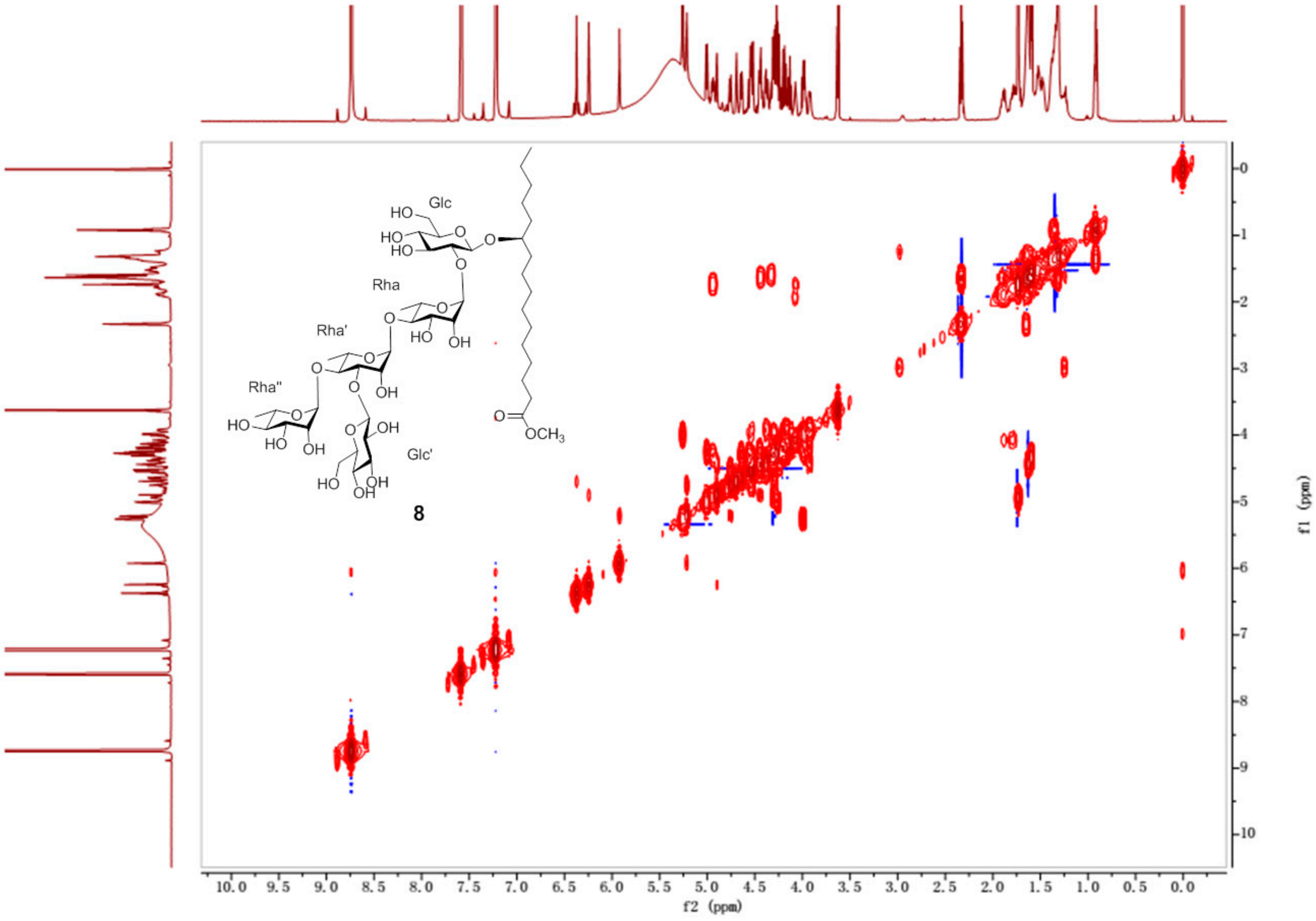

Figure S91. ^{1}H-^{1}H COSY spectrum of compound **8** (600 MHz, pyridine-d_5)

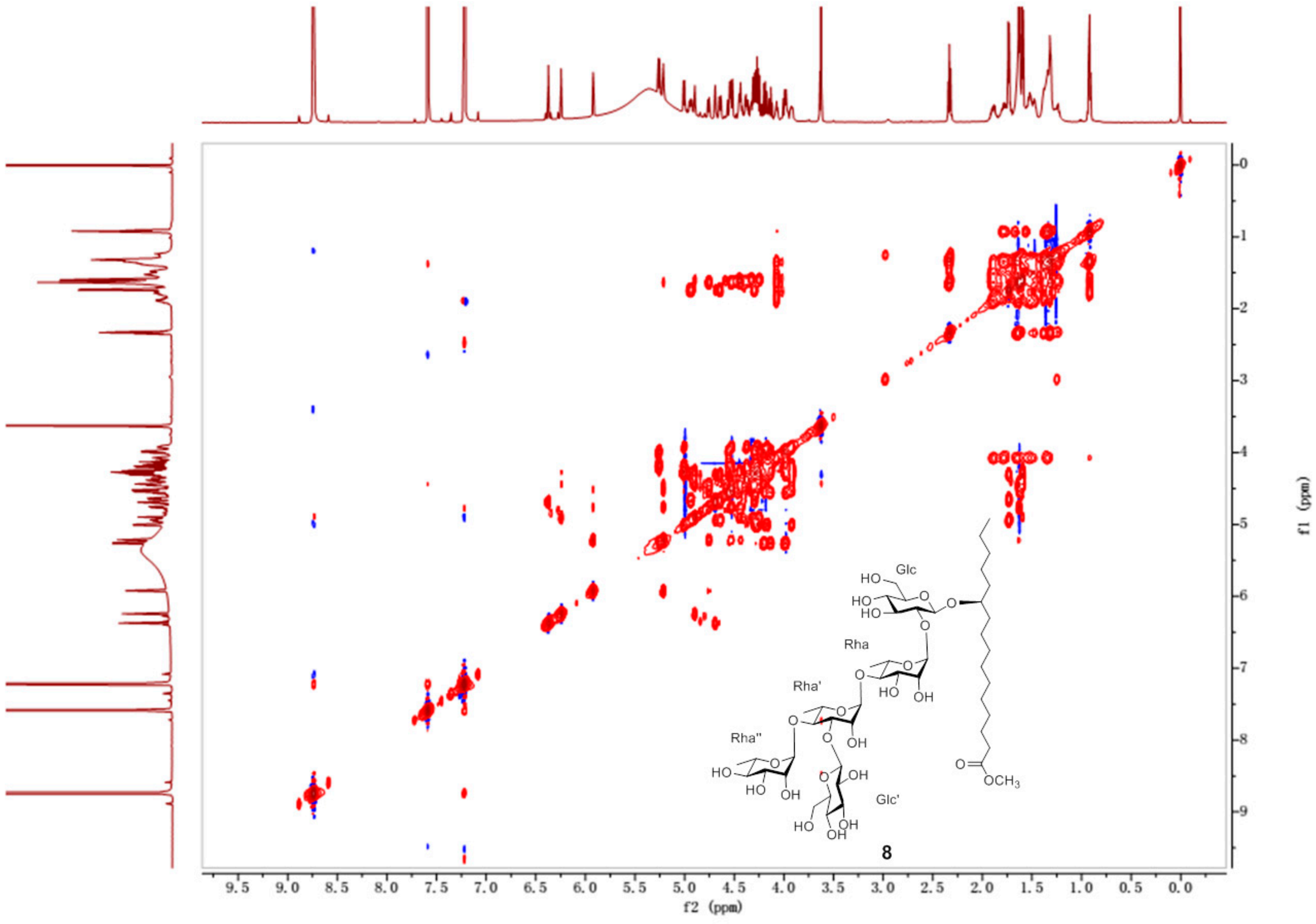

Figure S92. TOCSY spectrum of compound **8** (600 MHz, pyridine-*d*5)

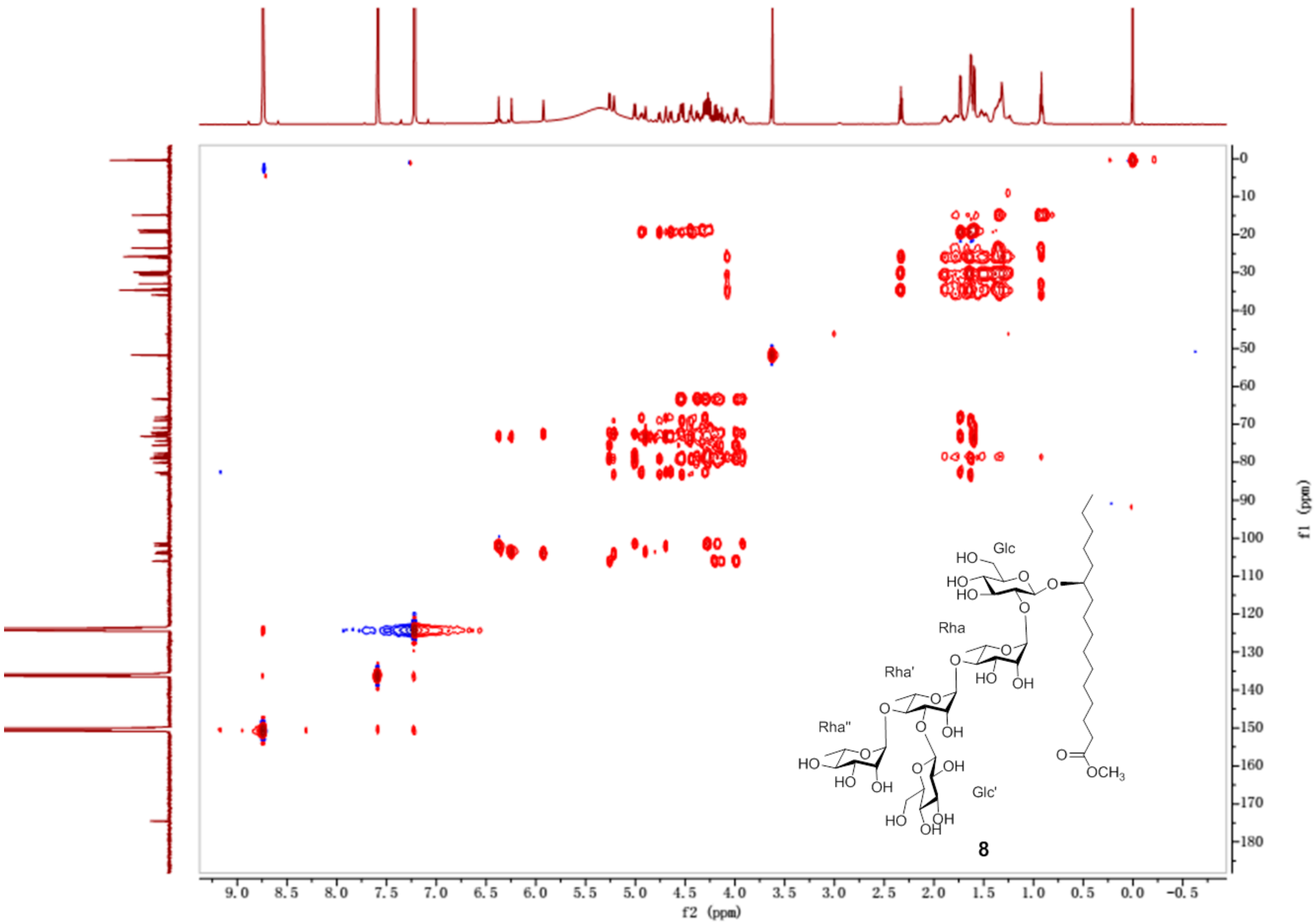

Figure S93. HSQC-TOCSY spectrum of compound **8** (600 MHz, pyridine-*d*5)

Elemental Composition Report

Single Mass Analysis

Tolerance = 5.0 mDa / DBE: min = -1.5, max = 50.0
Element prediction: Off
Number of isotope peaks used for i-FIT = 3

Monoisotopic Mass, Even Electron Ions
6525 formula(e) evaluated with 1 results within limits (up to 50 closest results for each mass)
Elements Used:
C: 47-47 H: 84-84 N: 0-100 O: 0-100 Na: 0-2

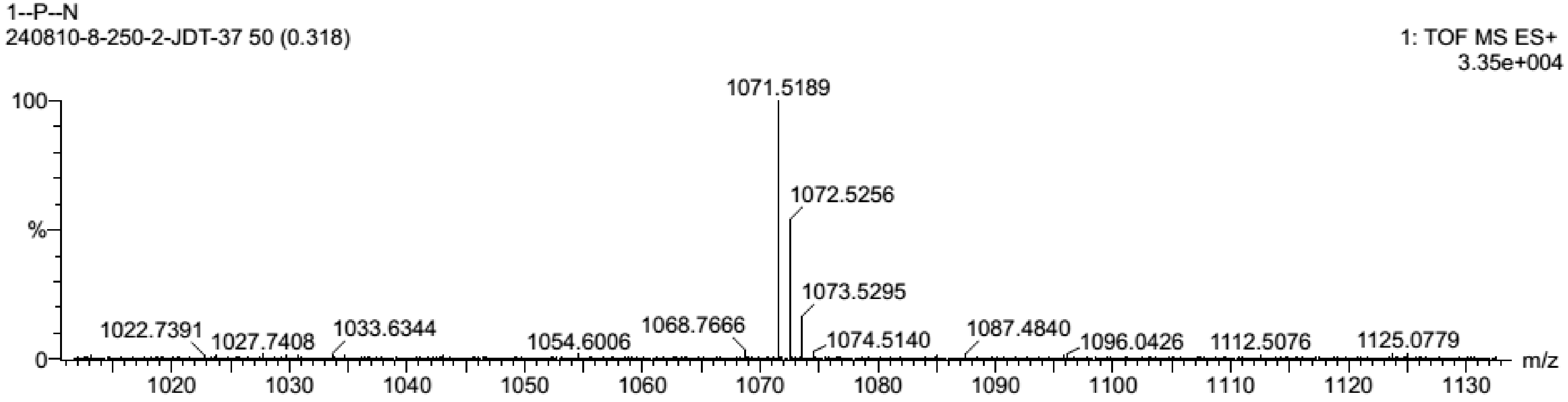

Minimum:				-1.5				
Maximum:		5.0	10.0	50.0				
Mass	Calc. Mass	mDa	PPM	DBE	i-FIT	Norm	Conf(%)	Formula
1071.5189	1071.5199	-1.0	-0.9	5.5	375.3	n/a	n/a	C47 H84 O25 Na

Figure S94. HRESIMS spectrum of compound **8**
